# The blood schizonticidal activity of tafenoquine makes an essential contribution to its prophylactic efficacy in nonimmune subjects at the intended dose (200 mg)

**DOI:** 10.1186/s12936-017-1862-4

**Published:** 2017-05-19

**Authors:** Geoffrey Dow, Bryan Smith

**Affiliations:** 60 Degrees Pharmaceuticals LLC, 1025 Connecticut Ave NW Suite 1000, Washington, DC 20036 USA

**Keywords:** Antimalarial drug, Malaria, *Plasmodium falciparum*, *Plasmodium vivax*, Tafenoquine, Primaquine, Asexual blood stages, Blood schizonticide

## Abstract

Tafenoquine (TQ) is an 8-aminoquinoline anti-malarial being developed for malaria prophylaxis. It has been generally assumed that TQ, administered prophylactically, acts primarily on the developing exoerythrocytic stages of malaria parasites (causal prophylaxis), and that polymorphisms in metabolic enzymes thought to impact the activity of other 8-aminoquinolines also inhibit this property of TQ. Furthermore, it has been suggested that a diagnostic test for CYP2D6 metabolizer status might be required. In field studies in which metabolic status was not an exclusion criteria, TQ has been shown to exhibit similar prophylactic efficacy as blood schizonticidal drugs (mefloquine). Also, its blood schizonticidal and anti-relapse efficacy is independent of 2D6 metabolizer status. The most reasonable explanation for the field study results, supported by other clinical and non-clinical data, is that TQ is not completely causal and exhibits substantial blood schizonticidal activity at the intended dose. Pharmacokinetic simulations demonstrate that trough concentrations of TQ exceed the proposed MIC of 80 ng/ml in >95% of individuals. Based on these data a companion diagnostic for CP450 enzyme status is not required.

**Electronic supplementary material:**

The online version of this article (doi:10.1186/s12936-017-1862-4) contains supplementary material, which is available to authorized users.

## Background

Tafenoquine is a long–half-life analog of primaquine being developed for travellers and community malaria prophylaxis by the United States (US) Army, 60 Degrees Pharmaceuticals, and their partners. Because of its long half-life (approximately 13 days), convenient dosing regimens, effectiveness against hypnozoites, broad spectrum activity against all species of malaria, and the absence of documented drug resistance, tafenoquine represents a potential improvement over the standard-of-care (doxycycline, atovaquone-proguanil, and mefloquine) in certain settings. However, the appropriateness of primaquine, and by inference tafenoquine, for prophylaxis in nonimmune subjects has been questioned, because of a perception that these agents may not arrest the development of 100% of the pre-erythrocytic stages of *Plasmodium falciparum* in individuals with certain enzyme deficiencies [1].

Primaquine is recommended for malaria prophylaxis at a dose of 30 mg/day [2]. Primaquine, when administered as a single dose, prevents patency in challenge studies only if it is administered within 1–3 days of a sporozoite challenge, not if it is administered before or after this time [3]. At doses higher than those used for malaria prophylaxis, primaquine clears symptomatic infections (i.e., blood stages) of *Plasmodium vivax* but not those of *P. falciparum* [4–6]. Thus, the pharmacological basis of the prophylactic efficacy of primaquine in non-immune subjects is assumed to be primarily its inhibitory effect on developing (and latent) exoerythrocytic parasites.

It has been hypothesized that a primaquine metabolite responsible for hypnozoite killing is generated by a CYP2D6-dependent pathway [7], and that, in certain individuals, inherited polymorphisms in CYP2D6 can result in insufficient levels of that metabolite, thereby exposing them to greater risk for the relapse of *P. vivax* hypnozoites following primaquine administration. Similarly, in animal models, CYP2D6 knockout mice are not protected from *Plasmodium berghei* by primaquine following a sporozoite challenge [8], which suggests a common mode of causal and hypnocytocidal action. CYP2D6 polymorphisms in humans vary by ethnicity and geography [9] and some in the field believe that travellers (up to ~13% in the case of travellers with European ancestry) with an inherited CYP2D6 deficiency may be at greater risk of contracting falciparum malaria if using primaquine for malaria prophylaxis [1, 10]. Because primaquine and tafenoquine are 8-aminoquinolines, it is also assumed that what is true for primaquine may also be true for tafenoquine; therefore, tafenoquine will only be approvable for use in patients that have tested for CYP2D6 metabolic status [1].

In fact, as will be argued in the remainder of this review, the available evidence suggests that tafenoquine exhibits substantial blood schizonticidal activity, and by virtue of its long half-life, clears asexual blood stages in animals and humans, albeit more slowly than traditional blood schizonticidal drugs. Animal studies suggest that the blood schizonticidal effect of tafenoquine is independent of cP450 2D6 enzyme status. Furthermore, because tafenoquine, inherently, may not kill all developing exoerythrocytic parasites in all individuals at the intended dose, its blood schizonticidal effect makes an important contribution to its prophylactic efficacy at the intended dose (200 mg).

## Tafenoquine and mefloquine exhibit similar prophylactic efficacy against *Plasmodium falciparum* and *Plasmodium vivax* in field studies

### Efficacy against *P. vivax*

In the pivotal Phase III study [11], 654 nonimmune Australian soldiers were randomly assigned to receive mefloquine (n = 162) or tafenoquine (n = 492) during deployment to Timor, and the *P. vivax* attack rate during the period of the deployment was retrospectively estimated to be 6.88% [11]. The subjects in this study were of largely European ethnicity, meaning that the rate of deficient CYP2D6 phenotypes may have been as high as 13% [9]. The subjects also had no prior exposure to malaria, meaning that any viable blood stage infections representing a potential risk would have been symptomatic.

The following was assumed for the purposes of conducting a thought experiment:

i. Tafenoquine exhibits no blood schizonticidal activity in humans,
ii. Tafenoquine kills no developing exoerythrocytic stages in individuals with a low CYP2D6 metabolizer phenotype,
iii. Tafenoquine kills all developing exoerythrocytic stages in a proportion of individuals with a normal CYP2D6 phenotype that is the same proportion of non-immune individuals that would be expected to be protected by a standard anti-malarial in this research setting (100% for mefloquine [11]).

It can be further assumed that the pivotal study was repeated with the same sample size in the tafenoquine arm and included a placebo group where the *P. vivax* attack rate was 6.88%. In such a study 1.7, 3.4 or 4.4 *P. vivax* cases should have been observed in the tafenoquine arm if the background rate of low metabolizer CYP2D6 phenotypes was 5, 10 or 13%, respectively.

However, in the actual Phase III study this was not the case: the point estimate of efficacy (95% confidence intervals [CI]) for tafenoquine was: 100% (93–100%).

### Efficacy against *Plasmodium falciparum*

In two Phase II studies from which the data were pooled, 519 African residents of high malaria transmission areas were randomly assigned to receive placebo (n = 187), tafenoquine (n = 190), or mefloquine (n = 142) during a period in which the incidence density in the placebo group was (almost completely attributable to *P. falciparum*) 5.06 cases/person-year [12]. Approximately 20% of individuals of sub-Saharan African ancestry may exhibit 2D6 deficient phenotypes [9].

The following was assumed for the purpose of conducting a thought experiment:

i. Tafenoquine exhibits no blood schizonticidal activity in humans,
ii. Tafenoquine kills no developing exoerythrocytic stages in individuals with a low CYP2D6 metabolizer phenotype,
iii. Tafenoquine kills all developing exoerythrocytic stages in a proportion of individuals with a normal CYP2D6 phenotype that is the same as the proportion of semi-immune individuals that would be expected to be protected by a standard anti-malarial in this research setting (94.5% for mefloquine [12]).

It can be further assumed that the pooled Phase II was conducted with the same sample sizes and a *P. falciparum* attack rate was 5.06 cases/person-year. In such a study, 22, 32 or 62 *P. falciparum* cases should have been observed in the tafenoquine arm if the background rate of low metabolizer CYP2D6 phenotypes was 5, 10 or 20%, respectively.

However, this was not the case. The observed number of cases in the actual tafenoquine arm was only 14 and the prophylactic efficacy of tafenoquine was 93.5% (89–96%) [12].

## A combination of blood schizonticidal and causal activity are required to mediate the prophylactic efficacy of tafenoquine at the intended dose

Neither of the field studies discussed above excluded any individuals who may have had alternate metabolizer status associated with CYP2D6 or any other type of metabolic enzyme. There is no evidence from field studies that the efficacy of continuous treatment-compliant prophylaxis with tafenoquine is any different from treatment-compliant prophylaxis with mefloquine. Therefore, the assumptions made in the thought experiments above must be incorrect. The field study results can be explained only if continuous prophylaxis is completely causal or if tafenoquine exhibits substantial blood schizonticidal activity against both *P. vivax* and *P. falciparum* in addition to its substantial causal activity that is unaffected by cP450 metabolizer status. In the remainder of this section the literature was reviewed to determine which one of these hypotheses is most reasonable.

### Both causal and suppressive actions of tafenoquine may be required to convey complete prophylactic activity in mice

In humans, it is not possible to definitively assess the relative contribution of causal and blood schizonticidal activity to the prophylactic efficacy of tafenoquine. However, animal models provide important clues. Li et al. [13] examined the contributions of causal and suppressive effects of tafenoquine to its prophylactic efficacy in mice utilizing a transgenic *P. berghei* parasite expressing the bioluminescent reporter protein luciferase. Drug activity against developing liver stages was assessed using a real-time in vivo imaging system and suppressive activity against erythrocytic stages was assessed using flow cytometry. When one dose of tafenoquine (5 mg/kg) was administered 1 day prior to sporozoite inoculation, merozoite leakage was not detected by flow cytometry through day 30 in 10 of 10 animals. However, in these animals real time imaging demonstrated that only 98.6% of exoerythrocytic parasites were eliminated compared to controls at 48 h after sporozoite challenge. Because parasites begin to emerge from the liver to enter the blood at 48 h in this model, some of the remaining 1.4% of liver parasites had entered the blood after that time and the 100% prophylactic efficacy of this regimen was due to elimination of a few blood parasites by tafenoquine remaining in the blood after 48 h. These data imply that at this particular dose in mice, both causal and blood schizonticidal activity are important for the prophylactic efficacy of tafenoquine. It is also true that at higher doses (e.g. 5 mg/kg/day for 3 days), 100% prophylactic efficacy was observed following 100% inhibition of liver schizogony. This means that it might also be possible in humans, assuming it could be tolerated, to administer a dose of tafenoquine at which prophylactic efficacy was mediated only via causal activity.

### Causal prophylactic effect of tafenoquine 600 mg against *P. falciparum* is not complete

**Fig. 1:**
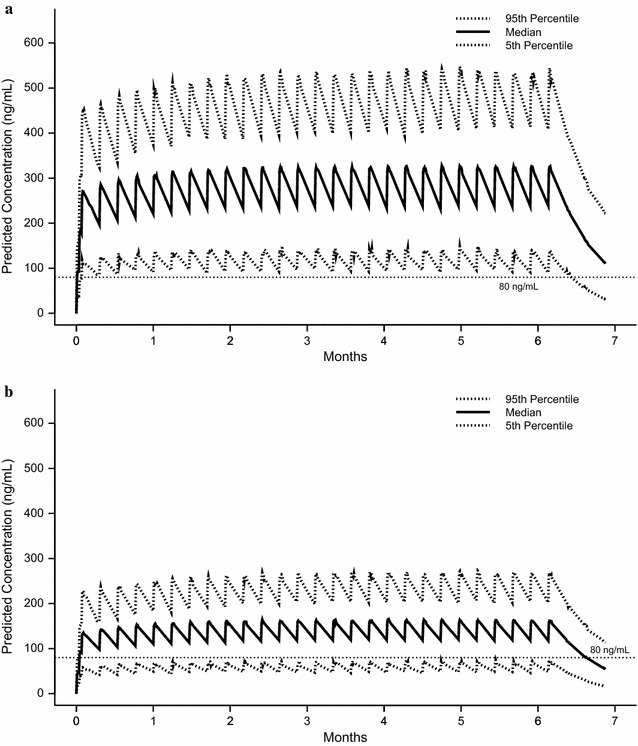
Plasma-concentration time curves for selected regimens of tafenoquine. Plasma-concentration time curves for tafenoquine generated from pharmacokinetic simulations at doses of 200 mg (**a**) and 100 mg (**b**) administered once per day for 3 days (load) then once per week for a simulated deployment of 6 months. Median trough concentrations exceed the putative MIC of 80 ng/ml for both regimens, but trough levels for the 200 mg dose exceed the MIC in 95% of subjects. The simulations utilized pharmacokinetic parameters from a US Army study [16] which were derived from modeling of data from eleven published and unpublished pharmacokinetic studies

The first challenge study involving tafenoquine attempted to position the drug as a causal prophylactic. Four nonimmune healthy volunteers were given a single 600-mg dose immediately prior to a mosquito-induced *P. falciparum* challenge [14]. The intended dose of a 200 mg × 3 load followed by 200 mg weekly essentially maintains this exposure level of tafenoquine at steady state. Three of the four volunteers were protected. The fourth contracted symptomatic malaria on day 31 after the challenge and had drug levels approximately half those of the three protected subjects. Because the study was conducted more than 15 years ago and there was no reason to suspect a correlation between cytochrome P450 enzyme status and activity at the time, the metabolic enzyme profile of the subject who contracted malaria is not known. Nevertheless, the Cmax observed in the single failure (244 ng/ml) was <15% lower than the predicted median Cmax following the intended loading dose of 200 mg × 3 for malaria prophylaxis (~280 ng/ml, see Fig. 1; Table 1). Assuming this effect is generalizable, exposure in humans at the intended dose may produce a pharmacologic effect more similar to that observed following the lower dose used in the mouse studies above. Therefore, the efficacy of continuous chemoprophylaxis can only be explained by a substantial blood schizonticidal effect.

**Table 1:** Summary of key concentration-related pharmacodynamic endpoints for tafenoquine

Actual or estimated concentration parameter of interest (ng/ml)	Regimen					
	0.6, 2 and 6 mg/kg/day × 3 against Pc in Rhesus monkeys [15] (HED = 36, 120 and 360 mg/day)^a^	1 and 3 mg/kg/day × 3 against Pv in Aotus monkeys [17] (HED = 60 and 180 mg/day)^a^	600 mg × 1 in nonimmune patients [14]	200 mg/day × 3 + 200 mg once per week in nonimmune and semi-immune subjects (see also Fig. 1, [16, 18])	100 mg/day × 3 + 100 mg once per week (hypothetical) (see also Fig. 1, [16])	400 mg/day × 3 + 400 mg once per month in Thai soldiers [18, 19]
Cmax	50, 150 and 500	~90 and 250	400	280	140	730
Steady state trough (median)	NA	NA	NA	220	110	Month 1–238^b^ Month 6–104^b^
Steady state trough (5%)	NA	NA	NA	90	45	NA
Steady state Cmax	NA	NA	NA	320	160	~350–500
Major pharmacologic effects and/or concentration at failure or relapse	2 and 6 mg/kg cleared established infection, no recrudescense at 6 mg/kg	1 and 3 mg/kg cleared established infection, no recrudescence at 3 mg/kg	Pf = 49 at day 31, Cmax in failed subject was 244 ng/ml	No Pf or Pv failures during deployment in non-immunes with 4 post-deployment Pv relapses—concentration unknown Similar efficacy to mefloquine against Pf in semi-immune subjects	NA	Pv = 20, 21 and Pf = 39 at 6–12 weeks after dosing Pv = 38 in subject non-compliant with medication
In vitro parameters	IC_50_ 97 in 44 MDR Pf strains from Thai/Cambodian border [22] IC_99_ 3108 in 44 MDR Pf strains from Thai/Cambodian border [22] IC_50_ 64-110 in 2 MDR Southeast Asia Pf strains [23] IC_50_ 2041 in 166 African Pf strains [24] IC_50_ 202 in seven MDR laboratory strains [25]					

*C*_*max*_ maximum concentration, *MDR* multidrug resistant, *NA* not available, *NR* not reported, *Pv Plasmodium vivax*, *Pf Plasmodium falciparum*, *Pc Plasmodium cynomolgi*, *HED* human equivalent dose assuming a 65 kg subject

^a^HED calculated assumed conversion factor 0.916 from a mg/kg dose in monkeys to a mg dose in a 65 kg human. The conversion factor was derived based on an observed median Cmax of 50 ng/ml in Rhesus monkeys at a dose 0f 0.6 mg/kg/day for 3 days versus a simulated Cmax of 280 ng/ml following a 200 mg × 3 loading dose in a 65 kg human and assuming Cmax scales linearly with dose. The same conversion factor between Rhesus monkeys and humans was assumed for Aotus monkeys

^b^Not at steady state; trough concentrations declined

### Evidence for a blood schizonticidal effect of tafenoquine from non-clinical and clinical studies

Several lines of evidence suggest that tafenoquine acts as a slow-acting suppressive prophylactic against both animal and human *Plasmodium* species at relevant doses.

First, tafenoquine administered at doses ≥2 mg/kg/day for 3 days cleared the erythrocytic stages of *Plasmodium cynomolgi* in *Macaca* monkeys within 3–4 days and at doses of 6 mg/kg/day for 3 days there was no relapse or recrudescence [15]. Dow et al. reported a median Cmax of approximately 50 ng/ml following administration of 0.6 mg/kg/day for 3 days [15]. Assuming pharmacokinetic parameters are proportional to dose, the median Cmax at the minimum dose (2 mg/kg/day × 3) was 150 ng/ml. This is higher than the lowest 5% Cmax observed following administration of the intended human loading dose (~120 ng/ml, Fig. 1 [16]), meaning it would be expected that the intended loading dose in humans would clear an established blood stage infection. The rate of parasite clearance observed at 2 mg/kg/day was slower than that for a comparator group administered chloroquine and a lower dose of tafenoquine. This means that tafenoquine clears asexual parasitaemia more slowly than established blood schizonticidal drugs.

Second, in *Aotus* monkeys, doses of 1 mg/kg/day for 3 days cleared the erythrocytic stages of an established chloroquine-resistant *P. vivax* with recrudescence 20–25 days later [17]. Doses of 3 mg/kg/day for 3 days were required for complete elimination (cure). No pharmacokinetic data are available from that study. However, let us assume that the conversion factor calculated to derive a human equivalent dose (HED) from *Macaca* monkeys that is presented in Table 1, is the same for *Aotus* monkeys. Therefore, at an HED of 120 mg per day × 3, tafenoquine is able to clear an established chloroquine-resistant *P. vivax* infection, while at a dose of around 360 mg per day × 3, the drug is able to cure such an infection. This confirms that clearance of established *P. vivax* infections should be possible at the anticipated loading dose in humans.

Third, Brueckner et al. [14], in the same challenge study discussed earlier, observed blood stage parasites following sporozoite challenge in one individual on day 31 after that individual had received a single 600-mg dose of tafenoquine. Drug levels at day 31 were approximately 48 ng/ml. Normal patency following a *P.* *falciparum* challenge is not usually longer than day 11. A reasonable interpretation of this data is that tafenoquine at concentrations greater than 48 ng/ml are able to suppress the amplification of low-density *P. falciparum* infections.

Fourth, Edstein et al. [18], in a study conducted in Thai soldiers, reported 3 symptomatic breakthroughs for which drug levels were measurable occurred 6–12 weeks after prophylaxis: two *P. vivax* cases with drug levels of 20 and 21 ng/ml and a single case of *P. falciparum* with a tafenoquine concentration of 38 ng/ml (Table 1). During the prophylactic phase of the same study, a single symptomatic *P. vivax* breakthrough was observed in an individual noncompliant with the medication, and the tafenoquine concentration was determined to be 40 ng/ml [19]. These data are consistent with the hypothesis that tafenoquine concentrations in excess of 40 ng/ml are able to control low density infections caused by multiply drug resistant *P. falciparum* and *P. vivax*.

Finally, tafenoquine exhibits greater potency against the blood stages than primaquine in vivo. Against the erythrocytic stages of drug-sensitive strains of *P. berghei* in vivo, tafenoquine is 9 times more active than primaquine [20]. Against the erythrocytic stages of strains of *P. berghei* resistant to conventional anti-malarial drugs, tafenoquine is 4 to 100 times more potent than primaquine in vivo [20]. This supports the hypothesis that tafenoquine should exhibit more activity against *P. falciparum* in vivo in humans than primaquine.

Together, these in vivo studies suggest that tafenoquine is able to clear established (high density) blood stage infections at exposure levels equivalent to the intended loading dose, and to control sub-patent low density infections at relatively low drug concentrations (>50 ng/ml) in vivo.

### Tafenoquine exhibits a 2D6-independent blood schizonticidal effect against *P. berghei* in mice

In a recent study, Milner et al. [21] demonstrated that a 20 mg/kg oral dose of tafenoquine administered exhibited different pharmacokinetic parameters in wild type and 2D6 knockout mice. However, in the same study, the authors also demonstrated that a single dose of 25 mg/kg tafenoquine, administered 4 days following a sporozoite inoculation, cleared asexual parasitaemia in wild type and 2D6 knock out with no difference in time to clearance or course of parasitaemia. These data demonstrate that although it is feasible that defects in 2D6 metabolizer status might affect the pharmacokinetic properties of tafenoquine, the drug is perfectly able to clear established blood stage infections in the absence of a functional CP450 2D6 cluster. Of note, similar effects were observed for primaquine, which implies a class effect.

### Biological basis of tafenoquine prophylaxis and lack of a requirement for a cP450 2D6 companion diagnostic

The most parsimonious explanation for all the available data is that continuous tafenoquine prophylaxis at the intended dose protects non-immune individuals from contracting symptomatic malaria by (i) eliminating most, but not all, developing asexual exoerythrocytic stages following an infectious mosquito bite, and (ii) slowly clearing the few erythrocytic stages that leak out of the liver in some individuals in a manner that is independent of cP450 2D6 metabolizer status. There is, therefore, no need for a cP450 2D6 companion diagnostic.

## Evidence gaps and alternative explanations

### Malaria parasites are more susceptible to tafenoquine in vivo than in vitro and the mechanism of action against blood stage parasites is not known

Based on the association of symptomatic breakthroughs with measured plasma concentration discussed above, Edstein et al. [18] proposed that the minimum inhibitory concentration (MIC) of tafenoquine required to protect nonimmune individuals from a symptomatic breakthrough is approximately 80 ng/ml. The US Army/commercial team developing tafenoquine has modelled all published and internal pharmacokinetic data to model the plasma concentrations of tafenoquine at the intended and lower doses [16]. The current thinking regarding the dose justification for malaria prophylaxis is that the intended dose of 200 mg × 3 followed by weekly maintenance doses generates trough concentrations in excess of the proposed MIC throughout the course of administration in 95% of individuals, whereas lower doses (e.g., a 100-mg regimen) do not (Table 1; Fig. 1).

However, this does not seem to make sense based on in vitro susceptibility data, since the IC50s, and most importantly the IC90/99s, reported for tafenoquine, are generally higher than the proposed MIC in vivo. Ramharter et al. reported that tafenoquine exhibits a mean IC_50_ of 97 ng/ml and a mean IC_99_ of 3108 ng/ml against 44 multiple-drug-resistant strains of *P. falciparum* from the Thai-Burmese border in a schizont maturation assay [22]. Ohrt et al. [23] reported an IC_50_ of 64 to 110 ng/ml against 2 drug resistant strains of *P. falciparum* from South East Asia. Tafenoquine was found to exhibit an IC_50_ of 2041 ng/ml amongst 162 African isolates of *P. falciparum* [24]. Vennerstrom et al. [25] reported a mean IC50 of 202 ng/ml against seven drug-resistant clones of *P. falciparum*.

The counter argument is that endpoints measured in vitro, and the conditions utilized to generate them are vastly different from the relevant in vivo pharmacodynamics endpoints for malaria prophylaxis. First, the in vitro assays conducted to date all exhibit short exposure formats (48 h) relative to the parasite clearance time observed in vivo. Second, in vitro assays executed to date utilize much higher initial parasite densities than those after initial merozoite release from the liver in a nonimmune subject who has been administered prophylactic treatment. Third, the in vitro assays conducted do not account for either (i) any metabolic activation outside red blood cells (if this is required for activity) and (ii) cannot replicate possible deleterious effects of tafenoquine on parasites that survive exposure in the liver but escape into the blood stream. In fact, it may not be possible to replicate all the relevant in vivo parameters in an in vitro assay system.

It should be stated that the mechanism(s) of action of tafenoquine against blood stage parasites are not known, and come from a single study reported by Vennerstrom et al. [25]. In that study, Vennerstrom et al. [25] proposed two possible mechanisms of action.

First, tafenoquine, but not primaquine, was shown to inhibit haem polymerization in vitro at concentrations lower than chloroquine (16 v 80 microM [25]). However, as the authors themselves point out, it cannot be concluded that this mechanism is relevant without data showing that tafenoquine accumulates in the food vacuole to the requisite degree at relevant concentrations. Furthermore, this seems implausible since only moderate inhibition is observed in vitro in most parasites lines at concentrations higher than the median Cmax of the intended loading dose (~280 ng/ml, see Fig. 1; Table 1).

Second, Vennerstrom et al. [25] also proposed that 8-aminoquinolines might exhibit their blood schizonticidal effects through oxidative mechanisms. This seems somewhat more plausible since it is well known that 8-aminoquinolines increase oxidative stress in red blood cells with reduced G6PD leading to increased deformability and enhanced splenic and hepatic clearance [26, 27]. A reasonable topic for further study would be to assess whether, in vivo, 8-aminoquinolines, via oxidative mechanisms, increase the deformability of infected red blood cells which are then cleared by the spleen and/or liver.

### No direct evidence of clearance of *P. falciparum* in humans

The existing clinical efficacy database does not include any studies that directly show clearance of *P. falciparum* in non-immune subjects. The lack of availability of such data has largely been for ethical concerns due to the slow parasite clearance time of tafenoquine. However, two approaches that could generate such data can be envisioned. The first is utilization of a well-characterized blood stage challenge model. Such a model would avoid the confounding effect of the causal activity of tafenoquine that would be encountered with a sporozoite challenge. Additionally, the parasite density following inoculation is more in line with the density that would be encountered after merozoite release during prophylaxis. The second approach would be, in a treatment study enrolling *P. falciparum* patients, administration of tafenoquine in combination with a dose of artesunate that clears but does not cure. Tafenoquine should clear up the residual parasite burden in a manner analogous to that for mefloquine.

## A side note on the role of cP450 2D6 polymorphisms in prevention of *P. vivax* relapse by tafenoquine

As discussed in the introductory paragraphs, it appears as if the causal prophylactic efficacy of primaquine in animals, and anti-relapse efficacy against *P. vivax* in humans requires 2D6 activation. This implies a common mode of action of killing of *P. vivax* hypnozoites and the exoerythrocytic schizonts of *P. falciparum* by primaquine. Animal data suggest that 2D6 deficiency also arrests the causal activity of tafenoquine. However, St Jean et al. [28] did not find a correlation between the frequency of *P. vivax* relapse and incidence of cP450 2D6 intermediate metabolizer status, implying different modes of causal and hypnocytocidal action for tafenoquine. However, these data should be treated cautiously until additional information becomes available. The St Jean et al. study contained few poor metabolizers [28]. Also, the incidence of relapse may be masked in long half-life drugs with blood schizonticidal activity such as tafenoquine. In any case, from a clinical stand-point, it makes no difference. The anti-relapse efficacy of the intended doses for radical cure (300 mg) and prophylaxis (200 mg × 3 then 200 mg weekly) exhibit the same anti-relapse efficacy as the standard of care (primaquine) with a simpler dosing regimen [12, 29, 30], so no cP450 2D6 diagnostic test is required.

## Conclusions

### Future use of tafenoquine for malaria prophylaxis

Concern has been expressed in the literature regarding the use of tafenoquine for malaria prophylaxis due to the assumption that (i) it, like primaquine, is not 100% causal in individuals with certain cytochrome P450 enzyme deficiencies and (ii) that it exhibits little blood schizonticidal activity. However, although tafenoquine was not universally causally prophylactic in a challenge study following a single dose, it exhibits prophylactic efficacy following continuous dosing that is equivalent to that of mefloquine (a blood schizonticidal drug) in field studies in which study subjects were not excluded on the basis of metabolic enzyme status. It appears plausible based on non-clinical and clinical efficacy data and pharmacokinetics that the robust efficacy of the drug is due to a slow-acting blood schizonticidal effect in addition to its substantial although incomplete causal prophylactic effect. This effect, in animals, is independent of cP450 2D6 isoenzyme status, which suggests that, other than the routine testing required for G6PD deficiency, no companion diagnostic test is required. Additional studies to demonstrate directly clearance of *P. falciparum* in non-immune subjects, and further mechanistic studies would be helpful, though not essential for a complete understanding of tafenoquine’s prophylactic effect.

It has been proposed that tafenoquine be administered by mass treatment to more rapidly eliminate malaria in endemic countries [31]. Dow et al. have proposed that this should not be conducted using single-dose administration, but with periodic dosing during peak malaria transmission season amongst all those in the community who are willing and able to take tafenoquine [12]. Such a regimen would be administered in addition to existing malaria control measures. Because subjects would be asymptomatic and tafenoquine would be providing “temporary immunity,” the community benefit may be greater than the individual benefit. In this context, the absolute magnitude of the prophylactic effect of tafenoquine and the manner in which it mediates such an effect is not particularly relevant, because the goal is to add to, not to replace, the standard of care. Therefore, as with travel medicine, diagnostic screening, other than currently required screening for G6PD deficiency, should not be required.

## Additional file

**Additional file 1.** Population Pharmacokinetic Modeling Report.

## Abbreviations

CI: confidence interval
MIC: minimum inhibitory concentration
US: United States
