# Supplementary material for: The blood schizonticidal activity of tafenoquine makes an essential contribution to its prophylactic efficacy in nonimmune subjects at the intended dose (200 mg)
Source: Malar J. 2017 May 19;16:209. doi: 10.1186/s12936-017-1862-4 (PMC5438551; doi:10.1186/s12936-017-1862-4)

Population Pharmacokinetic Modeling Report

**Drug Name:**	Tafenoquine
**Title:**	Population Pharmacokinetic Modeling of Tafenoquine

**Compound:**	Tafenoquine
**Sponsor:**	USAMMDA

**Report Version:**	Final
**Report Date:**	28 June 2013

TABLE OF CONTENTS

PAGE

PART I. CONFIRMATORY POPULATION PK MODELING OF 044 AND 033

1. SUMMARY 16
2. INTRODUCTION 21
3. OBJECTIVES 21
4. METHODS 21
   4.1. Study Design 21
      4.1.1. Pharmacokinetic Collection 22
   4.2. Data Assembly 22
      4.2.1. Description of Input Data 22
      4.2.2. Handling of Input Data 22
      4.2.3. Bioanalytical Methodology 23
   4.3. Data Analysis 23
   4.4. Modeling Assumptions 24
      4.4.1. Description of PK Model 24
      4.4.2. Variability Models 25
         4.4.2.1. Model for inter-individual variability 25
         4.4.2.2. Model for residual variability 25
         4.4.2.3. Model for interoccasion variability 26
      4.4.3. Covariate Analysis 26
   4.5. Model Evaluation 27
      4.5.1. Bootstrapping 27
      4.5.2. Posterior predictive check 27
5. RESULTS 28
   5.1. Study 044 28
   5.2. Study 033 33
6. DISCUSSION AND CONCLUSIONS 38
   6.1. Discussion 38
   6.2. Conclusions 38
7. REFERENCES 39

PART II. POOLED POPULATION PK MODELING

8. SUMMARY 41
9. INTRODUCTION 48
10. OBJECTIVES 48
11. METHODS 48
11.1. Study Design 48
11.2. Data Assembly 52
11.2.1. Description of Input Data 52
11.2.2. Handling of Input Data 52
11.2.3. Bioanalytical Methodology 52
11.3. Data Analysis 52
11.4. Modeling Assumptions 53
11.4.1. Description of PK Model 53
11.4.2. Variability Models 54
11.4.2.1. Model for inter-individual variability 54
11.4.2.2. Model for residual variability 54
11.4.3. Covariate Analysis 55
11.5. Model Evaluation 55
11.5.1. Bootstrapping 56
11.5.2. Posterior predictive check 56
11.6. Changes in Planned Analyses 56
12. RESULTS 57
12.1. Analysis Population and Data Characteristics 57
12.2. Modeling Results 57
12.2.1. Base PK Model 57
12.2.2. Final PK Model 59
12.3. Model Evaluation Results 64
12.3.1. Bootstrapping 64
12.3.2. Posterior Predictive Check Results 65
13. SIMULATIONS 71
14. DISCUSSION AND CONCLUSIONS 84
14.1. Discussion 84
14.2. Conclusions 85
15. REFERENCES 86
16. END-OF-TEXT TABLES AND FIGURES 87

LIST OF IN-TEXT TABLES

PAGE

Table 1-1 Published Population PK Parameters of Final PK Model for Study 044........17

Table 1-2 Estimated Population PK Parameters of Final PK Model for Study 044........18

Table 1-3 Published Population PK Parameters of Final PK Model for Study 033........18

Table 1-4 Estimated Population PK Parameters of Final PK Model for Study 033........19

Table 4-1 Model Development for Study 044[1] 23

Table 4-2 Model Development for Study 033[2] 24

Table 5-1 Published and Modeled Development of Structural and Covariate Population PK Model for Study 044...28

Table 5-2 Published Population PK Parameters of Final PK Model for Study 044........28

Table 5-3 Estimated Population PK Parameters of Final PK Model for Study 044........29

Table 5-4 Published and Modeled Development of Structural and Covariate Population PK Model for Study 033...33

Table 5-5 Published Population PK Parameters of Final PK Model for Study 033........34

Table 5-6 Estimated Population PK Parameters of Final PK Model for Study 033.......35

Table 8-1 Population PK Parameters of Final PK Model 42

Table 11-1 Clinical Studies of Tafenoquine 49

Table 12-1 Number of Subjects/Patients in the Population PK Analysis Population by Study...57

Table 12-2 Description and Evaluation of Key PK Models Tested 58

Table 12-3 Description and Evaluation of Key Individual Covariate PK Models Tested..60

Table 12-4 Description and Evaluation of Key Individual Covariate PK Models Tested..61

Table 12-5 Population PK Parameters of Final PK Model 62

Table 12-6 List of Diagnostic Plots for the Final PK Model 63

Table 12-7 Comparison of Bootstrap and NONMEM Parameter Estimates....................65

Table 12-8 Comparison of Bootstrap and NONMEM Estimates of Random Effects 65

LIST OF IN-TEXT FIGURES

PAGE

Figure 1-1 One-Compartment Pharmacokinetic Model 17

Figure 4-1 One-Compartment Pharmacokinetic Model 24

Figure 5-1 Published (A) and Modeled (B) Predicted (PRED) Concentration vs. Observed (DV) Tafenoquine Concentrations for Study 044 (Linear Scale)...30

Figure 5-2 Published (A) and Modeled (B) Weighted Residuals (WRES) vs. Predicted (PRED) Tafenoquine Concentrations for Study 044 (Linear Scale)..........31

Figure 5-3 Published (A) and Modeled (B) Weighted Residuals (WRES) vs. Subject number for Study 044 (Linear Scale)..........32

Figure 5-4 Published (A) and Modeled (B) predicted vs. Observed Concentrations of Tafenoquine (Linear Scale) 36

Figure 8-1 Individual Predicted (IPRED) vs. Observed (DV) Tafenoquine Concentrations (Linear and Logarithmic Scales) 44

Figure 8-2 Population Predicted (PRED) vs. Observed (DV) Tafenoquine Concentrations (Linear and Logarithmic Scales) 45

Figure 8-3 Model-predicted vs. Observed Concentrations of Tafenoquine 46

Figure 12-1 Model-Predicted vs. Observed Tafenoquine Concentration versus Time Profiles (Final PK Model)..........66

Figure 13-1 Predicted Tafenoquine Concentrations versus Time after 3 X 200 mg Once-Daily Loading Doses Followed by 200 mg Once-Weekly for Approximately 6 Months (Reference Profile) 73

Figure 13-2 Predicted Tafenoquine Concentrations versus Time after 200 mg Once-Weekly for Approximately 6 Months (No Loading Dose) (Test Profile 1) 74

Figure 13-3 Predicted Tafenoquine Concentrations versus Time after 3 X 100 mg Once-Daily Loading Doses Followed by 100 mg Once-Weekly for Approximately 6 Months (Test Profile 2)..........75

Figure 13-4 Predicted Tafenoquine Concentrations versus Time after 3 X 200 mg Once-Daily Loading Doses Followed by 200 mg Once-Weekly for Approximately 5 and Half Months followed by 3 X 200 mg Once-Daily Doses (Test Profile 3)..........76

Figure 13-5 Predicted Tafenoquine Concentrations versus Time after 3 X 200 mg Once-Daily Loading Doses Followed by 200 mg Once-Weekly for Approximately 5 and Half Months followed by 2 X 200 mg Once-Daily Doses (Test Profile 4)..........77

Figure 13-6 Predicted Tafenoquine Concentrations versus Time after 3 X 400 mg Once-Daily Loading Doses (Test Profile 5)....78

Figure 13-7 Predicted Tafenoquine Concentrations versus Time after 3 X 400 mg Once-Daily Loading Doses Followed by 400 mg Once-Weekly for Approximately 6 Months (Test Profile 6)....79

Figure 13-8 Predicted Tafenoquine Concentrations versus Time after 3 X 400 mg Once-Daily Loading Doses Followed by 400 mg Once-Monthly for Approximately 6 Months (Test Profile 7)....80

Figure 13-9 Predicted Tafenoquine Concentrations versus Time after Reference Profile versus Test Profile 3....81

Figure 13-10 Predicted Tafenoquine Concentrations versus Time after Reference Profile versus Test Profile 5 (Fasted) 82

Figure 13-11 Comparison of Median Predicted Reference Profile versus Test Profiles 83

LIST OF END-OF-TEXT TABLES

PAGE

End-of-Text Table 15-1 Data Item Definitions for NONMEM PK Input File 87

End-of-Text Table 15-2 Population PK Parameters of Base PK Model.......................90

End-of-Text Table 15-3 Population PK Parameters of Final PK Model 90

End-of-Text Table 15-4 Demographic Summary of Subjects/Patients Used in the Population PK Analysis - Tafenoquine Population PK Modeling -Overall...91

End-of-Text Table 15-5 Demographic Summary of Subjects/Patients Used in the Population PK Analysis – Army Study No. 1..........................92

End-of-Text Table 15-6 Demographic Summary of Subjects/Patients Used in the Population PK Analysis – Army Study No. 2..........................93

End-of-Text Table 15-7 Demographic Summary of Subjects/Patients Used in the Population PK Analysis – Army Study No. 3..........................94

End-of-Text Table 15-8 Demographic Summary of Subjects/Patients Used in the Population PK Analysis – Army Study No. 4..........................95

End-of-Text Table 15-9 Demographic Summary of Subjects/Patients Used in the Population PK Analysis – Army Study No. 5..........................96

End-of-Text Table 15-10 Demographic Summary of Subjects/Patients Used in the Population PK Analysis – Army Study No. 14........................97

End-of-Text Table 15-11 Demographic Summary of Subjects/Patients Used in the Population PK Analysis – Army Study No. 15........................98

End-of-Text Table 15-12 Demographic Summary of Subjects/Patients Used in the Population PK Analysis – Army Study No. 33........................99

End-of-Text Table 15-13 Demographic Summary of Subjects/Patients Used in the Population PK Analysis – Army Study No. 44......................100

End-of-Text Table 15-14 Demographic Summary of Subjects/Patients Used in the Population PK Analysis – Army Study No. 58......................101

LIST OF END-OF-TEXT FIGURES

PAGE

End-of-Text Figure 15-1 Plot of Observed (DV) vs. Individual Predicted (IPRED) tafenoquine Concentrations (Final PK Model) 102

End-of-Text Figure 15-2 Plot of Observed (DV) vs. Population Predicted (PRED) Tafenoquine Concentrations (Final PK Model)....................103

End-of-Text Figure 15-3 Plot of Residuals (RES) vs. Population Predicted (PRED) Tafenoquine Concentrations (Final PK Model)....................104

End-of-Text Figure 15-4 Plot of Residuals (RES) vs. Time (Final PK Model).............105

End-of-Text Figure 15-5 Plot of Residuals (RES) vs. Time after Dose (Final PK Model)..106

End-of-Text Figure 15-6 Plot of Weighted Residuals (WRES) vs. Population Predicted (PRED) Tafenoquine Concentrations (Final PK Model)..107

End-of-Text Figure 15-7 Plot of Weighted Residuals (WRES) vs. Time (Final PK Model)..108

End-of-Text Figure 15-8 Plot of Weighted Residuals (WRES) vs. Time after Dose (Final PK Model) 109

End-of-Text Figure 15-9 Plot of Weighted Residuals (WRES) vs. ID (Final PK Model)..110

End-of-Text Figure 15-10 Plot of Individual Weighted Residuals (IWRES) vs. Individual Predicted (IPRED) Tafenoquine Concentrations (Final PK Model) 111

End-of-Text Figure 15-11 Plot of Predicted Tafenoquine Concentrations vs. Observed Tafenoquine Concentrations (Final PK Model)....112

End-of-Text Figure 15-12 Plot of Conditional Weighted Residuals (CWRES) vs. Population Predicted (PRED) Tafenoquine Concentrations (Final PK Model) 113

End-of-Text Figure 15-13 Plot of Conditional Weighted Residuals (CWRES) vs. Time (Final PK Model) 114

End-of-Text Figure 15-14 Plot of DV, PRED, and IPRED vs. Time (Linear Scale) (Final PK Model) 115

End-of-Text Figure 15-15 Plot of DV, PRED, and IPRED vs. Time (Semi-Logarithmic Scale) (Final PK Model) 116

End-of-Text Figure 15-16 Plot of DV, PRED, and IPRED vs. Time after Dose (Linear Scale) (Final PK Model) 117

End-of-Text Figure 15-17 Plot of DV, PRED, and IPRED vs. Time after Dose (Semi-Logarithmic Scale) (Final PK Model) 118

End-of-Text Figure 15-18 Plot of CWRES vs. Covariates 119

End-of-Text Figure 15-19 Distribution of Individual Estimate of ETA (Final PK Model) 122

End-of-Text Figure 15-20 Plot of Correlation Matrix of ETA (Final PK Model)..............123

End-of-Text Figure 15-21 Plot of ETA vs. Covariates 124

LIST OF ATTACHMENTS

Attachment 1 Legacy NONMEM Dataset Validation Reports

Attachment 1.1 Validation Report for 044 Dataset
Attachment 1.2 Validation Report for 033 Dataset

Attachment 2 Confirmatory Modeling for Study 044

Attachment 2.1 NONMEM Control File and Output for 044 Model 1
Attachment 2.2 NONMEM Control File and Output for 044 Model 2
Attachment 2.3 NONMEM Control File and Output for 044 Model 3
Attachment 2.4 NONMEM Control File and Output for 044 Model 4
Attachment 2.5 NONMEM Control File and Output for 044 Model 5
Attachment 2.6 NONMEM Control File and Output for 044 Model 6
Attachment 2.7 NONMEM Control File and Output for 044 Model 7
Attachment 2.8 NONMEM Control File and Output for 044 Model 8
Attachment 2.9 NONMEM Control File and Output for 044 Model 9
Attachment 2.10 NONMEM Control File and Output for 044 Model 10
Attachment 2.11 NONMEM Control File and Output for 044 Model 11
Attachment 2.12 NONMEM Control File and Output for 044 Model 12

Attachment 3 Confirmatory Modeling for Study 033

Attachment 3.1 NONMEM Control File and Output for 033 Model 1
Attachment 3.2 NONMEM Control File and Output for 033 Model 2
Attachment 3.3 NONMEM Control File and Output for 033 Model 3
Attachment 3.4 NONMEM Control File and Output for 033 Model 4
Attachment 3.5 NONMEM Control File and Output for 033 Model 5
Attachment 3.6 NONMEM Control File and Output for 033 Model 6
Attachment 3.7 NONMEM Control File and Output for 033 Model 7
Attachment 3.8 NONMEM Control File and Output for 033 Model 8
Attachment 3.9 NONMEM Control File and Output for 033 Model 9
Attachment 3.10 NONMEM Control File and Output for 033 Model 10
Attachment 3.11 NONMEM Control File for 033 Model 9 Simulation

Attachment 4 Base PK Model (Pooled Data)

Attachment 4.1 NONMEM Control File for Base PK Model (Pooled Data)
Attachment 4.2 NONMEM Output Summary for Base PK Model (Pooled Data)
Attachment 4.3 NONMEM Detailed Results for Base PK Model (Pooled Data)
Attachment 4.4 Diagnostic Plots for Base PK Model (Pooled Data)
Attachment 4.5 NONMEM Run Record for Base PK Model (Pooled Data)
Attachment 4.6 Data Checkout Plots for PK (Pooled Data)
Attachment 4.7 Demographic Summary Results (Pooled Data)

Attachment 5 Model Evaluation Results for Base PK Model (Pooled Data)

Attachment 5.1 Bootstrap Output Summary and Run Records for Base PK Model (Pooled Data)
Attachment 5.2 Histograms of Bootstrap Parameter Estimates for Base PK Model (Pooled Data)

Attachment 6 Posterior Predictive Check Results for Base PK Model (Pooled Data)

Attachment 6.1 NONMEM Control File for Base PK Posterior Predictive Check (Pooled Data)
Attachment 6.2 Posterior Predictive Check Plots for Base PK Model (Pooled Data)

Attachment 7 Key PK Models Evaluated (Pooled Data)
Attachment 7.1 NONMEM Control File for Key PK Models (Base PK Model Development)
Attachment 7.2 NONMEM Output Summary for Key PK Models (Base PK Model Development)
Attachment 7.3 NONMEM Control File and Output for Individual Covariate PK Models
Attachment 7.4 NONMEM Control File and Output for Full Covariate PK Models

Attachment 8 Final PK Model (Pooled Data)

Attachment 8.1 NONMEM Control File for Final PK Model (Pooled Data)
Attachment 8.2 NONMEM Output Summary for Final PK Model (Pooled Data)
Attachment 8.3 NONMEM Detailed Results for Final PK Model (Pooled Data)
Attachment 8.4 Diagnostic Plots for Final PK Model (Pooled Data)
Attachment 8.5 NONMEM Run Record for Final PK Model (Pooled Data)

Attachment 9 Model Evaluation Results for Final PK Model (Pooled Data)

Attachment 9.1 Bootstrap Output Summary and Run Records for Final PK Model (Pooled Data)
Attachment 9.2 Histograms of Bootstrap Parameter Estimates for Final PK Model (Pooled Data)

Attachment 10 Posterior Predictive Check Results for Final PK Model (Pooled Data)

Attachment 10.1 NONMEM Control File for PK Posterior Predictive Check (Pooled Data)
Attachment 10.2 Posterior Predictive Check Plots (Pooled Data)

Attachment 11 Simulations

Attachment 11.1 NONMEM Control File for Simulations
Attachment 11.2 NONMEM Input File for Simulations

Attachment 12 Concentration Data (Pooled Data)

LIST OF ABBREVIATIONS

ε	Residual variability
η or ETA	Between-individual variability
θ	Population parameter
ω	Variance of ETA distribution
ADDL	Additional doses
AMT	Amount of dose
AUC	Area under the plasma concentration-time curve
BID	Twice-daily
BMI	Body mass index
BQL	Below the quantification limit
CI	Confidence Interval
CL/F or CL	Apparent clearance of tafenoquine following oral administration
C_{max}	Maximum plasma concentration
CRCL	Creatinine clearance
CV	Coefficient of variation
CWRES	Conditional weighted residuals
df	Degrees of freedom
DV	Dependent variable
EPSILON	Random effect for residual variability
ETA	Random effect for inter-individual variability
EVID	Event identification number
FO	First order approximation
FOCE	First order conditional estimation
HIST	Histopatholgical analysis for presence or absence of malaria
hr	Hour(s)
IPRED	Individual predicted observations
IWRES	Individual weighted residuals
Ka	First order absorption rate constant
Kg	Kilogram
L	Liter
LC/MS	Liquid chromatography with tandem mass spectrometry
mg	Milligram(s)
mL	Milliliter(s)
ng	Nanogram(s)
NONMEM	Nonlinear Mixed Effect Modeling Software
OFV	Objective function value
OMEGA	Variance of ETA
PDx-Pop	Front end software utility for NONMEM
PHOS	Phospholipidosis
PK	Pharmacokinetic(s)
PRED	Population predicted observations
PREDPP	PRED for Population Pharmacokinetics program
QC	Quality control

QD	Once-daily
RAP	Report Analysis Plan
RES	Residuals
RSE	Relative standard error
RSEQ	Record sequence
SAS	Statistical Analysis Software
SD	Standard deviation
SIGMA	Variance of EPSILON
THETA	Fixed effect parameter
V/F or V	Apparent volume of distribution of tafenoquine following oral administration
WRES	Weighted Residuals
WT	Body weight

PART I. CONFIRMATORY POPULATION PK MODELING OF STUDIES 044 AND 033

1. SUMMARY

Study Numbers: 044 and 033

Objectives:

The objectives of this analysis were:

- to validate the NONMEM input files used in the prior analyses,
- to verify a population PK model of tafenoquine in a target population of soldiers on military deployment and to verify the effect of covariates (e.g. body weight, age, sex, phospholipidosis, creatinine clearance, presence of malaria) on the PK characteristics of tafenoquine

Methods:

Study 044:

The study was a prospective study of tafenoquine in Thai soldiers on monthly prophylaxis deployed along the Thailand-Cambodian border.

Approximately 135 male Thai soldiers participated in the study and were treated with artesunate (300 mg on Day 1, 120 mg daily on Days 2 and 3) plus 200 mg daily doxycycline for 7 days to remove any pre-existing malarial infection. After pre-treatment, 104 soldiers received 400 mg tafenoquine (free base) daily for 3 days followed by 400 mg tafenoquine monthly for 5 consecutive months.

Study 033:

The study was a prospective, randomized, double-blind comparative study of tafenoquine and mefloquine in Australian soldiers on weekly malaria prophylaxis deployed on peacekeeping duties for 6 months in East Timor.

Approximately 490 subjects were given a loading dose of 200 mg tafenoquine (free base) per day for three consecutive days followed by an oral weekly maintenance dose of 200 mg tafenoquine for 6 months.

Population PK analyses was carried out using NONMEM Version 7.1.2, PDx-Pop Version 4.2 and Intel Visual Fortran Compiler Version 12 on a Microsoft Windows XP platform.

A one-compartment PK model with first order absorption and elimination was used to describe the PK of tafenoquine. For study 044, inter-individual and residual variability terms were included in the PK model. For study 033, inter-individual, residual variability and interoccasion variability on clearance terms were included in the PK model. As outlined in the publications, a covariate analysis was performed to identify potential covariates affecting the PK of tafenoquine and to evaluate the extent to which the covariates accounted for the

variability in the overall response. The validity of the final PK model was evaluated for study 033 using bootstrapping and the visual predictive check as outlined in the publication.

Results and Discussion:

Pharmacokinetic

Based upon the modeling results in the previous publications of study 044[1] and 033[2], a one-compartment pharmacokinetic model with first order absorption and elimination was used to describe the tafenoquine concentration versus time profiles. A schema of the one-compartment PK model is displayed in Figure 1-1.

Figure 1-1 One-Compartment Pharmacokinetic Model

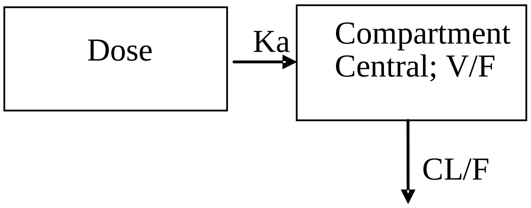

where

V/F = apparent volume of distribution for central compartment

Ka = first order absorption rate constant

CL/F = apparent clearance

Note: CL/F and V/F appear in NONMEM input file and control file as CL and V, respectively.

Table 1-1 presents the published and Table 1-2 presents the estimated Population PK parameters for study 044.

Table 1-1 Published Population PK Parameters of Final PK Model for Study 044

Parameters (Units)	Published Final Estimate (CV%)	Published Between-individual Variability[a]
CL (L/hr)	3.20 (2.7)	25.3 (14.0)
V (L)	1820 (1.7)	14.8 (21.1)
Ka (1/hr)	0.694 (12.8)	61.2 (167)
Residual Variability (CV%)	17.9 (8.6)	
Covariance between CL and V	0.0265 (21.5)	

[a] The magnitude of inter-individual variability is presented as CV%.
Note: Final estimate and inter-individual variability were from NONMEM estimates.
Source: Table 3[1].

Table 1-2 Estimated Population PK Parameters of Final PK Model for Study 044

Parameters (Units)	Final Estimate (CV%)	Between-individual Variability[a]
CL (L/hr)	3.18 (2.8)	25.4 (13.7)
V (L)	1820 (1.7)	15.0 (20.6)
Ka (1/hr)	0.700 (17.0)	61.1 (177)
Residual Variability (CV%)	17.9 (8.5)	
Covariance between CL/F and V/F	0.0273 (20.8)	

[a] The magnitude of inter-individual variability is presented as CV%.
Note: Final estimate and inter-individual variability were from NONMEM estimates.
Source: Attachment 2.12 (Tafenoquine044FOmodel0012.res).

Using the final model for study 044, the population estimates of CL, V, Ka, associated inter-individual variability, residual variability and covariance between CL and V of the final model were in agreement with the published estimates.

Table 1-3 and Table 1-4 present the published and estimated Population PK parameters for study 033, respectively.

Table 1-3 Published Population PK Parameters of Final PK Model for Study 033

Parameters (Units)	Published Final Estimate	Published Bootstrap 90% CI		Published Between-individual Variability[a]	Published Bootstrap 90% CI	
		Estimate	Lower - Upper		Estimate	Lower - Upper
CL (L/hr)	3.02	3.01	2.42 - 3.52	18	18	16 – 20
V (L)	1110	1110	874 - 1382	22	22	20 – 25
Ka (1/hr)	0.243	0.245	0.212 - 0.280	76	75	64 - 85
Weight centered on CL	0.448	0.447	0.249 - 0.816			
Weight centered on V	0.713	0.713	0.371 - 1.20			
IOV on CL (CV%)	18	18	16 - 20			
Proportional Residual Variability (CV%)	5.9	5.9	4.7 - 7.4			
Additive Residual Variability (ng/mL)	22.9	23.1	18.7 - 26.3			

[a] The magnitude of inter-individual variability is presented as CV%.
Note: Final estimate and inter-individual variability were from NONMEM estimates. IOV= interoccasion variability.
Source: Table 2[2].

Table 1-4 Estimated Population PK Parameters of Final PK Model for Study 033

Parameters (Units)	Final Estimate (CV%)	Bootstrap 90% CI		Between-individual Variability[a]	Bootstrap 90% CI	
		Estimate	Lower - Upper		Estimate	Lower - Upper
CL (L/hr)	3.02 (11.7)	3.02	2.28 – 3.61	18.1 (10.0)	10.1	6.5 – 12.6
V (L)	1110 (14.8)	1083	751 – 1440	22.4 (12.9)	22.3	18.8 – 25.3
Ka (1/hr)	0.244 (8.2)	0.242	0.200 – 0.278	75.7 (15.1)	74.8	58.1 – 85.8
Weight centered on CL	0.447 (38.0)	0.468	0.188 – 0.907			
Weight centered on V	0.721 (35.4)	0.799	0.330 – 1.470			
IOV on CL (CV%)	18.3 (12.2)	18.1	16.2 – 19.7			
Proportional Residual Variability (CV%)	5.96 (29.6)	6.0	3.9 – 7.5			
Additive Residual Variability (ng/mL)	23.02 (19.4)	23.02	18.1 – 27.1			

[a] The magnitude of inter-individual variability is presented as CV%.
Note: Final estimate and inter-individual variability were from NONMEM estimates. IOV= interoccasion variability.
Source: Attachment 3.9 (Tafenoquine033FOCEImodel0009.res)

Similar to the results for study 044, the Population PK parameter estimates for study 033 are in agreement with the published results.

Conclusions:

- For both studies 044 and 033, Population PK parameters of tafenoquine and associated variability using the final model are in good agreement with the published results.

2. INTRODUCTION

Tafenoquine is an 8-aminoquinoline antimalarial agent active against all stages of the malarial parasite. Given the broad range of efficacy during the lifecycle of the parasite, tafenoquine has potential therapeutic uses as a malarial prophylactic and in relapse prevention.

This population pharmacokinetic (PK) analysis was performed using data obtained from a Phase-II clinical study of tafenoquine in Thai soldiers deployed on security operations on the Thailand-Cambodian border (study 044)[1] and in a Phase-III clinical study of tafenoquine in Australian soldiers deployed for 6 months in areas endemic with malaria (study 033)[2].

3. OBJECTIVES

The objectives of this analysis were:

- to validate the NONMEM input files used in the prior analyses,
- to verify a population PK model of tafenoquine in a target population of soldiers on military deployment and to verify the effect of covariates (e.g. body weight, age, sex, phospholipidosis, creatinine clearance, presence of malaria) on the PK characteristics of tafenoquine

Primary Endpoints

- Endpoints for population PK were the population estimates of PK parameters (e.g., CL, V, Ka), interoccasion variability (IOV, study 033 only), associated inter-subject variability and residual error.

- Endpoints for the covariate analysis were the confirmation of significant covariates that are retained in the PK model and quantification of their effects.

4. METHODS

4.1. Study Design

Study 044:

The study was a prospective study of tafenoquine in Thai soldiers on monthly prophylaxis deployed along the Thailand-Cambodian border.

Approximately 135 male Thai soldiers participated in the study and were treated with artesunate (300 mg on Day 1, 120 mg daily on Days 2 and 3) plus 200 mg daily doxycycline for 7 days to remove any pre-existing malarial infection. After pre-treatment, 104 soldiers received 400 mg tafenoquine (free base) daily for 3 days followed by 400 mg tafenoquine monthly for 5 consecutive months.

Study 033:

The study was a prospective, randomized, double-blind comparative study of tafenoquine and mefloquine in Australian soldiers on weekly malaria prophylaxis deployed on peacekeeping duties for 6 months in East Timor.

Approximately 490 subjects were given a loading dose of 200 mg tafenoquine (free base) per day for three consecutive days followed by an oral weekly maintenance dose of 200 mg tafenoquine for 6 months.

4.1.1. Pharmacokinetic Collection

Study 044:

Blood samples for the determination of tafenoquine concentration in plasma for PK evaluation were taken randomly in the field after beginning the loading dose at approximately 8, 24, 48, 56 hours and then at every 3 to 4 day intervals up until the first monthly dose. After each monthly dose, samples were obtained at approximately 8 hours post-dose, mid-month, and at the end of the dosing interval (trough concentration). Following the last monthly dose, samples were obtained at 4, 8, 12, and 24 hours post dose and at every 3 to 4 day intervals for 2 months.

Study 033:

Blood samples for the determination of tafenoquine concentration in plasma for PK evaluation were taken at prerandomized times after the last loading dose and then at prerandomized times at weeks 4, 8 and 16. Samples were collected on predetermined days after dosing on each of the assessment weeks. The predetermined days included Day 1 (early postdose, absorption phase), Days 3 and 5 (72 hours to 120 hours postdose), and Day 7 (predose, trough phase). The date and exact time of blood sampling were recorded.

4.2. Data Assembly

4.2.1. Description of Input Data

All data used in the population PK analysis were obtained from data used in previously published tafenoquine modeling efforts[1,2]. Approximately 135 male Thai soldiers participated in study 044 and approximately 490 subjects participated in study 033.

4.2.2. Handling of Input Data

The data were prepared for analysis and validated using SAS Version 9.1.3 (SAS Institute Inc., Cary, NC). Actual dosing and actual sampling times were used for the analysis.

4.2.3. Bioanalytical Methodology

For study 044, plasma samples were analyzed for tafenoquine concentration by HPLC with fluorescence detection. For study 033, plasma samples were analyzed for tafenoquine concentrations using a validated LC/MS/MS method.

4.3. Data Analysis

Population PK analysis was carried out using NONMEM Version 7.1.2, PDx-Pop Version 4.2 and Intel Visual Fortran Compiler Version 12 on a Microsoft Windows XP platform.

Study 044:

The tafenoquine data from a prior Phase II[1] (study 044) was used to establish a structural PK model and explore covariates. Table 4-1 outlines model development:

Table 4-1 Model Development for Study 044[1]

Number/Model		OFV^a
Forwards stepwise addition		
1	$CL/F=\theta_1$; $V/F=\theta_2$; $K_a=\theta_3$	
2	$CL/F=\theta_1 \cdot (WT\text{-}60.3)+\theta_2$; $V/F=\theta_3$; $K_a=\theta_4$	-1^b
3	$CL/F=\theta_1 \cdot (AGE\text{-}28.9)+\theta_2$; $V/F=\theta_3$; $K_a=\theta_4$	-1^b
4	$CL/F=\theta_1$; $V/F=\theta_2 \cdot (WT\text{-}60.3)+\theta_3$; $K_a=\theta_4$	$-12^{b,\star}$
5	$CL/F=\theta_1$; $V/F=\theta_2 \cdot (AGE\text{-}28.9)+\theta_3$; $K_a=\theta_4$	$-8^{b,\star}$
6	$CL/F=\theta_1 \cdot MAL+\theta_2 \cdot (1\text{-}MAL)$; $V/F=\theta_3$; $K_a=\theta_4$	-58^b
7	$CL/F=\theta_1$; $V/F=\theta_2 \cdot (WT\text{-}60.3)+\theta_3 \cdot (AGE\text{-}28.9)+\theta_4$; $K_a=\theta_5$	$-18^{b,\star}$
8	$CL/F=\theta_1 \cdot MAL+\theta_2 \cdot (1\text{-}MAL)$; $V/F=\theta_3 \cdot (WT\text{-}60.3)+\theta_4 \cdot (AGE\text{-}28.9)+\theta_5$; $K_a=\theta_6$	-83^b
Backwards stepwise elimination		
9	$CL/F=\theta_1$; $V/F=\theta_2+\theta_3 \cdot (WT\text{-}60.3)+\theta_4 \cdot (AGE\text{-}28.9)$; $K_a=\theta_5$	$+53^c$
10	$CL/F=\theta_1 \cdot MAL+\theta_2 \cdot (1\text{-}MAL)$; $V/F=\theta_3+\theta_4 \cdot (AGE\text{-}28.9)$; $K_a=\theta_5$	$+11^c$
11	$CL/F=\theta_1 \cdot MAL+\theta_2 \cdot (1\text{-}MAL)$; $V/F=\theta_3+\theta_4 \cdot (WT\text{-}60.3)$; $K_a=\theta_5$	$+2^c$

[a]Objective function value, [b]Change in OFV from model no. 1 (OFV = −4556), [c]Change in OFV from model no. 8 (OFV = −4627).
CL/*F*: clearance ($l\ h^{-1}$), *V/F*: volume of distribution (l), K_a: absorption rate constant (h^{-1}), (WT-60.3): centred body weight (kg), (AGE-28.9): centred age (years), MAL: presence (1) absence (0) of malaria. ⋆$P<0.01$ (full model *vs* reduced model).

Source: Table 2[1].

An additional model (Model 12) was explored using model 1 and estimating the off diagonal elements of the Covariance Matrix (Omega matrix) using the $OMEGA BLOCK (2) function as mentioned in the text[1].

Study 033:

The tafenoquine data from a prior Phase III[2] (study 033) was used to establish a structural PK model and explore covariates. Table 4-2 outlines model development:

Table 4-2 Model Development for Study 033[2]

Model	Parameterization[d]	ΔOFV[a]
1	$CL/F = \theta_1;\ V/F = \theta_2;\ K_a = \theta_3$	
2	$CL/F = \theta_1 \cdot (1 + \theta_4 \cdot age/25.4);\ V/F = \theta_2;\ K_a = \theta_3$	−2
3	$CL/F = \theta_1;\ V/F = \theta_2 \cdot (1 + \theta_4 \cdot age/25.4);\ K_a = \theta_3$	−9[b]
4	$CL/F = \theta_1 \cdot (1 + \theta_4 \cdot CL_{CR}/121);\ V/F = \theta_2;\ K_a = \theta_3$	−4
5	$CL/F = \theta_1 \cdot PHOS + \theta_4 \cdot (1 - PHOS);\ V/F = \theta_2;\ K_a = \theta_3$	0
6	$CL/F = \theta_1;\ V/F = \theta_2 \cdot PHOS + \theta_4 \cdot (1 - PHOS);\ K_a = \theta_3$	−1[b]
7	$CL/F = \theta_1 \cdot sex + \theta_4 \cdot (1 - sex);\ V/F = \theta_2;\ K_a = \theta_3$	−3[b]
8	$CL/F = \theta_1;\ V/F = \theta_2 \cdot sex + \theta_4 \cdot (1 - sex);\ K_a = \theta_3$	−12
9[c]	$CL/F = \theta_1 \cdot (1 + \theta_4 \cdot WT/80.9);\ V/F = \theta_2 \cdot (1 + \theta_5 \cdot WT/80.9);\ K_a = \theta_3$	−39
10	$CL/F = \theta_1 \cdot (WT/70)^{0.75};\ V/F = \theta_2 \cdot (WT/70)^{1.0};\ K_a = \theta_3$	+37[b]

[a] ΔOFV, change in OFV from that of model 1 (OFV = 22,177).
[b] Rounding errors occurred during fitting.
[c] Final model.
[d] WT/80.9, body weight (kg) centered on average weight (80.9 kg); age/25.4, age (years) centered on average age (25.4 years); $CL_{CR}/121$, CL_{CR} (ml/min) centered on average CL_{CR} (121 ml/min); PHOS, phospholipidosis (tested in 77 subjects; 1 = phospholipidosis present, 0 = phospholipidosis not present); sex, male = 0 and female = 1.

Source: Table 1[2].

4.4. Modeling Assumptions

4.4.1. Description of PK Model

Based upon the modeling results in the previous publications of study 044[1] and 033[2], a one-compartment pharmacokinetic model with first order absorption and elimination was used to describe the tafenoquine concentration versus time profiles. A schema of the one-compartment PK model is displayed in Figure 4-1.

Figure 4-1 One-Compartment Pharmacokinetic Model

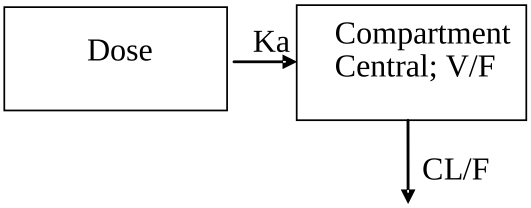

where

V/F	=	apparent volume of distribution for central compartment
Ka	=	first order absorption rate constant
CL/F	=	apparent clearance

Note: CL/F and V/F appear in NONMEM input file and control file as CL and V, respectively.

This one-compartment PK model was specified in the NONMEM control file and was parameterized in terms of CL, V, and Ka, using the PREDPP ADVAN2 with the TRANS2 subroutine. First order estimation was used for study 044 and first order conditional estimation (FOCE) with interaction was used as the estimation method for study 033.

4.4.2. Variability Models

4.4.2.1. Model for inter-individual variability

Pharmacokinetic

Between-individual variability (ETA or η), which is the difference between the individual parameter estimate and the population mean estimate of final PK parameters, was described by an exponential error model as described here for both studies 044 and 033:

$$Pi = \hat{P} \exp(\eta_i)$$

where:

Pi is the estimated parameter for the ith individual.

$\hat{P}$ is the population value for the parameter.

η_i are individual inter-individual random effects for the ith patient and parameter P and are distributed as follows: $\eta \sim N(0, \omega^2)$, and ω^2 is the variance of the inter-individual random effect.

4.4.2.2. Model for residual variability

Pharmacokinetic

Residual variability (EPSILON or ε), which is the difference between the observed and the individual predicted observations of the final PK model, was described as an exponential error model for study 044 as shown below:

$$C_{ij} = \hat{C}_{ij} * \exp(\varepsilon_{pij})$$

Residual variability was described by a proportional and additive error model for study 033 as shown below:

$$C_{ij} = \hat{C}_{ij} (1 + \varepsilon_{1,pij}) + \varepsilon_{2,pij}$$

Where:

C_{ij} is the jth observation in the ith individual.

$\hat{C}_{ij}$ is the jth predicted value in the ith individual.

ε_{pij} is the proportional residual random error for the ith individual and the jth measurement and is distributed as follows: $\varepsilon \sim N(0,\sigma^2)$.

4.4.2.3. Model for interoccasion variability

Pharmacokinetic

Interoccasion variability (IOV, kappa or κ), is a random variable representing the variability of a PK parameter across different occasions where each occasion is defined as a dose (or several sequential doses) followed by at least one observation. IOV was assumed to be normally distributed having a mean of 0 and a variance of π^2 and that the variance of each parameter was from the same distribution. IOV was described on CL/F for study 033 as shown below:

$$CL_{ij} = \hat{CL} * \exp(\eta_i + \kappa_j)$$

where:

CL_{ij} is the estimated parameter for the ith individual on the jth occasion.

CL is the population value for the parameter.

η_i is the individual inter-individual random effects for the ith patient and are distributed as follows: $\eta \sim N(0,\omega^2)$, and ω^2 is the variance of the inter-individual random effect.

κ_j is the IOV on the jth occasion.

4.4.3. Covariate Analysis

Covariate analysis was performed to the base PK model to identify potential covariates and to evaluate the extent to which the covariates accounted for the variability in the PK parameters as outlined in each publication[1,2] and presented in Table 4-1 and Table 4-2.

The following variables were selected in the publication for evaluation as potential covariates of CL/F and V/F for study 044: weight (WT), age, presence or absence of malaria (MAL) and the following were evaluated for study 033: age, creatinine clearance (CLCR), presence or absence of malaria (HIST), sex, and weight (WT). It must be noted that in the publication for study 033, Phospholipidosis (PHOS) was considered as a covariate, however, a corresponding binary variable was not identified in the clinical data nor in the provided NONMEM dataset. The binary variable HIST, which was supplied in the NONMEM dataset was substituted in its place.

4.5. Model Evaluation

As reported in the reference paper, the final PK model for study 033[1] was evaluated using bootstrapping and a posterior predictive check. The final PK model for study 044[2] was evaluated by plotting observed tafenoquine concentrations versus model predicted concentrations, weighted residuals versus predicted concentrations, and weighted residuals versus subject ID as reported in the publication.

4.5.1. Bootstrapping

For study 033, bootstrapping was reported in the publication[1]. As a result, bootstrapping was performed to evaluate the final PK model. Bootstrapping is data re-sampling method for estimating sampling variances, confidence intervals, and stability of regression models. Using the bootstrap approach, the bootstrap parameter values are obtained by repeatedly fitting the final population model to a reasonable number of bootstrap samples. The mean and confidence interval (CI) values of the bootstrap parameters are then compared to the final population model parameter estimates and associated CIs from NONMEM.

Bootstrap analysis procedure consisted of the following steps:

1. The replication of the data file is obtained by random draw (with replacement) from the original data file.

2. The final model is fitted to the resulting data file, and the model parameter estimates are saved for the final analysis.

3. Steps 1 and 2 are repeated 100-1000 times (with different random draws)

4. Distribution of the parameter estimates are plotted and visually analyzed.

5. Bootstrap parameter estimates, standard errors and CIs are obtained.

The PDx-Pop Model Evaluation module automated the bootstrap analysis, computation of bootstrap estimates, variance, and CIs and plotting of histogram of the parameter estimates. The 95% bootstrap CIs were determined for the PK parameters derived from 200 bootstrap datasets and compared to the original parameters obtained from the final model (PDx-Pop manual).

4.5.2. Posterior predictive check

For study 033, a posterior predictive check was reported in publication[1]. As a result, a posterior predictive check was performed by simulating the tafenoquine concentrations from the original datasets using the parameter estimates obtained in the final PK modeling step. One thousand (1000) predicted profiles were simulated for each original subjects/patients. Random effects were included in the simulation. The median, 5th percentile, and 95th percentile PK concentrations versus time profiles from the simulations were compared with observed tafenoquine concentrations. Simulated results (5th percentile, median, and 95th

percentile) were visually compared with the observed data to evaluate how well the models predicted the data.

5. RESULTS

5.1. Study 044

Based upon the modeling results in the previous publications of study 044[1] and 033[2], a one-compartment pharmacokinetic model with first order absorption and elimination was used to describe the tafenoquine concentration versus time profiles. A schema of the one-compartment PK model is displayed in Figure 4-1.

Table 5-1 presents the published and modeled development of the structural and covariate model for study 044.

Table 5-1 Published and Modeled Development of Structural and Covariate Population PK Model for Study 044

Model	Parameterization	Published OFV	Published ΔOFV	Modeled OFV	Modeled ΔOFV
Forward Stepwise Addition					
1	No Covariates	-4556	.	-4593.61	.
2	Centered WT on CL		-1	-4593.61	0.00
3	Centered Age on CL		-1	-4593.61	0.00
4	Centered WT on V		-12	-4603.04	-9.44
5	Centered Age on V		-8	-4600.50	-6.89
6	Malaria Infection on CL		-58	-4650.79	-57.18
7	Centered WT and Age on V		-18	-4609.27	-15.67
8	Malaria Infection on CL; Centered WT and Age on V	-4627	-83	-4661.81	-68.20
Backwards Stepwise Elimination					
9	Centered WT and Age on V		+53*	-4609.27	52.54*
10	Malaria Infection on CL; Centered Age on V		+11*	-4652.64	9.17*
11	Malaria Infection on CL; Centered WT on V		+2*	-4660.31	1.50*
**12****	**No Covariates; Covariance CL,V**	**-4601**	**-45**	**-4640.83**	**-47.2**

OFV= objective function value; NR= not reported.
* Difference in OFV from Model 8; **Final Model, reported in publication text [1].

Table 5-2 presents the published and Table 5-3 presents the estimated Population PK parameters for study 044.

Table 5-2 Published Population PK Parameters of Final PK Model for Study 044

Parameters (Units)	Published Final Estimate (CV%)	Published Between-individual Variability[a]
CL (L/hr)	3.20 (2.7)	25.3 (14.0)
V (L)	1820 (1.7)	14.8 (21.1)
Ka (1/hr)	0.694 (12.8)	61.2 (167)
Residual	17.9 (8.6)	

Variability (CV%)		
Covariance between CL and V	0.0265 (21.5)	

[a] The magnitude of inter-individual variability is presented as CV%.
Note: Final estimate and inter-individual variability were from NONMEM estimates.
Source: Table 3[1].

Table 5-3 Estimated Population PK Parameters of Final PK Model for Study 044

Parameters (Units)	Final Estimate (CV%)	Between-individual Variability[a]
CL (L/hr)	3.18 (2.8)	25.4 (13.7)
V (L)	1820 (1.7)	15.0 (20.6)
Ka (1/hr)	0.700 (17.0)	61.1 (177)
Residual Variability (CV%)	17.9 (8.5)	
Covariance between CL/F and V/F	0.0273 (20.8)	

[a] The magnitude of inter-individual variability is presented as CV%.
Note: Final estimate and inter-individual variability were from NONMEM estimates.
Source: Attachment 2.12 (Tafenoquine044FOmodel0012.res).

For study 044, the population estimates of CL, V, Ka, associated inter-individual variability, residual variability and covariance between CL/F and V/F of the final model were in agreement with the published estimates.

The population predicted (PRED) plasma concentrations of tafenoquine for study 044 were obtained using model-estimated population PK parameters from the final PK model, and the published and newly modeled plots of the predicted plasma concentrations versus observed plasma concentrations of tafenoquine were presented in Figure 5-1. The weighted residuals (WRES) versus model predicted (PRED) plasma concentrations of tafenoquine were presented in Figure 5-2. The weighted residuals (WRES) versus subject number were presented in Figure 5-3.

Figure 5-1 Published (A) and Modeled (B) Predicted (PRED) Concentration vs. Observed (DV) Tafenoquine Concentrations for Study 044 (Linear Scale)

A. Published

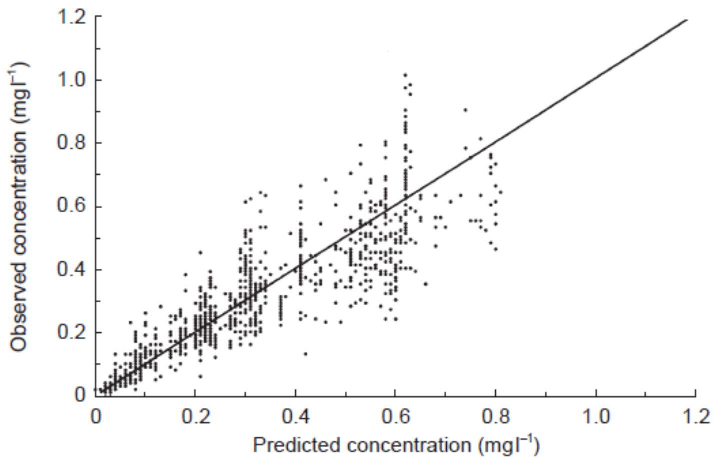

Source: Figure 3a[1]

B. Modeled

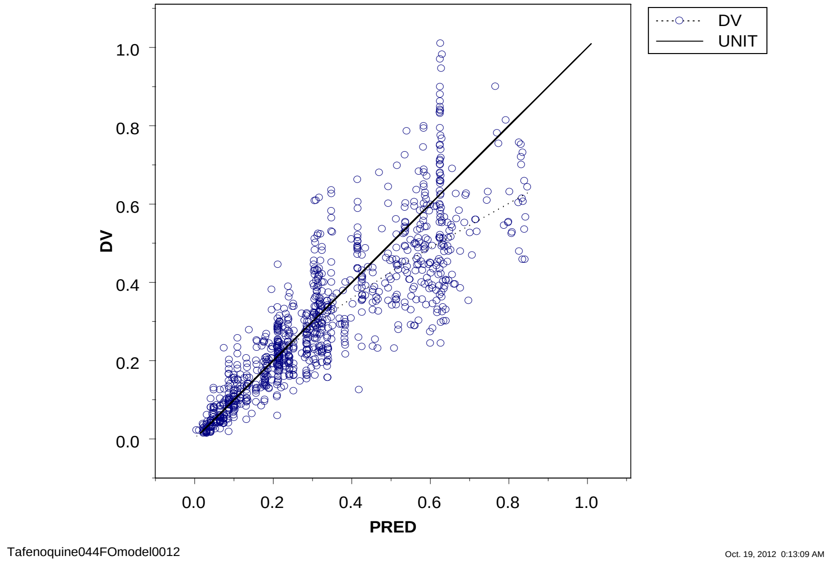

Figure 5-2 Published (A) and Modeled (B) Weighted Residuals (WRES) vs. Predicted (PRED) Tafenoquine Concentrations for Study 044 (Linear Scale)

A. Published

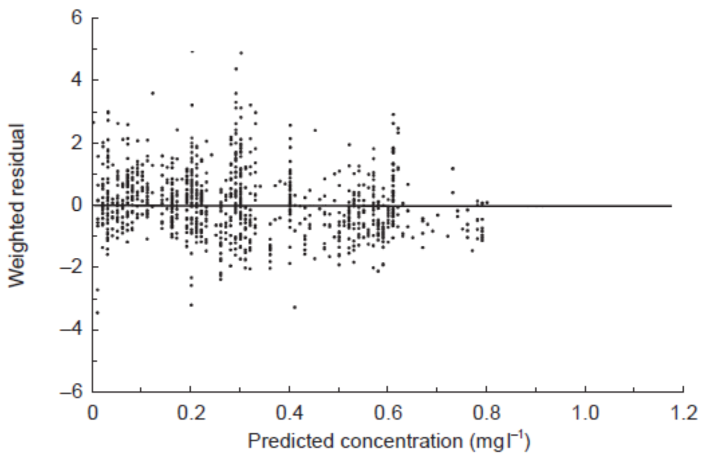

Source: Figure 3b[1].

B. Modeled

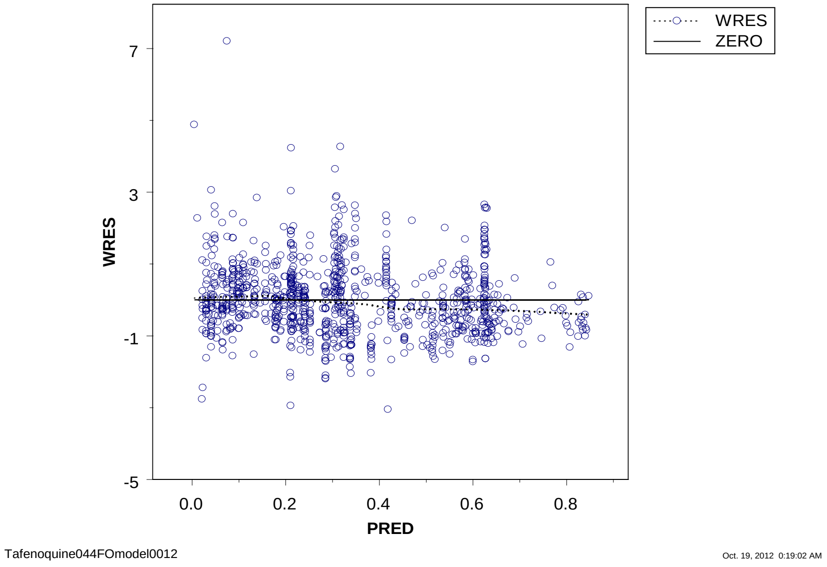

Figure 5-3 Published (A) and Modeled (B) Weighted Residuals (WRES) vs. Subject number for Study 044 (Linear Scale)

A. Published

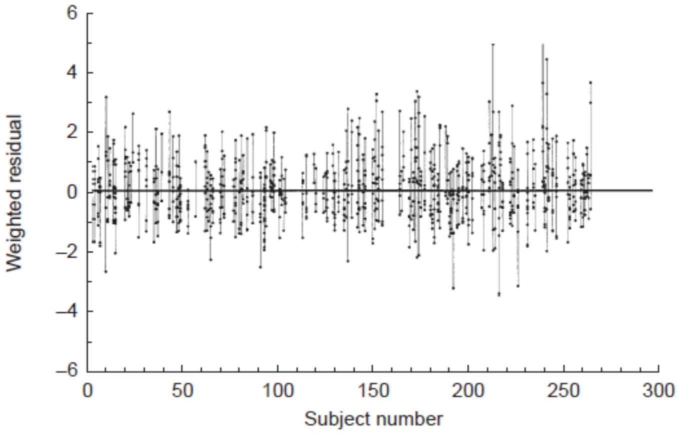

Source: Figure 3c[1].

B. Modeled

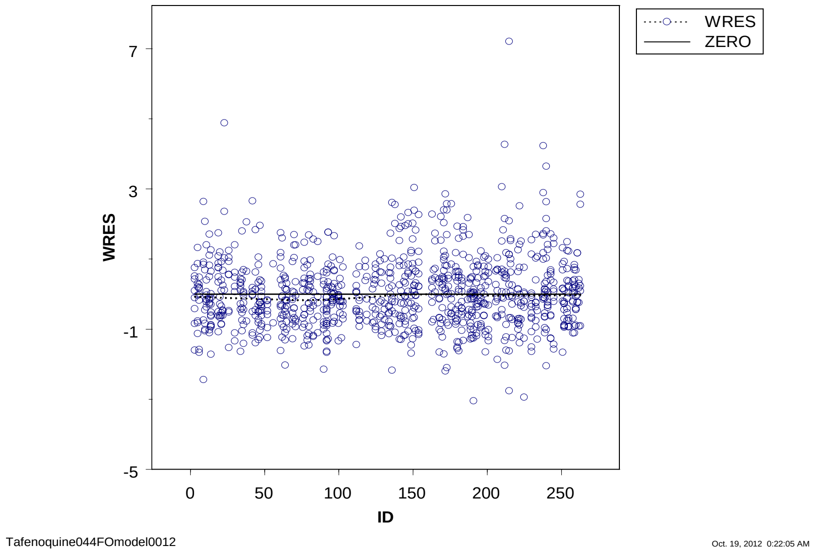

5.2. Study 033

Table 5-4 presents the published and modeled development of the structural and covariate model for study 033.

Table 5-4 Published and Modeled Development of Structural and Covariate Population PK Model for Study 033

Model	**Parameterization**	**Published OFV**	**Pulbished ΔOFV**	**Modeled OFV**	**Modeled ΔOFV**
1	IOV on CL;<br>Covariance between CL, and V	22177	.	22179.08	.
2	IOV on CL;<br>Covariance between CL and V; AGE on CL		-2	22177.43	-1.7
3	IOV on CL;<br>Covariance betweem CL and V;<br>AGE on V		-9	22170.59	-8.5
4	IOV on CL;<br>Covariance between CL and V;<br>CLCR on CL		-4	22174.90	-4.2
5	IOV on CL;<br>Covariance between CL and V;<br>PHOS(HIST) on CL		0	22178.99	-0.1
6	IOV on CL;<br>Covariance between CL and V;<br>PHOS(HIST) on V		-1	22178.03	-1.1
7	IOV on CL;<br>Covariance between CL and V;<br>SEX on CL		-3	22176.44	-2.6
8	IOV on CL;<br>Covariance between CL and V;<br>SEX on V		-12	22167.33	-11.8
**9***	**IOV on CL;<br>Covariance between CL and V;<br>Centered WT on CL and V**		**-39**	**22139.85**	**-39.2**
10	IOV on CL;<br>Covariance between CL and V;<br>Allometric WT on CL and V		+37	22215.61	36.5

OFV= objective function value; IOV= interoccasion variability.
*Final Model [2].

Table 5-5 and Table 5-6 present the published and estimated Population PK parameters for study 033, respectively.

Table 5-5 Published Population PK Parameters of Final PK Model for Study 033

Parameters (Units)	Published Final Estimate	Published Bootstrap 90% CI		Published Between-individual Variability[a]	Published Bootstrap 90% CI	
		Estimate	Lower - Upper		Estimate	Lower - Upper
CL (L/hr)	3.02	3.01	2.42 - 3.52	18	18	16 – 20
V (L)	1110	1110	874 - 1382	22	22	20 – 25
Ka (1/hr)	0.243	0.245	0.212 - 0.280	76	75	64 - 85
Weight centered on CL	0.448	0.447	0.249 - 0.816			
Weight centered on V	0.713	0.713	0.371 - 1.20			
IOV on CL (CV%)	18	18	16 - 20			
Proportional Residual Variability (CV%)	5.9	5.9	4.7 - 7.4			
Additive Residual Variability (ng/mL)	22.9	23.1	18.7 - 26.3			

[a] The magnitude of inter-individual variability is presented as CV%.
Note: Final estimate and inter-individual variability were from NONMEM estimates. IOV= interoccasion variability.
Source: Table 2[2].

Table 5-6 Estimated Population PK Parameters of Final PK Model for Study 033

Parameters (Units)	Final Estimate (CV%)	Bootstrap 90% CI		Between-individual Variability[a]	Bootstrap 90% CI	
		Estimate	Lower - Upper		Estimate	Lower - Upper
CL (L/hr)	3.02 (11.7)	3.02	2.28 – 3.61	18.1 (10.0)	10.1	6.5 – 12.6
V (L)	1110 (14.8)	1083	751 – 1440	22.4 (12.9)	22.3	18.8 – 25.3
Ka (1/hr)	0.244 (8.2)	0.242	0.200 – 0.278	75.7 (15.1)	74.8	58.1 – 85.8
Weight centered on CL	0.447 (38.0)	0.468	0.188 – 0.907			
Weight centered on V	0.721 (35.4)	0.799	0.330 – 1.470			
IOV on CL (CV%)	18.3 (12.2)	18.1	16.2 – 19.7			
Proportional Residual Variability (CV%)	5.96 (29.6)	6.0	3.9 – 7.5			
Additive Residual Variability (ng/mL)	23.02 (19.4)	23.02	18.1 – 27.1			

[a] The magnitude of inter-individual variability is presented as CV%.
Note: Final estimate and inter-individual variability were from NONMEM estimates. IOV= interoccasion variability.
Source: Attachment 3.9 (Tafenoquine033FOCEImodel0009.res)

The bootstrapping technique was used to evaluate the final PK models. Two hundred (200) bootstrap samples for PK were employed to evaluate the final models. The bootstrap estimates were derived for the PK parameters and compared to the original parameters obtained for the final model.

Similar to the results for study 044, the Population PK parameter estimates for study 033 are in agreement with published results.

Figure 5-4 presents the results of the published and modeled posterior predictive check of the Final Population model for study 033.

Figure 5-4 Published (A) and Modeled (B) predicted vs. Observed Concentrations of Tafenoquine (Linear Scale)

A. Published

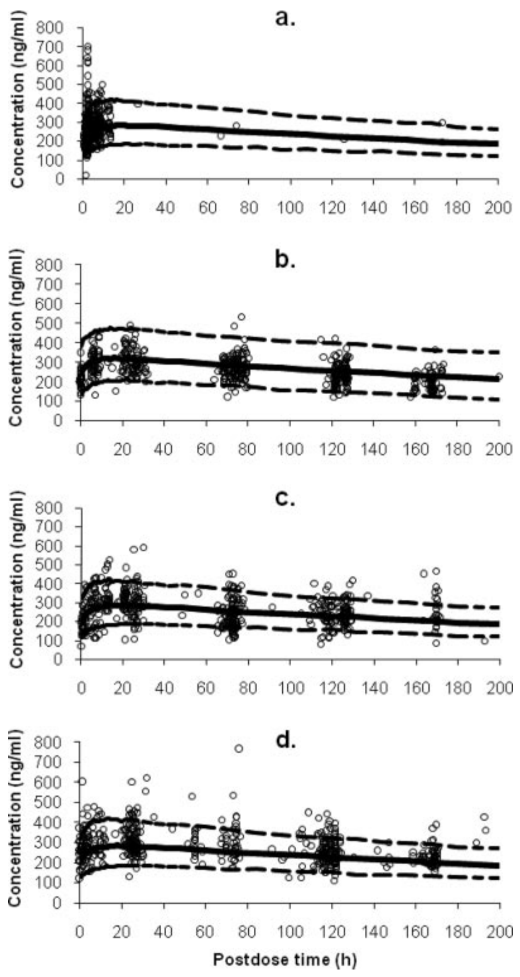

B. Modeled

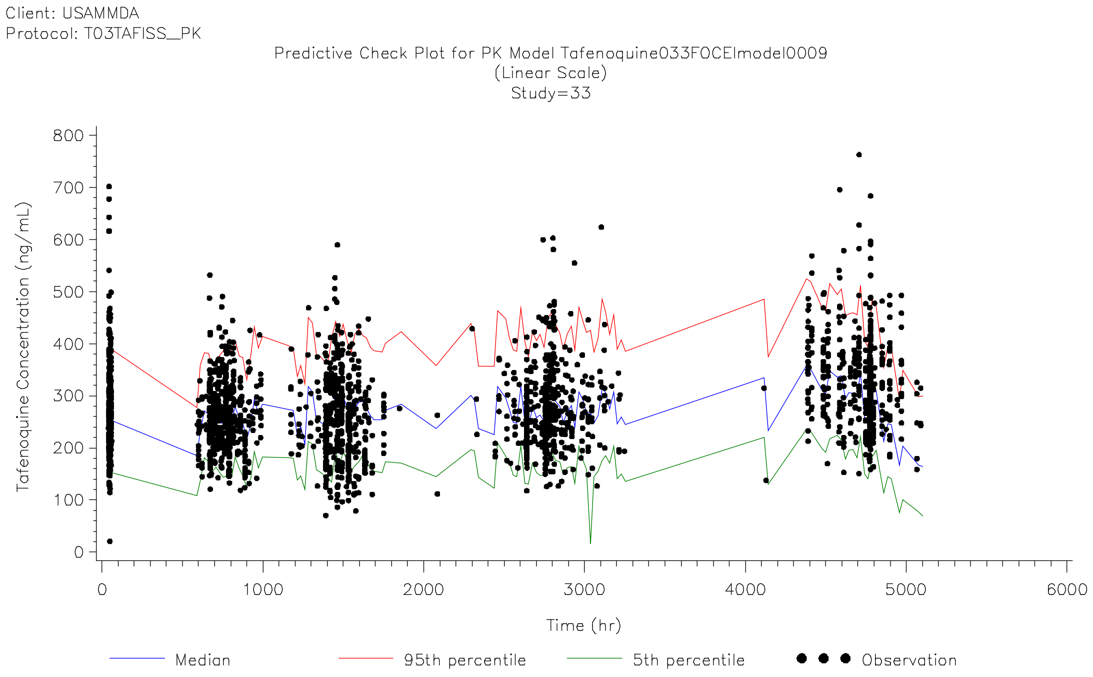

Simulations were performed using NONMEM parameter estimates from the final PK model and were carried out for the study 033 dataset. One thousand (1000) simulations for each original subject were carried out. The NONMEM control file used for the simulation of tafenoquine is presented in Attachment 3.11. Median, 5th percentile, and 95th percentile plot of model-predicted vs. observed concentrations is presented in Figure 5-4. Similar to the published predictive check, a majority of the tafenoquine plasma concentrations lie within the 90% CI (5th to 95th percentile).

6. DISCUSSION AND CONCLUSIONS

6.1. Discussion

Confirmatory Population PK modeling was performed on plasma tafenoquine concentrations using data published previously[1,2]. The modeling effort outlined in this report followed the modeling effort as published and presented in Table 4.1 (for study 044) and 4.2 (for study 033).

Results showed that a one-compartment PK model with first order absorption and elimination was an appropriate base PK model for describing PK of tafenoquine following oral administration.

In general, there was a good agreement between the published and modelled PK parameter and variability estimates.

Mean bootstrap estimates of all PK parameters for study 033 were compared to those from the final PK model. The bootstrap estimates agree with the final estimated PK parameter estimates as well as to the published estimates.

Simulations were performed using the NONMEM parameter estimates from the final PK model for study 033 and were carried out for actual sampling times. The results are in agreement with the published results as a majority of the tafenoquine concentrations were inside the 90% confidence interval (the 5th and 95th percentile lines).

6.2. Conclusions

- For both studies 044 and 033, Population PK parameters of tafenoquine and associated variability using the final model are in good agreement with the published results.

7. REFERENCES

1. Edstein, MD, Kocisko, DA, Brewer, TG, Walsh, DS, Eamsila, C and Charles, BG (2001). Population pharmacokinetics of the new antimalarial agent tafenoquine in Thai soldiers. British Journal of Clinical Pharmacology, 52, 663-670.

2. Charles, BG, Miller, AK, Nasveld, PE, Reid, MG, Harris, IE, Edstein, MD (2007). Population pharmacokinetics of tafenoquine during malaria prophylaxis in healthy subjects. Antimicrobial Agents and Chemotherapy 51, 2709-2715.

Part II. POOLED DATA POPULATION PK MODELING

8. SUMMARY

Objectives:

The objectives of this analysis were:

- to develop population pharmacokinetic (PK) model of tafenoquine using pooled PK data from 10 clinical studies and to assess the effect of covariates such as body weight, age, sex, race, and food on the PK characteristics of tafenoquine

Methods:

This population PK analysis was performed using data obtained from 10 Phase I/II clinical studies of tafenoquine (Army Study No's: 1, 2, 3, 4, 5, 14, 15, 33, 44, and 58). Data for this analysis was provided as validated NONMEM ready datasets for Army Study No's: 1, 2, 3, 4, 5, 14, 15, and 58. However, for Army Study No's: 33 and 44, unvalidated datasets were provided which were validated first using the raw datasets. Then, a confirmatory PK modeling was performed (Part 1 of this report) before incorporating the data into the pooled dataset.

Population PK and PKPD analyses were carried out using NONMEM Version 7.1.2, PDx-Pop Version 4.2 and Intel Visual Fortran Compiler Version 12 on a Microsoft Windows XP platform.

A one-compartment PK model with first order absorption and elimination was selected to describe the PK of tafenoquine. Between-individual and residual variability terms were included in the PK model. In addition, a covariate analysis was performed to identify potential covariates affecting the PK of tafenoquine and to evaluate the extent to which the covariates accounted for the variability in the overall response. The validity of the final PK model was evaluated using bootstrapping and the posterior predictive check.

Results and Discussion:

Pharmacokinetic

From previous modeling experience (Part 1 of this report), tafenoquine followed a mono-exponential kinetics. Therefore, a one-compartment PK model with first order absorption and elimination was used as a starting point for the base PK model in the population PK analysis.

A schematic representation of the final PK model is presented in Figure 4-1. The population PK parameters of tafenoquine from the final PK model and the results from the final model evaluation by bootstrapping are presented in Table 8-1.

Table 8-1 Population PK Parameters of Final PK Model

Parameters (Units)	Final Estimate	Bootstrap 95% CI		Between-individual Variability [a]
		Lower	Upper	
CL/F (L/hr) = $\theta_{CL} \times (WT/75)^{\theta_{CL\text{-}WT}} \times (AGE/25)^{\theta_{CL\text{-}AGE}}$				23.6%
θ_{CL}	4.17	4.080	4.230	
$\theta_{CL\text{-}WT}$	0.552	0.474	0.637	
$\theta_{CL\text{-}AGE}$	-0.200	-0.267	-0.138	
V/F (L) = $\theta_{V} \times (WT/75)^{\theta_{V\text{-}WT}} \times (AGE/25 \times (\theta_{V\text{-}FOOD})^{FOOD}$				24.1%
θ_{V}	2470	2340	2630	
$\theta_{V\text{-}WT}$	0.781	0.652	0.901	
$\theta_{V\text{-}FOOD}$	0.822	0.761	0.861	
Ka (1/hr)	0.359	0.321	0.384	54.1%

a. The magnitude of inter-individual variability was presented as CV%.
Note: Final estimate and inter-individual variability were from NONMEM estimates.
Source: Attachment 8.2 (USARMYTAFPKC0409.sum)

For oral tafenoquine, the population CL/F and V/F are determined to be 4.17 L/hr and 2470 L, respectively. The first order absorption rate constant of oral tafenoquine was 0.359 1/hr. The inter-individual variability of CL/F, V/F, and Ka was 23.6%, 24.1% and 54.1%, respectively.

The final PK model revealed that apparent clearance (CL/F) of tafenoquine is a function of body weight (WT) and age. Tafenoquine CL/F was found to increase with increasing body weight and decreased with age. The final PK model revealed that apparent volume of distribution (V/F) of tafenoquine is a function of body weight (WT) and food. Tafenoquine V/F was found to increase with increasing body weight and decreased with food.

The individual predicted (IPRED) plasma concentrations of tafenoquine were obtained using the individual post hoc PK parameters from the final PK model, and the plots of the individual predicted plasma concentrations versus observed plasma concentrations of tafenoquine were presented in Figure 8-1. The population predicted (PRED) plasma

concentrations of tafenoquine were obtained using the model-estimated population PK parameters from the final PK model, and the plots of the population predicted plasma concentrations versus observed plasma concentrations of tafenoquine were presented in Figure 8-2. The IPRED versus the observed concentration plot showed good prediction of the observed concentrations (Figure 8-2).

Using the final population PK model, tafenoquine plasma concentrations were simulated for a posterior predictive check. The model-predicted versus observed concentrations of tafenoquine are displayed in Figure 8-3. The posterior predictive check demonstrated the adequacy of the final PK model to reproduce tafenoquine concentrations in all 10 studies combined.

Figure 8-1 Individual Predicted (IPRED) vs. Observed (DV) Tafenoquine Concentrations (Linear and Logarithmic Scales)

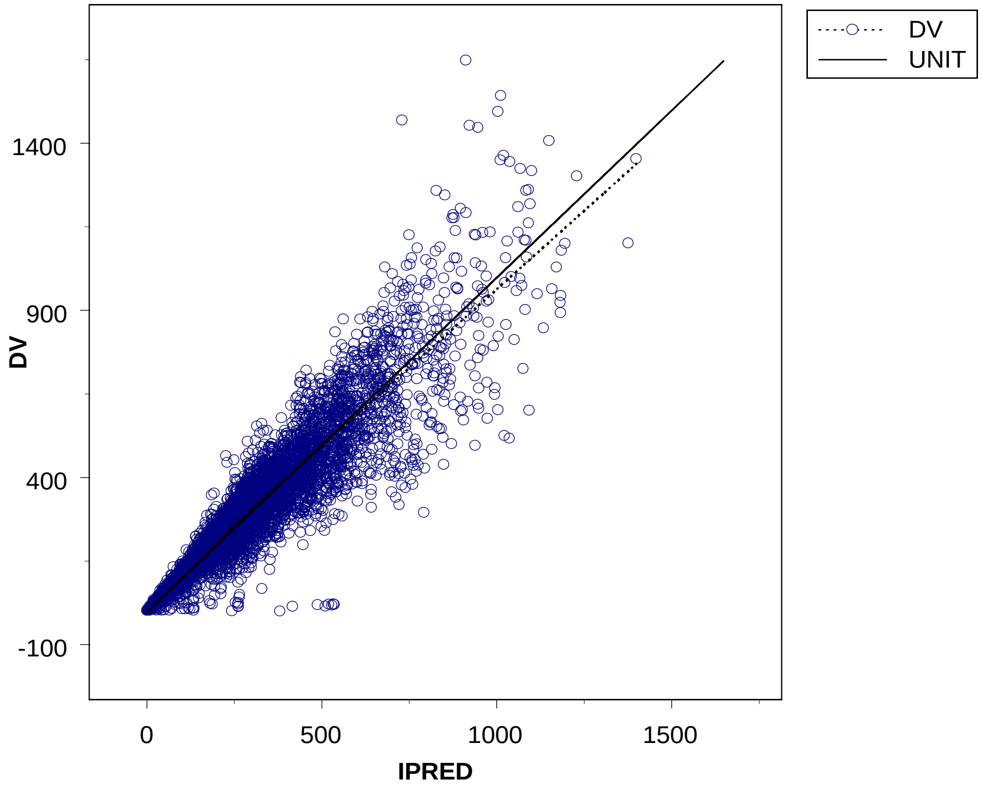

USARMYTAFPKC0409

Oct. 21, 2012 9:37:05 PM

Figure 8-2 Population Predicted (PRED) vs. Observed (DV) Tafenoquine Concentrations (Linear and Logarithmic Scales)

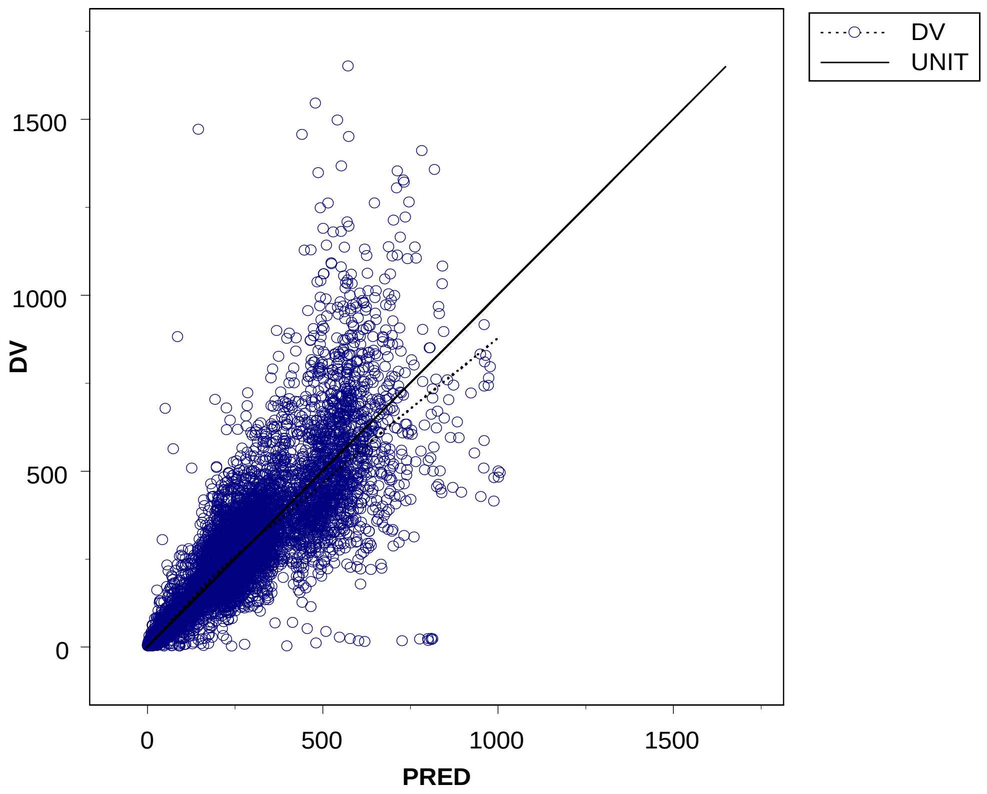

USARMYTAFPKC0409

Oct. 21, 2012 9:36:49 PM

Figure 8-3 Model-predicted vs. Observed Concentrations of Tafenoquine

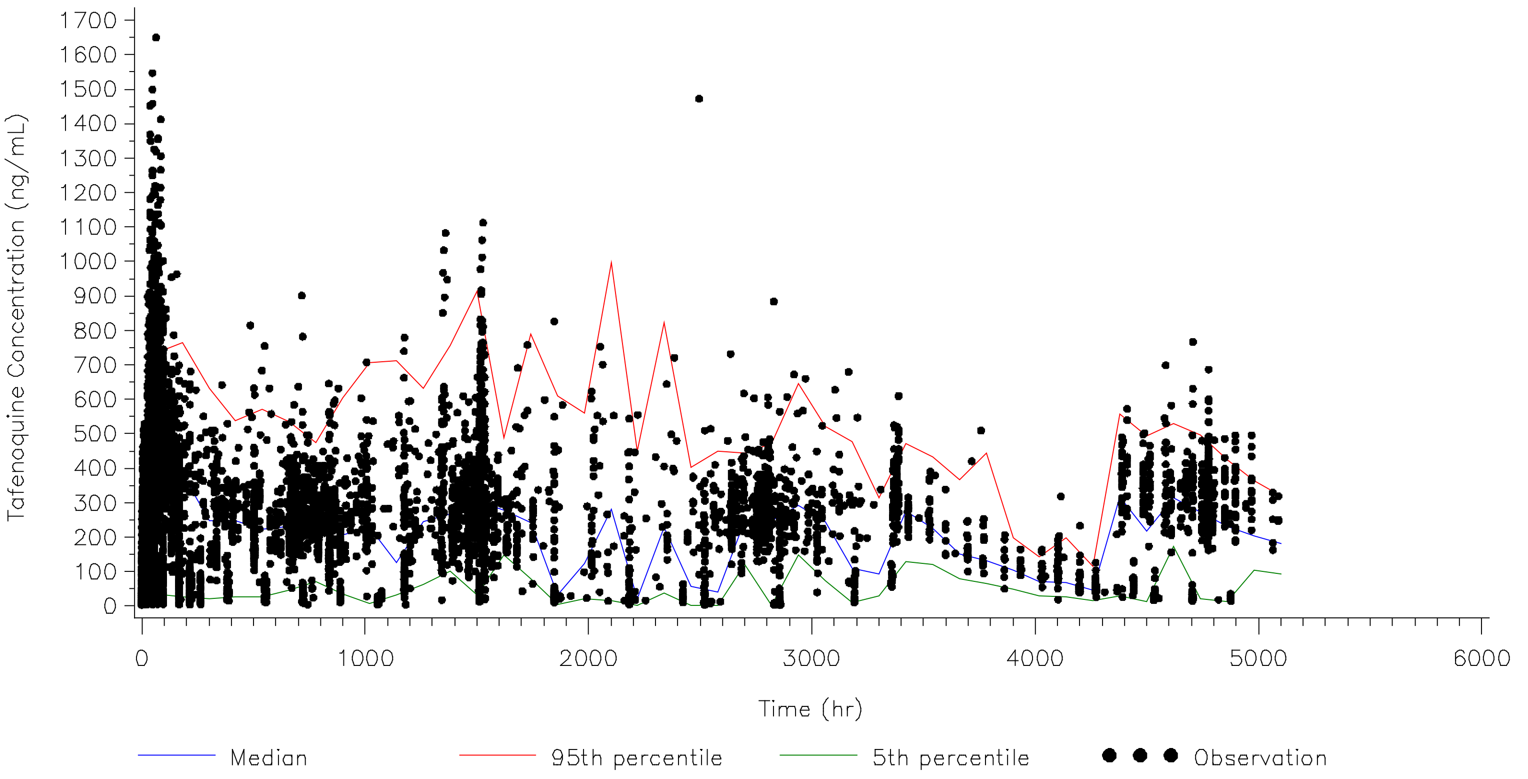

Source: Attachment 10.2

Conclusions:

- Pharmacokinetics of oral tafenoquine follows a one-compartment model with first order absorption and elimination
- Body weight and age were significant covariates with respect to CL/F of tafenoquine and CL/F was found to increase with increasing body weight and decreased with age
- Body weight and food were significant covariates with respect to V/F of tafenoquine and V/F was found to increase with increasing body weight and decreased with food

9. INTRODUCTION

Malaria is a protozoan infection of red blood cells (RBCs) that causes an acute febrile illness. Malaria caused by *P. falciparum* can be fatal. Manifestations of severe disease include cerebral, renal and/or gastrointestinal pathology as the parasitised RBCs become sticky and clump, blocking the capillaries. *P. vivax* and *P. ovale*, while rarely causing fatality cause substantial morbidity from recurrent relapses from liver hypnozoites.

Tafenoquine (SB-252263 and WR 38605), is a new aminoquinoline antimalarial drug being co-developed by GlaxoSmithKline (GSK), the Medicines for Malaria Venture (MMV), with historic support and assistance of the Walter Reed Army Institute of Research (WRAIR).

This population PK analysis was performed using data obtained from 10 Phase I/II/III clinical studies of tafenoquine (Army Study No's: 1, 2, 3, 4, 5, 14, 15, 33, 44, and 58).

10. OBJECTIVES

The objectives of this analysis were:

- to develop population pharmacokinetic (PK) model of tafenoquine using pooled PK data from 10 clinical studies and to assess the effect of covariates such as body weight, age, sex, race, and food on the PK characteristics of tafenoquine

Primary Endpoints

- Endpoints for population PK are population estimates of PK parameters (e.g., CL/F, V/F, Ka), associated inter-subject variability and residual error.

- Endpoints for the covariate analysis are the identification of significant covariates that were retained in the PK model and quantification of their effects.

11. METHODS

11.1. Study Design

Some key details of each of 10 studies used for this population PK modeling are given in Table 11-1. Data for this analysis was provided as validated NONMEM ready datasets for Army Study No's: 1, 2, 3, 4, 5, 14, 15, and 58. However, for Army Study No's: 33 and 44, unvalidated datasets were provided which were validated first using the raw datasets. Then, a confirmatory PK modeling was performed (Part 1 of this report) before incorporating the data into the pooled dataset.

Table 11-1 Clinical Studies of Tafenoquine

Army Study No.	GSK Study No.	Phase	Study Type	Study Design	Doses	Population	PK Collection
1	50	I	Single Dose Healthy Volunteer Study	Randomized, double-blind, placebo controlled, single oral dose rising, fasted	TQ 4 to 600mg or placebo	80 Male Healthy Volunteers (18-35y)	Before dosing and at 4, 8, 12, 24, 48, 72, 96, and 168 hours after dose. For the six higher dose groups (250, 300-600 mg), additional samples were collected on days 16, 23, 30, and 37.
2	52	I	Single Dose Healthy Volunteer Study	Randomized, parallel-group, single oral dose, fasted	TQ 100, 200 or 400mg	18 Male Healthy Volunteers (18-32y)	N/A
3	53	I	Malaria Challenge Study in Healthy Volunteers	Randomized, double-blind, placebo controlled, single dose, fasted, Malaria challenge study	TQ 600mg (n=4) or placebo (n=2) one day before sporozoite inoculation	4M/2F volunteers (19-30y)	Before dosing and at 5, 7, 12, 28, and 42 days after dosing on Day 1
4	51	I	Single and Repeat Dose Healthy Volunteer Study	Randomized, double-blind, placebo controlled, multiple dose (fasted for ≥2h)	TQ 200, 400, or 600mg weekly for 10 weeks or placebo	30M/6F (23-46y)	On Day 1 (Dose 1) and at Week 10 (Dose 10) before dosing and at 2, 4, 6, 8, 12, 16 and 24 h postdose. On Dose 10, trough blood samples were drawn pre-dose (weekly) prior to Doses 2 through 9 at weeks 12, 14, 16, 18, and 20.
5	54	I	Malaria Challenge Study in Healthy Volunteers	Randomized, double-blind, placebo controlled, multiple oral dose (fed)	TQ 600mg on Days (–3) and (–2) before sporozoite inoculation (Day 0), then 300mg on Days 3, 10, 17 and 24 or placebo	12M volunteers (19-36y)	Before dosing on Day 17 and Before dosing on Day 24 and at 26, 31, 38, 45, and 59 days after dosing on Day 24.
14	14	I	Relative Bioavailability	Randomized, relative	TQ 400mg OD for 3	43M/15F	Relative to the first dose,

Army Study No.	GSK Study No.	Phase	Study Type	Study Design	Doses	Population	PK Collection
			in Healthy Volunteers	bioavailability of 3 oral formulations	days (fed)	(21-60y)	blood samples were collected for pharmacokinetic analysis from each subject prior to dosing and up to 96 h post dose. Further blood samples were collected on an ambulatory basis on days 6, 7 and 8 and in weeks 4, 6, 8, 10, 12, 14, 16 and 18.
15	15	I	Drug-Drug Interaction Studies in Healthy Volunteers	Single sequence, desipramine interaction	TQ 400mg OD for 3 days (fed). Desipramine 100mg on morning of Day 1 and Day 11 (fasted).	20M/14F (25-60y)	Blood samples for determination of SB-252263 (tafenoquine) plasma concentrations were collected up to 96 hours after the second dose of desipramine (Day 11) and thereafter at 2 week intervals until 9 weeks after the last (third) dose of SB-252263.
33	33	III	Malaria Prophylaxis Study	Double-blind, randomized, mefloquine positive control	TQ 200mg x 3d, then 200mg weekly MQ 250mg x 3d, then 250mg weekly	Nonimmune Australian army troops 632M/22F (18-51y)	Prophylactic Phase (Day 2, Week 4, 8, 16, 26)
44	44	II	Malaria Prophylaxis Study	Double-blind, randomized, placebo controlled	TQ 400mg x 3d, then 400mg monthly	Nonimmune Royal Thai Army 104M Randomized to TQ and 101M Randomized to placebo	For the 104 soldiers on monthly tafenoquine prophylaxis, blood samples were collected after commencing the loading dose at about 8 h, 24 h, 48 h and 56 h and then at 3±4 day intervals up until the first monthly dose. After each monthly dose, samples

Army Study No.	GSK Study No.	Phase	Study Type	Study Design	Doses	Population	PK Collection
							were collected at about 8 h post-dose, mid-month and at the end of the monthly dose (trough plasma drug concentration). Following the last monthly dose samples were collected at about 4 h, 8 h, 12 h and 24 h and then at 3±4 day intervals for 2 months. At each blood collection, samples were obtained from 2 to 28 volunteers (mean±sd., 12.6±7.1). For the 31 soldiers on weekly prophylaxis, blood samples were collected after 2±22 weeks of medication (mean±sd., 11.8±6.8). Samples from this group were collected at about 12 h and 168 h post weekly medication and at 14 days, 21 days and 28 days after the last dose.
58	58	II	*P. vivax* Treatment Study	Randomized, active-control, double-blind, double-dummy	TQ 400mg OD for 3 days in Cohort 1and TQ 600mg OD for 1 day in Cohort 2	120 M/F (60 in each Cohort) (20-60y)	Daily for Day 0-7, Day 12-20, and Day 28-30

N/A: Not available; M: Male; F: Female; TQ: Tafenoquine; MQ: Mefloquine; OD: Once daily; 3d: 3 days;

Source: Investigator Brochure Ver. 09, 18 November 2011.

11.2. Data Assembly

11.2.1. Description of Input Data

All data used in the population PK analysis were obtained from Army Study No's: 1, 2, 3, 4, 5, 14, 15, 33, 44, and 58. A total of 866 subjects were included in the population PK analysis.

Tafenoquine concentrations, demographic information, and clinical laboratory results from 10 studies were used to build NONMEM input data for PK analysis. Description of the PK input data is presented in End-of-Text Table 8-1.

11.2.2. Handling of Input Data

The data were prepared for analysis using SAS Version 9.1.3 (SAS Institute Inc., Cary, NC). Actual dosing and actual sampling times, when available, were used for the analysis.

The following dosing data and PK sampling handling were applied to the NONMEM input data for PK analysis.

- Concentrations that were BQL or equal to LLOQ were excluded.
- Missing continuous covariates were replaced by population median value

11.2.3. Bioanalytical Methodology

Plasma samples were analyzed for tafenoquine concentrations using a validated liquid chromatography-tandem mass spectrometry method, with an LLOQ of either 1.00 ng/mL or 5.00 ng/mL across studies.

11.3. Data Analysis

Population PK and PKPD analyses were carried out using NONMEM Version 7.1.2, PDx-Pop Version 4.2 and Intel Visual Fortran Compiler Version 12 on a Microsoft Windows XP platform.

Successful runs were determined by the following:

i. Successful model convergence
ii. At least three significant digits for any parameter
iii. Non-singular covariance matrix
iv. Completion of the covariance step without warnings
v. Confidence intervals of structural parameters should not include zero
vi. Acceptable models should not lead to trends in the distribution of weighted residuals versus model predictions and versus independent variable

vii. Not over-sensitive to initial estimates.

For optimal model selection, the NONMEM diagnostic plots generated by PDx-Pop in conjunction with Microsoft Excel and S-PLUS during the preliminary runs were evaluated at each stage of model development for appropriateness of the initial estimates and final estimates of model parameters. The standard criteria of change in the minimum objective function value (ΔOFV) and review of the following diagnostics were carried out:

i. Plots of DV (observed plasma concentration/effect) versus PRED (population predicted values)

ii. Plots of DV versus IPRED (individual predicted values)

iii. Plots of WRES (weighted residuals) versus PRED

iv. Plots of WRES versus time

v. Plots of CWRES (conditional weighted residuals) versus PRED for PK

vi. Plots of CWRES versus time for PK

vii. Plots of DV/PRED/IPRED versus time by Patient

viii. Population parameter estimates that are reasonable and supported in the context of the structural model, percent relative standard error (%RSE) and 95% confidence interval (CI) for the parameter estimates

ix. Value of the objective function (smaller is better)

x. Model stability based on changing of the number of significant digits specified with the estimation method and changing of the initial estimates of the parameters

Potential covariates affecting the PK of tafenoquine were explored. The procedure for covariate model building is detailed in Section 11.4.3.

11.4. Modeling Assumptions

11.4.1. Description of PK Model

Based on the pharmacokinetic profile of tafenoquine from previous modeling (as described in Part 1 of this report), a one-compartment PK model with first order absorption and elimination processes was selected to describe the PK of tafenoquine. As part of the modeling process, a two-compartment PK model with first order absorption and elimination process was also tested (Attachment 7). A schema of the one-compartment PK model is displayed in Figure 4-1.

This one-compartment PK model was specified in the NONMEM control file and was parameterized in terms of CL/F, V/F, Ka using the PREDPP ADVAN2 with TRANS2 subroutine. First order conditional estimation (FOCE) with interaction between variance of inter-individual variability and the variance of residual error was used as the estimation method.

11.4.2. Variability Models

11.4.2.1. Model for inter-individual variability

Pharmacokinetic

Between-individual variability (ETA or η), which is the difference between the individual parameter estimate and the population mean estimate of final PK parameters, was described by an exponential error model as described here:

$$Pi = \hat{P} \exp(\eta_i)$$

where:

Pi is the estimated parameter for the ith individual.

$\hat{P}$ is the population value for the parameter.

η_i are individual inter-individual random effects for the ith patient and parameter P and are distributed as follows: $\eta \sim N(0, \omega^2)$, and ω^2 is the variance of the inter-individual random effect.

11.4.2.2. Model for residual variability

Different structural models for residual variability were tested.

Pharmacokinetic

Residual variability (EPSILON or ε), which is the difference between the observed and the individual predicted observations of the final PK model, was described by a proportional error model as shown below:

$$C_{ij} = \hat{C}_{ij}(1 + \varepsilon_{pij})$$

Where:

C_{ij} is the jth observation in the ith individual.

$\hat{C}_{ij}$ is the jth predicted value in the ith individual.

ε_{pij} is the proportional residual random error for the ith individual and the jth measurement and is distributed as follows: $\varepsilon \sim N(0, \sigma^2)$.

11.4.3. Covariate Analysis

Covariate analysis was performed to the base PK model to identify potential covariates and to evaluate the extent to which the covariates accounted for the variability in the PK parameters.

Prior to including covariates in the population model, visual inspection of the relationship between each ETA and covariate was performed using scatter plots (End-of-Text Figure 16-21). The scatter plots were used to provide visual identification of collinearity between the covariates of interest. Covariates that were identified to demonstrate collinearity were not allowed to enter the covariate model at the same time. A decision on inclusion of covariates in the final model was also based on whether they made physiological sense. The following variables were selected for evaluation as potential covariates of CL/F, V/F, and Ka: sex, age, race, weight (WT).

For covariate selection/elimination, the following steps were followed:

- Selection of the simplest structural model, to use as a valid base model, based on smallest objective function and by inspection of the pattern in the residual plots. The best estimation method, the most appropriate between-subjects variance models, and the residual error model, were identified. The resulting model was called a BASE model.

- Selection of covariates by univariate analyses, at risk 0.05 (reduction of objective function of at least 3.84). The best model for each covariate to affect each of the parameters was selected at this stage.

- Multivariate analysis: all selected covariates were added together and the model fitted to data. This reference was called FULL model.

- Backward deletion was applied until no covariate could be removed without significantly increasing the objective function, resulting in the FINAL model (likelihood ratio test at $p<0.001$, 1 df, objective function drop of at least 10.83).

- If a confidence interval (CI) of structural parameters included the value zero, the effect was considered not significant and the model was further simplified until all structural parameters were well estimated.

Continuous covariates in the model were centered on the population median value of the subjects/patients included in the analysis and are described in more detail in the results section.

11.5. Model Evaluation

The final PK model was evaluated using bootstrapping and a posterior predictive check.

11.5.1. Bootstrapping

Bootstrapping is data re-sampling method for estimating sampling variances, confidence intervals, and stability of regression models. Using the bootstrap approach, the bootstrap parameter values are obtained by repeatedly fitting the final population model to a reasonable number of bootstrap samples. The mean and confidence interval (CI) values of the bootstrap parameters are then compared to the final population model parameter estimates and associated CIs from NONMEM.

Bootstrap analysis procedure consisted of the following steps:

1. The replication of the data file is obtained by random draw (with replacement) from the original data file.

2. The final model is fitted to the resulting data file, and the model parameter estimates are saved for the final analysis.

3. Steps 1 and 2 are repeated 100-1000 times (with different random draws)

4. Distribution of the parameter estimates are plotted and visually analyzed.

5. Bootstrap parameter estimates, standard errors and CIs are obtained.

The PDx-Pop Model Evaluation module automated the bootstrap analysis, computation of bootstrap estimates, variance, and CIs and plotting of histogram of the parameter estimates. The 95% bootstrap CIs were determined for the PK parameters derived from 1000 bootstrap datasets and compared to the original parameters obtained from the final model (PDx-Pop manual)[4].

11.5.2. Posterior predictive check

A posterior predictive check was performed by simulating the tafenoquine concentrations from the original datasets using the parameter estimates obtained in the final PK modeling steps. One thousand (1000) predicted profiles were simulated for each original subjects/patients. Random effects were included in the simulation. The median, 5th percentile, and 95th percentile PK concentrations versus time profiles from the simulations were compared with observed tafenoquine concentrations.

11.6. Changes in Planned Analyses

None.

12. RESULTS

12.1. Analysis Population and Data Characteristics

A total of 866 subjects were included in the population PK. A breakdown of subjects/patients by study is given in Table 12-1. More details of demographic information are presented in End-of-Text Table 16-4 to End-of-Text Table 16-14.

Table 12-1 Number of Subjects/Patients in the Population PK Analysis Population by Study

Army Study No.	Number of Subjects/Patients
1	45
2	18
3	4
4	25
5	10
14	58
15	34
33	491
44	135
58	46

Since oral tafenoquine displayed mono-exponential disposition in the previous population PK modeling as described in Part 1, a one-compartment PK model with first order absorption and elimination was used as a starting point for the base PK model in the population PK analysis.

12.2. Modeling Results

12.2.1. Base PK Model

Based on the previous modeling experience, one-compartment PK model with first order absorption and elimination rate constants was selected as the structural model. Different error models for inter-individual and residual variability were also tested. The exponential error model was finally chosen to describe inter-individual variability of each PK parameter and the proportional error model was finally chosen to describe residual error. The base PK model was chosen based on the criteria in Section 11.3. Description of key models tested during model searches is given in Table 12-2.

A two-compartment PK model was also tried but was not pursued further because unreliable estimates from bootstrap results (Attachment 7).

Table 12-2 Description and Evaluation of Key PK Models Tested

Primary Model Structure	ETA Model	Residual Error	Model No	Data File	OFV	MIN	Boundary	COV
All 3 ETAs	Exponential All	Additive	USARMYTAFPKB0201	USTAFCMB10OCT12.csv	72958.76	Y	N	Y
	Exponential All	Proportional	USARMYTAFPKB0202	USTAFCMB10OCT12.csv	68057.94	Y	N	Y
	Exponential All	Exponential	USARMYTAFPKB0203	USTAFCMB10OCT12.csv	68057.94	Y	N	Y
	Exponential All	Additive + Proportional	USARMYTAFPKB0204	USTAFCMB10OCT12.csv	68034.8	Y	N	Y
All 3 ETAs	CL, V exponential KA additive	Additive	USARMYTAFPKB0205	USTAFCMB10OCT12.csv	72974.49	Y	N	Y
	CL, V exponential KA additive	Proportional	USARMYTAFPKB0206	USTAFCMB10OCT12.csv	68091.73	Y	N	Y
	CL, V exponential KA additive	Exponential	USARMYTAFPKB0207	USTAFCMB10OCT12.csv	68095.66	Y	N	Y
	CL, V exponential KA additive	Additive + Proportional	USARMYTAFPKB0208	USTAFCMB10OCT12.csv	68073.39	Y	N	Y
ETAs on CL and V only	Exponential All	Additive	USARMYTAFPKB0209	USTAFCMB10OCT12.csv	73152.82	Y	N	Y
	Exponential All	Proportional	USARMYTAFPKB0210	USTAFCMB10OCT12.csv	68381.44	Y	N	Y
	Exponential All	Exponential	USARMYTAFPKB0211	USTAFCMB10OCT12.csv	68381.44	Y	N	Y
	Exponential All	Additive + Proportional	USARMYTAFPKB0212	USTAFCMB10OCT12.csv	68366.55	Y	N	Y
All 3 ETAs (Full Omega Block)	Exponential All	Additive	USARMYTAFPKB0213	USTAFCMB10OCT12.csv	72958.76	Y	N	AB
	Exponential All	Proportional	USARMYTAFPKB0214	USTAFCMB10OCT12.csv	68057.94	Y	N	AB
	Exponential All	Exponential	USARMYTAFPKB0215	USTAFCMB10OCT12.csv	68057.94	Y	N	AB
	Exponential All	Additive + Proportional	USARMYTAFPKB0216	USTAFCMB10OCT12.csv	68034.8	Y	N	AB
All 3 ETAs Omega block on CL and V	Exponential All	Additive	USARMYTAFPKB0217	USTAFCMB10OCT12.csv	72955.77	Y	N	Y
	**Exponential All**	**Proportional**	**USARMYTAFPKB0218 (selected as Base PK Model)**	**USTAFCMB10OCT12.csv**	**67844.96**	**Y**	**N**	**Y**
	Exponential All	Exponential	USARMYTAFPKB0219	USTAFCMB10OCT12.csv	67844.96	Y	N	Y
	Exponential All	Additive + Proportional	USARMYTAFPKB0220	USTAFCMB10OCT12.csv	67830.24	Y	N	Y
All 3 ETAs Omega block on V and Ka	Exponential All	Additive	USARMYTAFPKB0221	USTAFCMB10OCT12.csv	72915.97	Y	N	Y
	Exponential All	Proportional	USARMYTAFPKB0222	USTAFCMB10OCT12.csv	68048.72	Y	N	Y
	Exponential All	Exponential	USARMYTAFPKB0223	USTAFCMB10OCT12.csv	68048.72	Y	N	Y
	Exponential All	Additive + Proportional	USARMYTAFPKB0224	USTAFCMB10OCT12.csv	68025.18	Y	N	Y
All 3 ETAs Omega block on CL and Ka	Exponential All	Additive	USARMYTAFPKB0225	USTAFCMB10OCT12.csv	72955.76	Y	N	Y
	Exponential All	Proportional	USARMYTAFPKB0226	USTAFCMB10OCT12.csv	68046.16	Y	N	Y
	Exponential All	Exponential	USARMYTAFPKB0227	USTAFCMB10OCT12.csv	68046.16	Y	N	Y
	Exponential All	Additive + Proportional	USARMYTAFPKB0228	USTAFCMB10OCT12.csv	68022.27	Y	N	Y
ETAs on CL and V only Omega block	Exponential All	Additive	USARMYTAFPKB0229	USTAFCMB10OCT12.csv	73143.67	Y	N	Y
	Exponential All	Proportional	USARMYTAFPKB0230	USTAFCMB10OCT12.csv	68161.42	Y	N	Y
	Exponential All	Exponential	USARMYTAFPKB0231	USTAFCMB10OCT12.csv	68161.42	Y	N	Y
	Exponential All	Additive + Proportional	USARMYTAFPKB0232	USTAFCMB10OCT12.csv	68152.38	Y	N	Y

Min: Successful Minimization; BOUND: Parameter near boundary; Cov: Successful covariance.

Source: Attachment 7.2

Once one-compartment PK model with first order absorption and elimination was selected as the structural PK model, several more models were explored for inter-individual variability and residual variability. In early attempts, when inter-individual variability terms were included for all three PK model parameters: CL, V, and Ka, p-value of mean ETABAR of Ka was statistically significant indicating that the true mean of ETA on Ka was different from 0. Before dropping the inter-individual variability term for Ka, the robustness of the model with inter-individual variability on Ka was tested using both bootstraps as well as visual predictive check plots. Since results clearly showed that inter-individual variability could be estimated with reasonable precision, a decision was made continue further modeling including inter-individual variability for all 3 parameters.

As shown in Table 12-3, models with covariance between CL and V variances (Models: USARMYTAFPKB0218, USARMYTAFPKB0219, USARMYTAFPKB0220, and USARMYTAFPKB0221) resulted in the smallest OFV. Out of these 4 models, the model USARMYTAFPKB0220 had the smallest OFV. However, it was not selected as the Base PK model not only because of over-prediction of concentrations as seen in visual predictive check plots and also based on the bootstrap parameter distribution (Attachment 7). In all of the models explored, ETA shrinkage on CL and V was below 15% and was ~55% for Ka. After comparing predictive check results and bootstrap results (Attachment 5, Attachment 6, and Attachment 7), the model USARMYTAFPKB0218 was eventually selected as the Base PK model.

The population parameter estimates for the base PK model are summarized in (End-of-Text Table 16-2). NONMEM control file, output, and diagnostic plots for the base PK model are provided in Attachment 4.

12.2.2. Final PK Model

Covariates selection was carried out as described in Section 11.4.3. Potential covariates for the base PK model were evaluated by examining scatter plots of inter-individual variability (ETA) of PK parameter versus the covariates, the correlations among the covariates. Description of key covariate models tested during model searches is given in Table 12-3 and Table 12-4.

Because age, body weight, RACE, SEX, and FOOD were the only covariates present for all 10 studies, these covariates were selected for covariate model exploration. Each of these covariates was included in the Base PK model (Table 12-3) to test for their significance. Since SEX and RACE were confounded with body weight, they were not explored in the full covariate model. Since distribution of races other than Caucasian and Asian was small (<20%), race effect was explored only for Asian versus Caucasians/others as the reference.

Table 12-3 Description and Evaluation of Key Individual Covariate PK Models Tested

No.	Model No	Model Description	OFV	ΔOFV	MIN	BOUND	COV	P<0.05
0	USARMYTAFPKB0218	Base PK Model	67844.955	0	Y	N	Y	-
1	USARMYTAFPKC0201	WT on CL	67817.089	-27.866	Y	N	Y	Y
2	USARMYTAFPKC0202	WT on V	67738.273	-106.682	Y	N	Y	Y
3	USARMYTAFPKC0203	WT on Ka	67844.85	-0.105	Y	N	Y	N
4	USARMYTAFPKC0204	AGE on CL	67810.799	-34.156	Y	N	Y	Y
5	USARMYTAFPKC0205	AGE on V	67824.215	-20.74	Y	N	Y	Y
6	USARMYTAFPKC0206	AGE on Ka	67838.048	-6.907	Y	N	Y	Y
7	USARMYTAFPKC0207	SEX on CL	67842.014	-2.941	Y	N	Y	N
8	USARMYTAFPKC0208	SEX on V	67829.661	-15.294	Y	N	Y	Y
9	USARMYTAFPKC0209	SEX on Ka	67844.941	-0.014	Y	N	Y	N
10	USARMYTAFPKC0210	RACE on CL	67795.763	-49.192	Y	N	Y	Y
11	USARMYTAFPKC0211	RACE on V	67777.084	-67.871	Y	N	Y	Y
12	USARMYTAFPKC0212	RACE on Ka	67840.349	-4.606	Y	N	Y	Y
13	USARMYTAFPKC0213	FOOD on CL	67842.201	-2.754	Y	N	Y	N
14	USARMYTAFPKC0214	FOOD on V	67800.944	-44.011	Y	N	Y	Y
15	USARMYTAFPKC0215	FOOD on Ka	67832.048	-12.907	Y	N	Y	Y

Min: Successful Minimization
BOUND: Parameter near boundary
Cov: Successful covariance
ΔOFV: Difference in OFV from Base PK model (USARMYTAFPKB0218).
Any individual covariate that resulted in a reduction of objective function of at least 3.84 was taken forward to the full model.
Source: Attachment 7.3

Table 12-4 Description and Evaluation of Key Individual Covariate PK Models Tested

No.	Model No	Model Description	OFV	ΔOFV	MIN	BOUND	COV	P<0.001
16	USARMYTAFPKC0401 (Full PK Model)	WT on CL and V<br>AGE on CL, V, and KA<br>FOOD on V and KA	67517.068	0	Y	N	Y	-
17	USARMYTAFPKC0402	WT on CL dropped from Model 16	67641.404	124.336	Y	N	Y	Y
18	USARMYTAFPKC0403	WT on V dropped from Model 16	67710.242	193.174	Y	N	Y	Y
19	USARMYTAFPKC0404	AGE on CL dropped from Model 16	67540.211	23.143	Y	N	Y	Y
20	USARMYTAFPKC0405	AGE on V dropped from Model 16	67521.861	4.793	Y	N	Y	N
21	USARMYTAFPKC0406	AGE on KA dropped from Model 16	67527	9.932	Y	N	Y	Y
22	USARMYTAFPKC0407	FOOD on V dropped from Model 16	67563.393	46.325	Y	N	Y	Y
23	USARMYTAFPKC0408	FOOD on KA dropped from Model 16	67523.458	6.39	Y	N	Y	N
24	USARMYTAFPKC0409 (selected as Final PK Model)	WT on CL and V<br>AGE on CL<br>FOOD on V	67539.357	22.289	Y	N	Y	-

Min: Successful Minimization
BOUND: Parameter near boundary
Cov: Successful covariance
ΔOFV: Difference in OFV from Full Covariate PK model (USARMYTAFPKC0401).
If dropping a covariate from Full Covariate PK model resulted in an increase of OFV by 10.83, that covariate was retained in the final model.
Source: Attachment 7.4

Body weight on CL and V, age on CL, V, Ka and FOOD on V, Ka were included in the full covariate PK model (Table 12-4). Full covariate PK model was further reduced by backward elimination by dropping one covariate at a time. The results from this process of backward elimination are presented in Table 12-4. Using the criteria in Section 11.4.3, the final PK model was reached with body weight and age as covariates of CL/F and body weight and food as covariates of V/F.

The relationship of covariates with CL/F and V/F is summarized in (Table 12-5).

Table 12-5 Population PK Parameters of Final PK Model

Parameters (Units)	Final Estimate	Bootstrap 95% CI		Between-individual Variability [a]
		Lower	Upper	
CL/F (L/hr) = $\theta_{CL} \times (WT/75)^{\theta_{CL\text{-}WT}} \times (AGE/25)^{\theta_{CL\text{-}AGE}}$				23.6%
θ_{CL}	4.17	4.080	4.230	
$\theta_{CL\text{-}WT}$	0.552	0.474	0.637	
$\theta_{CL\text{-}AGE}$	-0.200	-0.267	-0.138	
V/F (L) = $\theta_{V} \times (WT/75)^{\theta_{V\text{-}WT}} \times (AGE/25 \times (\theta_{V\text{-}FOOD})^{FOOD}$				24.1%
θ_{V}	2470	2340	2630	
$\theta_{V\text{-}WT}$	0.781	0.652	0.901	
$\theta_{V\text{-}FOOD}$	0.822	0.761	0.861	
Ka (1/hr)	0.359	0.321	0.384	54.1%

b. The magnitude of inter-individual variability was presented as CV%.
Note: Final estimate and inter-individual variability were from NONMEM estimates.
Source: Attachment 8.2 (USARMYTAFPKC0409.sum)

For oral tafenoquine, the population CL/F and V/F are determined to be 4.17 L/hr and 2470 L, respectively. The first order absorption rate constant of oral tafenoquine was 0.359 1/hr. The inter-individual variability of CL/F, V/F, and Ka was 23.6%, 24.1% and 54.1%, respectively.

The final PK model revealed that apparent clearance (CL/F) of tafenoquine is a function of body weight (WT) and age. These covariates accounted for 2.9% of inter-individual variability of CL/F (i.e., decrease inter-individual variability from 26.5% to 23.6%). The relationship between CL/F and both covariates was:

$$CL/F\ (L/hr) = 4.17 \times (WT/75)^{0.552} \times (AGE/25)^{-0.2}$$

Thus, tafenoquine CL/F was found to increase with increasing body weight and decreased with age.

The mean estimate of CL/F for an individual with a median age of 25 years in the lowest body weight (43 kg) could be as low as 0.97-fold of the average CL/F. The mean estimate of

CL/F in the highest body weight (135 kg) could be as large as 1.03-fold of the average CL/F (Table 12-5).

The mean estimate of CL/F for an individual with a median weight of 75 kilograms in the younger age category (18 years) could be 1.07-fold of the average CL/F. The mean estimate of CL/F in the older age category (60 years) could be 0.84-fold of the average CL/F (Table 12-5).

The final PK model revealed that apparent volume of distribution (V/F) of tafenoquine is a function of body weight (WT) and food. These covariates accounted for 5.5% of inter-individual variability of V/F (i.e., decrease inter-individual variability from 29.6% to 24.1%). The relationship between V/F and both covariates was:

$$V/F\ (L) = 2470 \times (WT/75)^{0.781} \times (0.822)^{FOOD}$$

Where FOOD = 0 for fasted and 1 for fed

Tafenoquine V/F was found to increase with increasing body weight and decreased with food.

The mean estimate of V/F for a fasted individual in the lowest body weight (43 kg) could be as low as 0.65-fold of the average V/F. The mean estimate of V/F in the highest body weight (135 kg) could be as large as 1.58-fold of the average V/F (Table 12-5). For individual with a median body weight of 75 kilograms, V/F of tafenoquine under fed conditions is 17.8% lower than the typical individual (2030 L vs. 2470 L).

Selected diagnostic plots and end-of-text references are presented in Table 12-6. Complete results of the final PK model and diagnostic plots are in Attachment 8.

Table 12-6 List of Diagnostic Plots for the Final PK Model

Plot Name	End-of-Text Reference
DV vs. IPRED	End-of-Text Figure 16-1
DV vs. PRED	End-of-Text Figure 16-2
RES vs. PRED	End-of-Text Figure 16-3
RES vs. Time	End-of-Text Figure 16-4
RES vs. TAD	End-of-Text Figure 16-5
WRES vs. PRED	End-of-Text Figure 16-6
WRES vs. Time	End-of-Text Figure 16-7
WRES vs. TAD	End-of-Text Figure 16-8
WRES vs. ID	End-of-Text Figure 16-9
Absolute IWRES vs. IPRED	End-of-Text Figure 16-10
Predicted vs Observed concentrations	End-of-Text Figure 16-11
CWRES vs. PRED	End-of-Text Figure 16-12

CWRES vs. Time	End-of-Text Figure 16-13
DV, PRED, IPRED vs. Time (Linear Scale)	End-of-Text Figure 16-14
DV, PRED, IPRED vs. Time (Semi-Logarithmic Scale)	End-of-Text Figure 16-15
DV, PRED, IPRED vs. TAD (Linear Scale)	End-of-Text Figure 16-16
DV, PRED, IPRED vs. TAD (Semi-Logarithmic Scale)	End-of-Text Figure 16-17
Plot of WRES vs. Covariates (Final PK Model)	End-of-Text Figure 16-18
Distribution of Individual Estimate of ETA (Final PK Model)	End-of-Text Figure 16-19
Plot of Correlation Matrix of ETA (Final PK Model)	End-of-Text Figure 16-20
Plot of ETA vs. Covariates (Final PK Model)	End-of-Text Figure 16-21

Routine diagnostic weighted residuals versus population model-predicted values (data not shown) were symmetrically indicating a good fit of the model to the data.

12.3. Model Evaluation Results

12.3.1. Bootstrapping

The bootstrapping technique was used to evaluate the final PK and PKPD models. One thousand (1000) bootstrap samples for PK were employed to evaluate the final model. The bootstrap estimates were derived for the PK and compared to the original parameters obtained for the final model. Results of the PK model evaluation are presented in Attachment 9. Histograms of bootstrap PK parameter estimates are displayed in Attachment 9.2.

Table 12-7 compares parameter estimates from the bootstrap to NONMEM estimates. Table 12-8 compares estimates of the variability of the random effects from the bootstrap to the corresponding NONMEM estimates. It had been proposed by Ette EI *et al* that if the parameter estimates from the bootstrap are within ±15% of those of final model, the parameters from the final model could be considered reliable [5, 6]. In this study, the differences of mean bootstrap estimates from the NONMEM estimates of those parameters were less than 5%, demonstrating a satisfactory level of reliability of the final PK model. Overall, mean population PK parameter estimates and 95% CI obtained from the bootstrap procedure were generally comparable to the estimates and 95% CI from the final PK model.

The success rate of bootstrap runs was 100% for PK model.

Table 12-7 Comparison of Bootstrap and NONMEM Parameter Estimates

Parameters	Bootstrap Estimate	NONMEM Estimate	Difference [a]
**PK Model**			
θ_{CL}	4.17	4.15445	-0.37%
$\theta_{CL\text{-}WT}$	0.552	0.553913	0.35%
$\theta_{CL\text{-}AGE}$	-0.2	-0.2001639	0.08%
θ_{V}	2470	2481.52	0.47%
$\theta_{V\text{-}WT}$	0.781	0.773541	-0.96%
$\theta_{V\text{-}FOOD}$	0.822	0.809538	-1.52%
θ_{KA}	0.359	0.350768	-2.29%

[a] Expressed as percent of difference between Bootstrap and NONMEM estimates from the final model ([Bootstrap/NONMEM-1]*100%).
Source: Attachment 8 to Attachment 9.

Table 12-8 Comparison of Bootstrap and NONMEM Estimates of Random Effects

Parameters	Bootstrap Estimate	NONMEM Estimate	Difference [a]
PK Model			
ω11	0.0555	0.0535898	-3.44%
ω21	0.0289	0.0250287	-13.40%
ω22	0.0583	0.0521484	-10.55%
ω33	0.293	0.282772	-3.49%
σ11	0.0488	0.0485538	-0.50%

OMEGA is the variance of the inter-individual random effect.
SIGMA is the variance of the residual random effect.
[a]: Expressed as percent of difference between Bootstrap and NONMEM estimates from the final model ([Bootstrap/NONMEM-1]*100%).
Source: Attachment 8 to Attachment 9.

12.3.2. Posterior Predictive Check Results

Simulations were performed using NONMEM parameter estimates from the final PK and PKPD models and were carried out for the original datasets. One thousand (1000) simulations for each original subject/patient were carried out. The NONMEM control file used for the simulation of tafenoquine is given in Attachment 10. Median, 5th percentile, and 95th percentile plot of model-predicted vs. observed concentrations is presented in Figure 12-1 for tafenoquine. Figure 12-1 demonstrates the adequacy of final PK model to reproduce a majority of tafenoquine concentrations over the course of several dose levels. Most of the observations were within the 90% confidence interval (the 5th and 95th percentiles).

Figure 12-1 Model-Predicted vs. Observed Tafenoquine Concentration versus Time Profiles (Final PK Model)

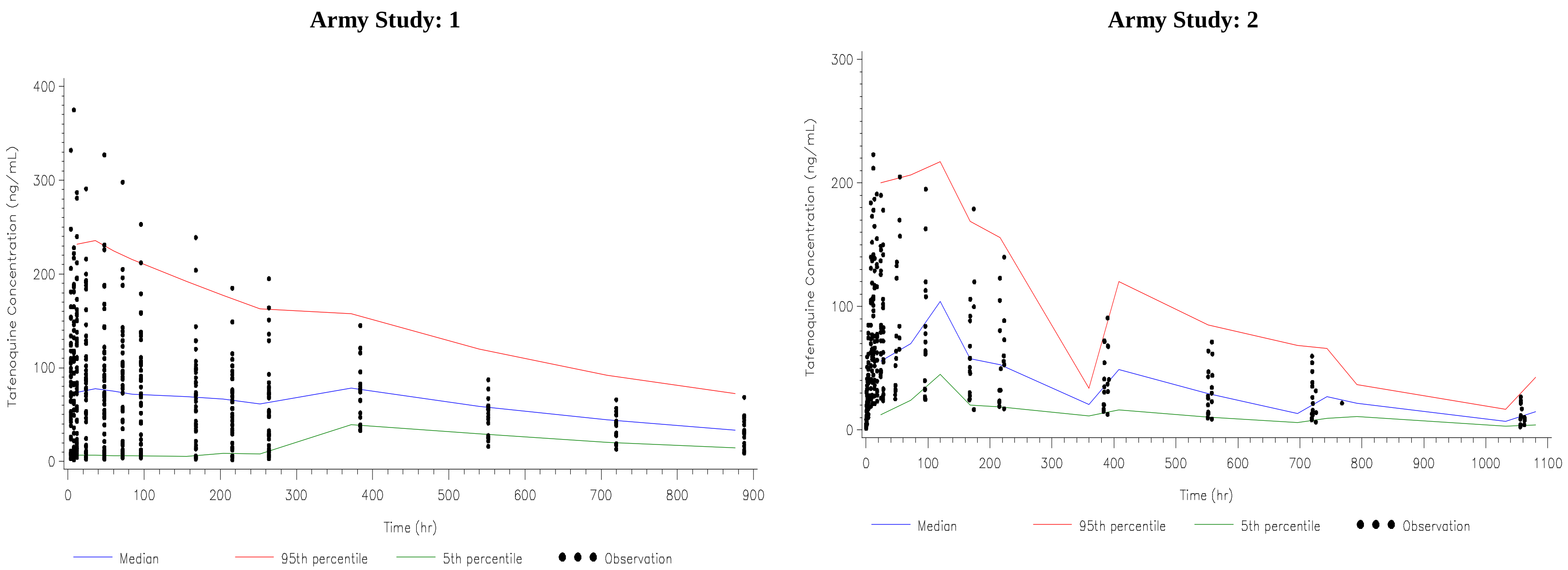

Figure 12-1 Model-Predicted vs. Observed Tafenoquine Concentration versus Time Profiles (Final PK Model) (Cont'd)

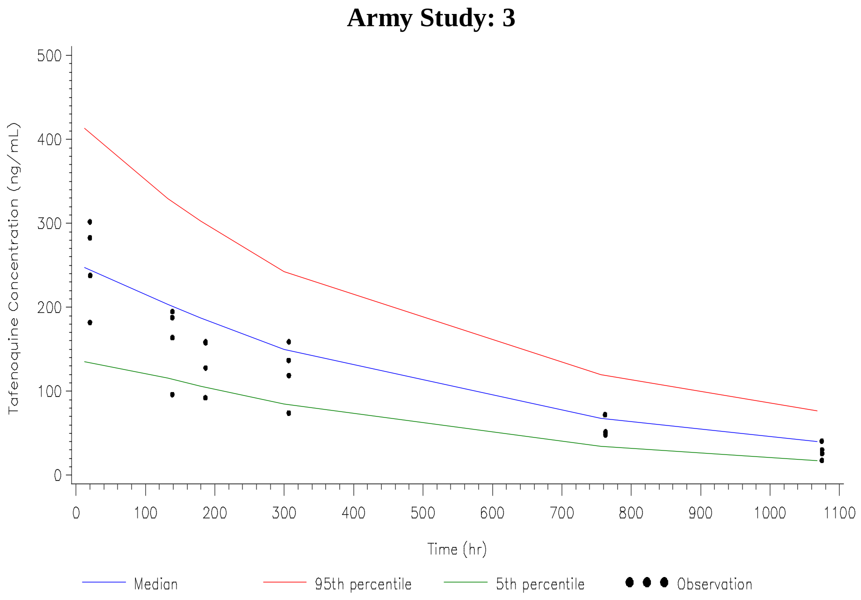

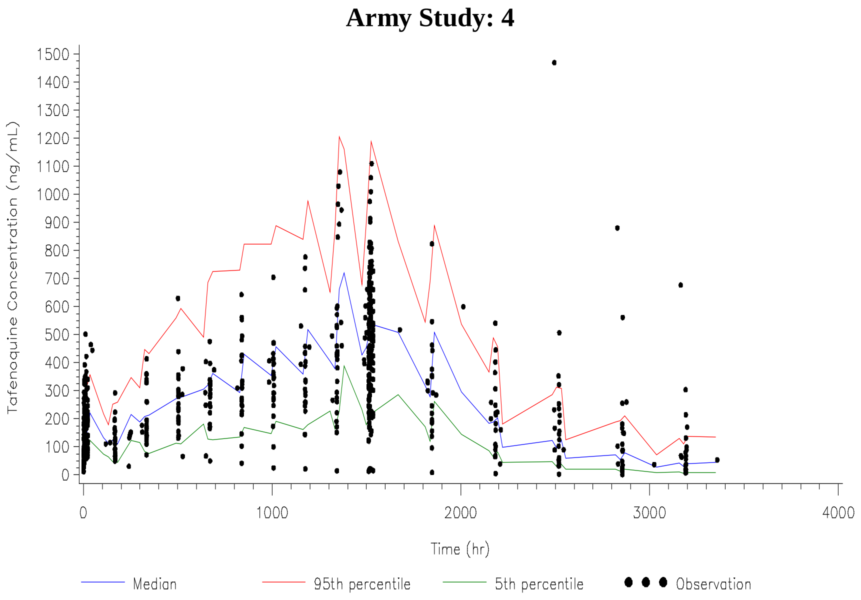

Source: Attachment 10.2

Figure 12-1 Model-Predicted vs. Observed Tafenoquine Concentration versus Time Profiles (Final PK Model) (Cont'd)

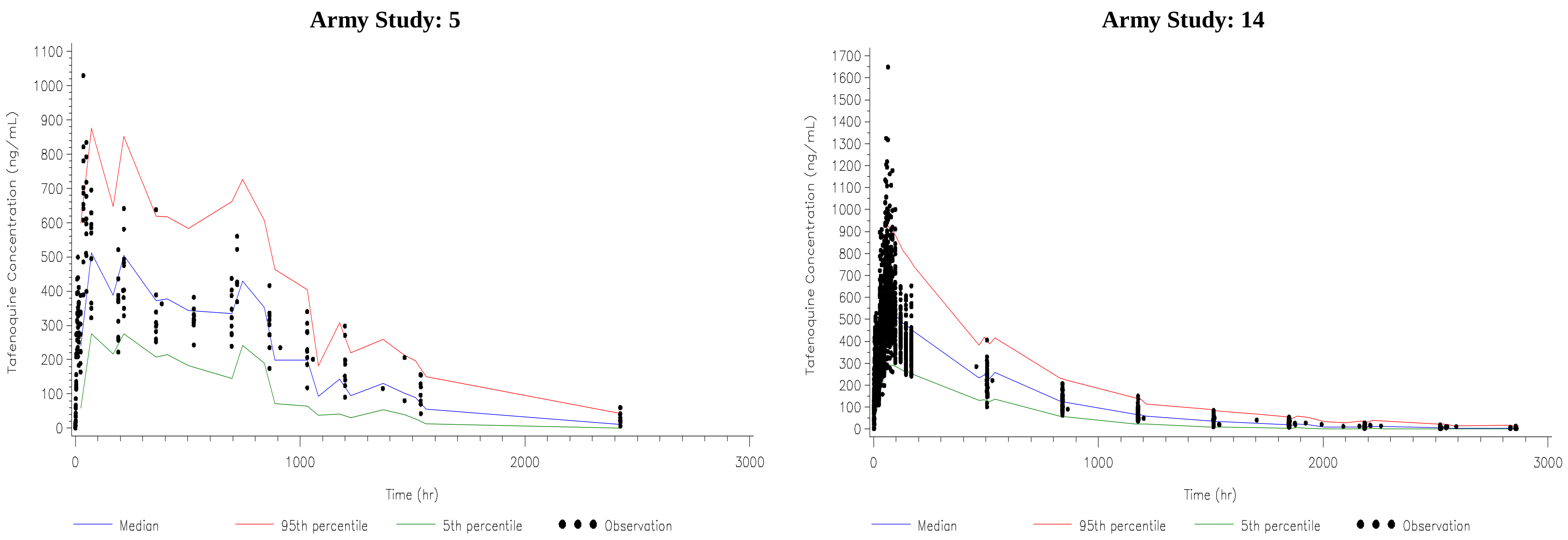

Source: Attachment 10.2

Figure 12-1 Model-Predicted vs. Observed Tafenoquine Concentration versus Time Profiles (Final PK Model) (Cont'd)

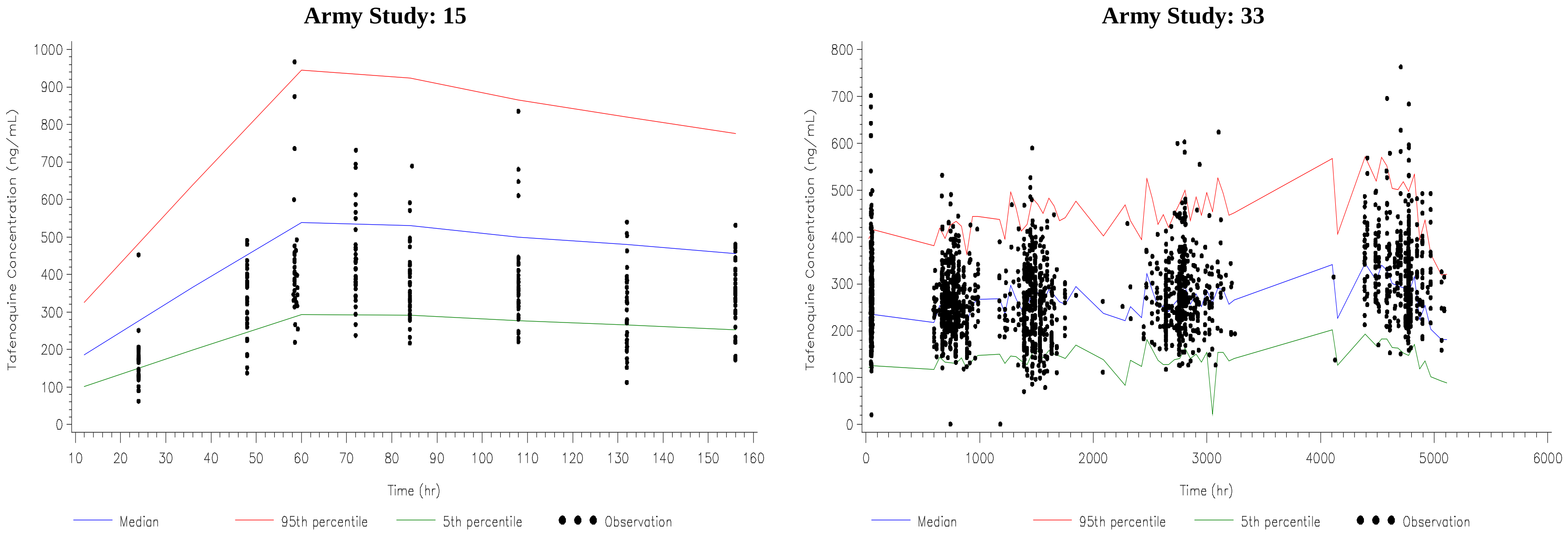

Source: Attachment 10.2

Figure 12-1 Model-Predicted vs. Observed Tafenoquine Concentration versus Time Profiles (Final PK Model) (Cont'd)

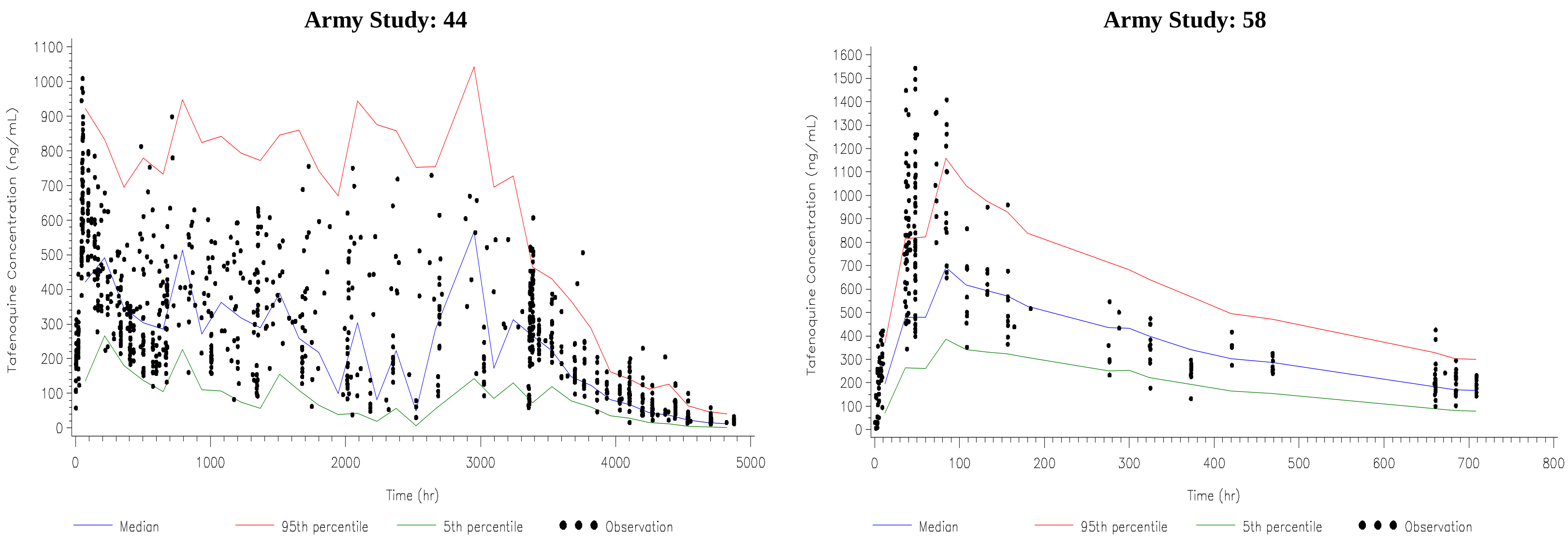

Source: Attachment 10.2

13. SIMULATIONS

Simulation of PK data for various doses and dose regimens were carried out using the final model parameter estimates (fixed effect, random effect and residual error). In general, the simulation step included creation of data files using dummy subjects with desired sampling times and dosing regimen, running simulation with desired number of replicates using the final model output parameters (THETAs, ETAs and SIGMAs) in the control file. The output from the simulations was summarized using SAS and presented graphically in Phoenix WinNonlin. The decision on the dosing regimen to be simulated and the number of simulations to be conducted was based on the instructions provided by US Army.

Following dosing scenarios were simulated.

- A reference profile (Figure 13-1)
  - Three 200 mg once-daily loading doses followed by 200 mg once-weekly administration for approximately for six months
- Test Profile 1 (Figure 13-2)
  - 200 mg once-weekly administration for approximately for six months
- Test Profile 2 (Figure 13-3)
  - Three 100 mg once-daily loading doses followed by 100 mg once-weekly for approximately six months
- Test Profile 3 (Figure 13-4)
  - Three 200 mg once-daily loading doses followed by 200 mg once-weekly for approximately five and half months followed by three 200 mg once-daily doses
- Test Profile 4 (Figure 13-5)
  - Three 200 mg once-daily loading doses followed by 200 mg once-weekly for approximately five and half months followed by two 200 mg once-daily doses
- Test Profile 5 (Figure 13-6)
  - Three 400 mg once-daily loading doses
- Test Profile 6 (Figure 13-7)
  - Three 400 mg once-daily loading doses followed by 400 mg once-weekly of approximately 6 months
- Test Profile 7 (Figure 13-8)
  - Three 400 mg once-daily loading doses followed by 400 mg once-monthly for approximately 6 months

The reference profile is the dose chosen and accepted by the FDA at the end of Phase II meeting for the Phase III program. This dose level maintains trough plasma concentrations at levels > 80 ng/mL, which is believed to be the minimum concentration required to prevents breakthroughs of symptomatic malaria. Due to the potential hemolytic effect of tafenoquine in the context of G6PD screening errors, it is reasonable to contemplate eliminating the loading dose (Test Profile 1) or lowering the dose of tafenoquine (Test Profile 2) in order to determine whether alternate dosing schedule might convey the appropriate protection but improve safety.

Upon return from deployment from a malaria area (in this instance at approximately six months exposure), subjects must be given tafenoquine in such a manner that plasma concentrations remain above 80 ng/mL for at least three weeks. This is to prevent against breakthroughs from *Plasmodium falciparum* resulting from exposure to infectious mosquitoes immediately prior to the end of deployment. The simulations compare the extension of the reference profile for three weeks post deployment versus a compressed three day dosing regimen (Test Profile 3, comparison between the two in Figure 13-9) that we refer to as reverse load. The reverse load is compared to a standard 400 mg x 3 day loading dose (Test Profile 5, comparison between the two in Figure 13-10) since it was hypothesized that the maximum plasma exposure would be similar for these regimens. A composite profile where in the reference profile is compared with Test Profile 3, Test Profile 4, Test Profile 6, and Test Profile 7 is presented in Figure 13-11. There are several studies for which safety data available for the 400 mg x 3 loading dose, which are relevant for evaluating utility of the loading dose given the similarity in plasma tafenoquine levels.

For each of the dosing scenario, 5th (blue line), median (green line), and 95th (red line) percentile predicted concentrations are presented with target concentration (80 ng/mL) is identified with a horizontal line on the plot.

Simulations of reference profile at various body weights are presented in Attachment 11.

Figure 13-1 Predicted Tafenoquine Concentrations versus Time after 3 × 200 mg Once-Daily Loading Doses Followed by 200 mg Once-Weekly for Approximately 6 Months (Reference Profile)

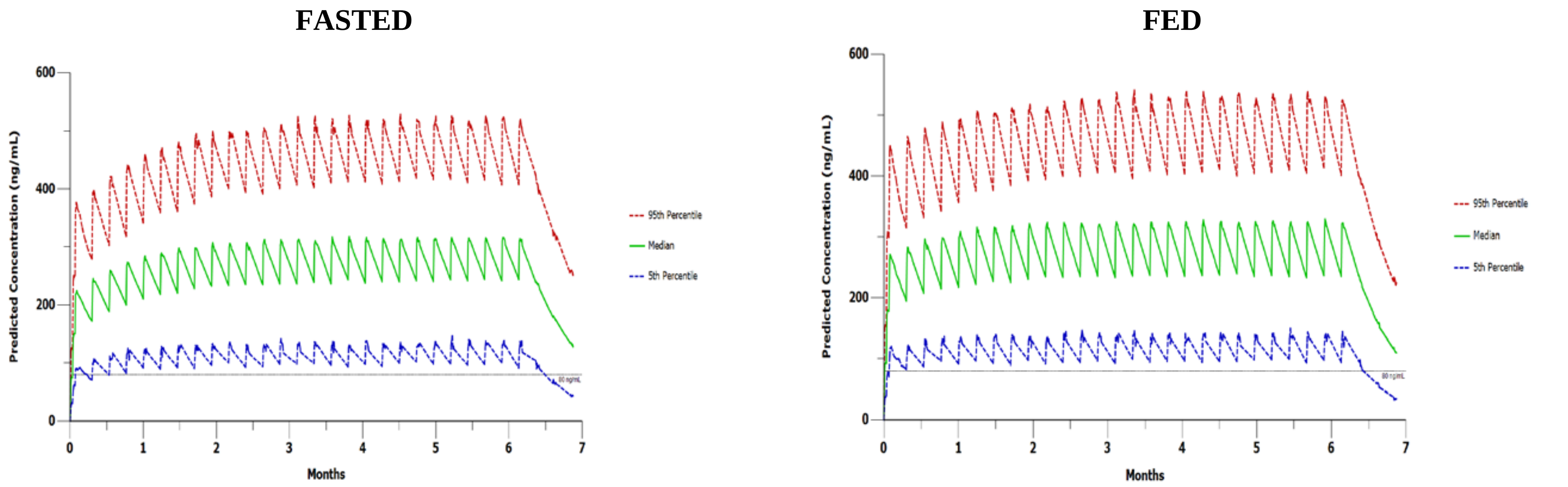

Figure 13-2 Predicted Tafenoquine Concentrations versus Time after 200 mg Once-Weekly for Approximately 6 Months (No Loading Dose) (Test Profile 1)

FASTED

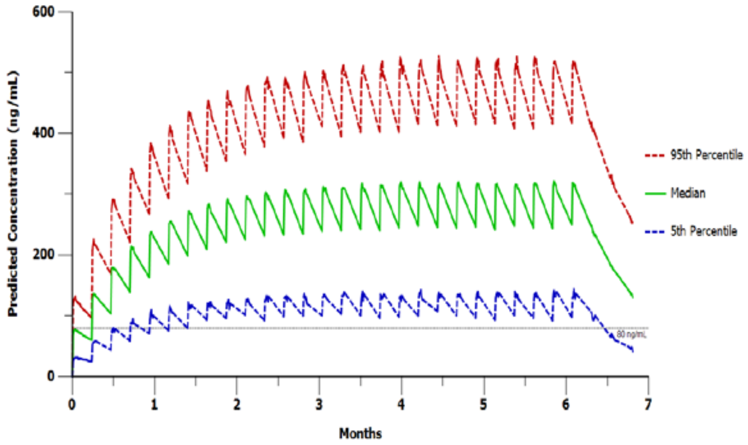

FED

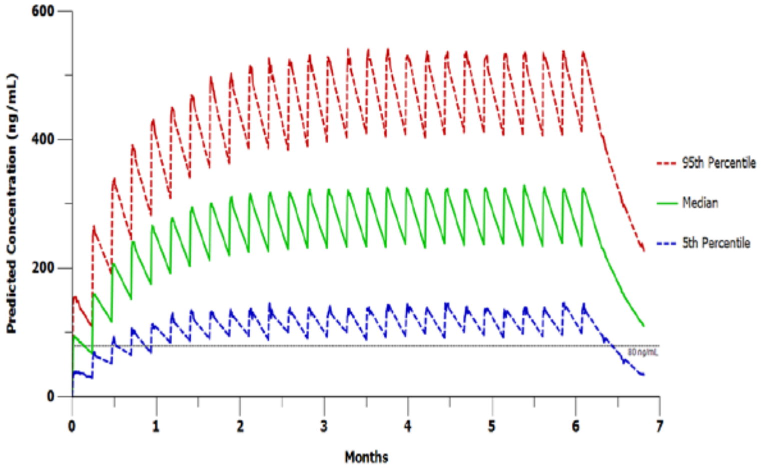

Figure 13-3 Predicted Tafenoquine Concentrations versus Time after 3 × 100 mg Once-Daily Loading Doses Followed by 100 mg Once-Weekly for Approximately 6 Months (Test Profile 2)

FASTED

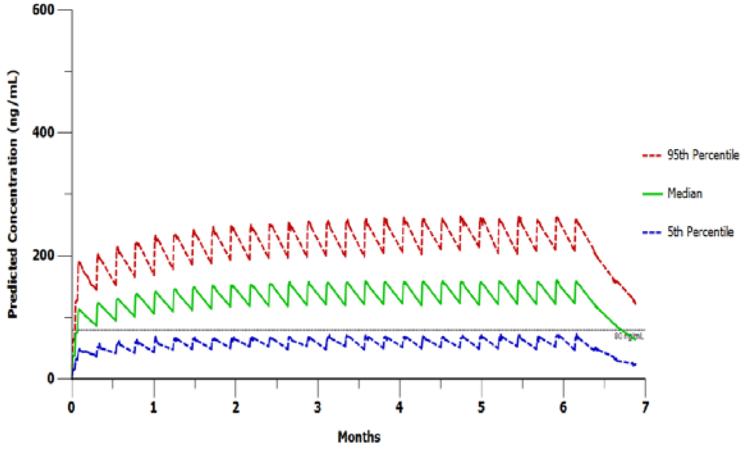

FED

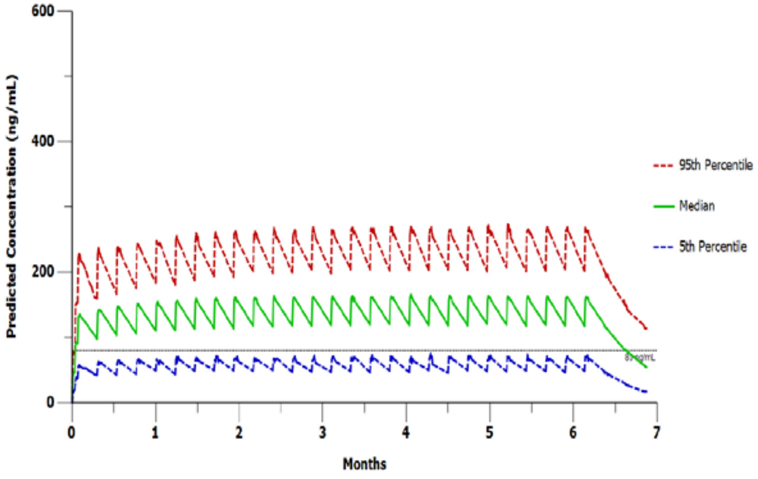

Figure 13-4 Predicted Tafenoquine Concentrations versus Time after 3 × 200 mg Once-Daily Loading Doses Followed by 200 mg Once-Weekly for Approximately 5 and Half Months followed by 3 × 200 mg Once-Daily Doses (Test Profile 3)

FASTED

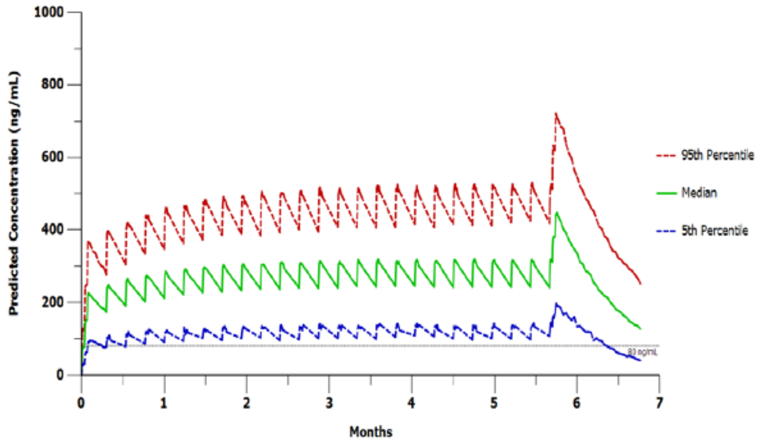

FED

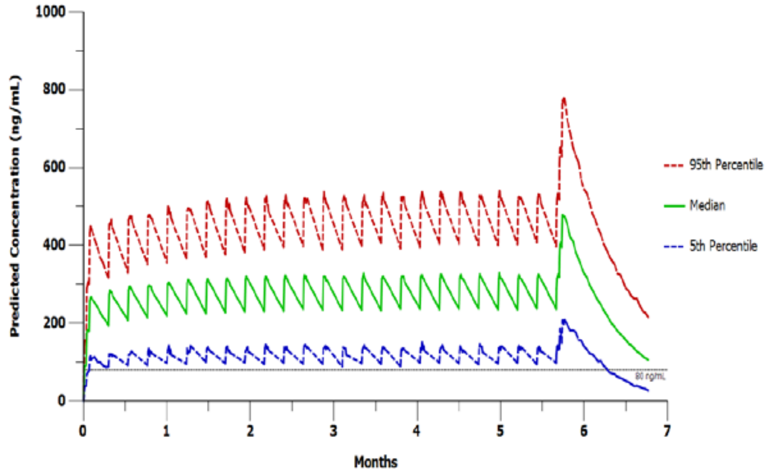

Figure 13-5 Predicted Tafenoquine Concentrations versus Time after 3 × 200 mg Once-Daily Loading Doses Followed by 200 mg Once-Weekly for Approximately 5 and Half Months followed by 2 × 200 mg Once-Daily Doses (Test Profile 4)

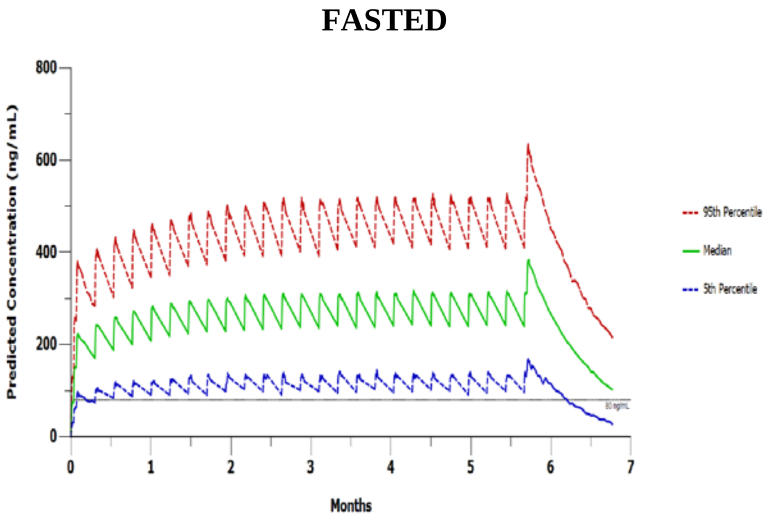

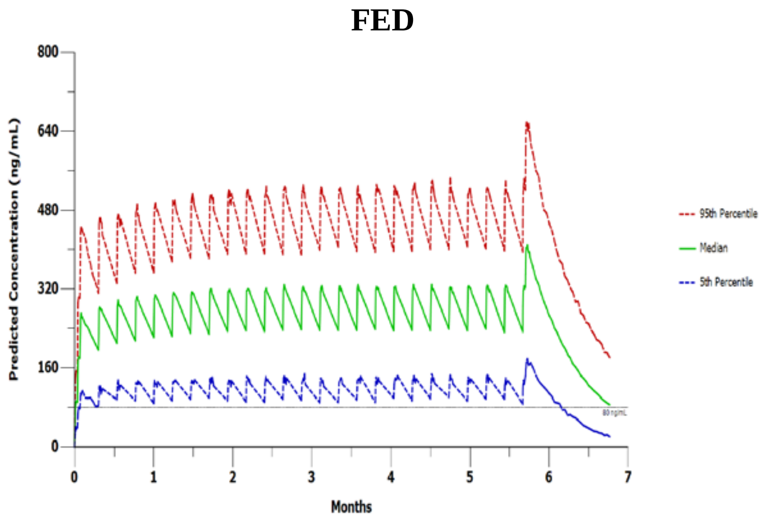

Figure 13-6 Predicted Tafenoquine Concentrations versus Time after 3 × 400 mg Once-Daily Loading Doses (Test Profile 5)

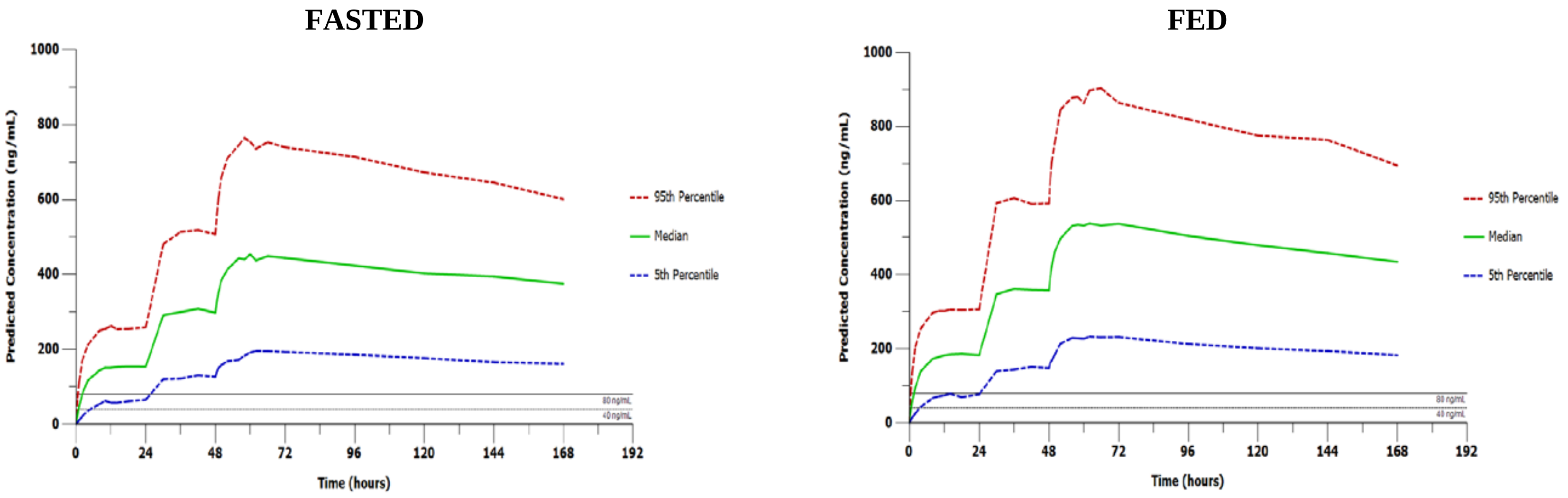

Figure 13-7 Predicted Tafenoquine Concentrations versus Time after 3 × 400 mg Once-Daily Loading Doses Followed by 400 mg Once-Weekly for Approximately 6 Months (Test Profile 6)

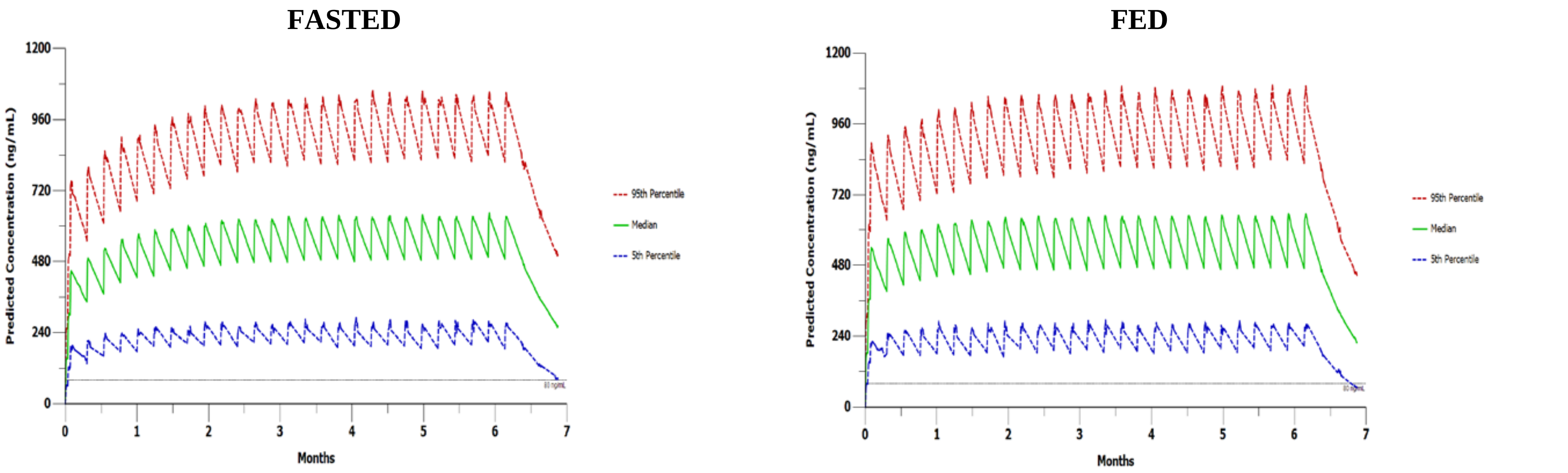

Figure 13-8 Predicted Tafenoquine Concentrations versus Time after 3 × 400 mg Once-Daily Loading Doses Followed by 400 mg Once-Monthly for Approximately 6 Months (Test Profile 7)

FASTED **FED**

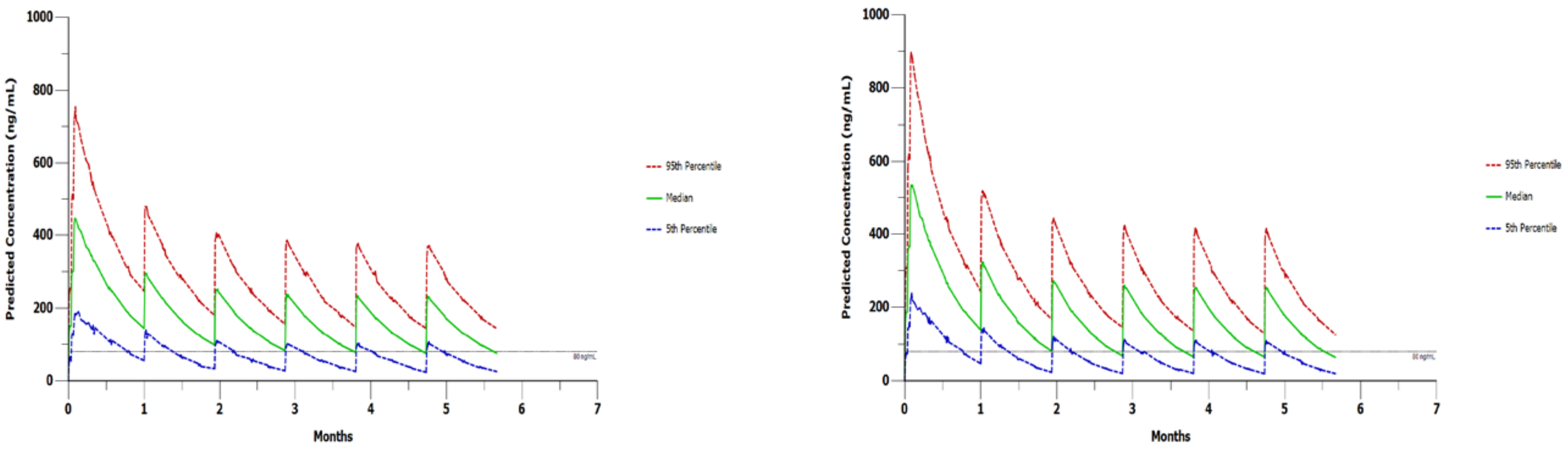

Figure 13-9 Predicted Tafenoquine Concentrations versus Time after Reference Profile versus Test Profile 3

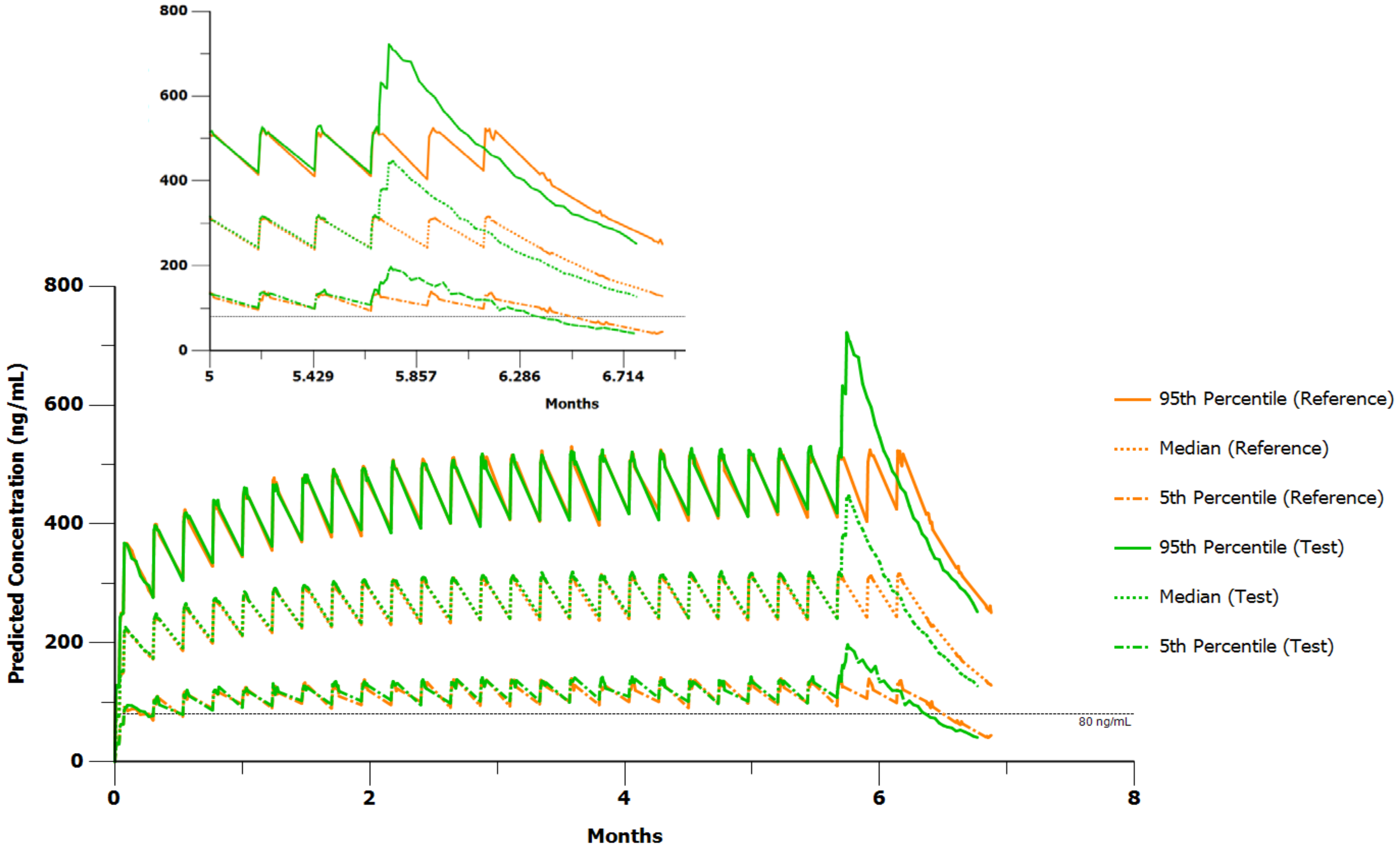

Figure 13-10 Predicted Tafenoquine Concentrations versus Time after Reference Profile versus Test Profile 5 (Fasted)

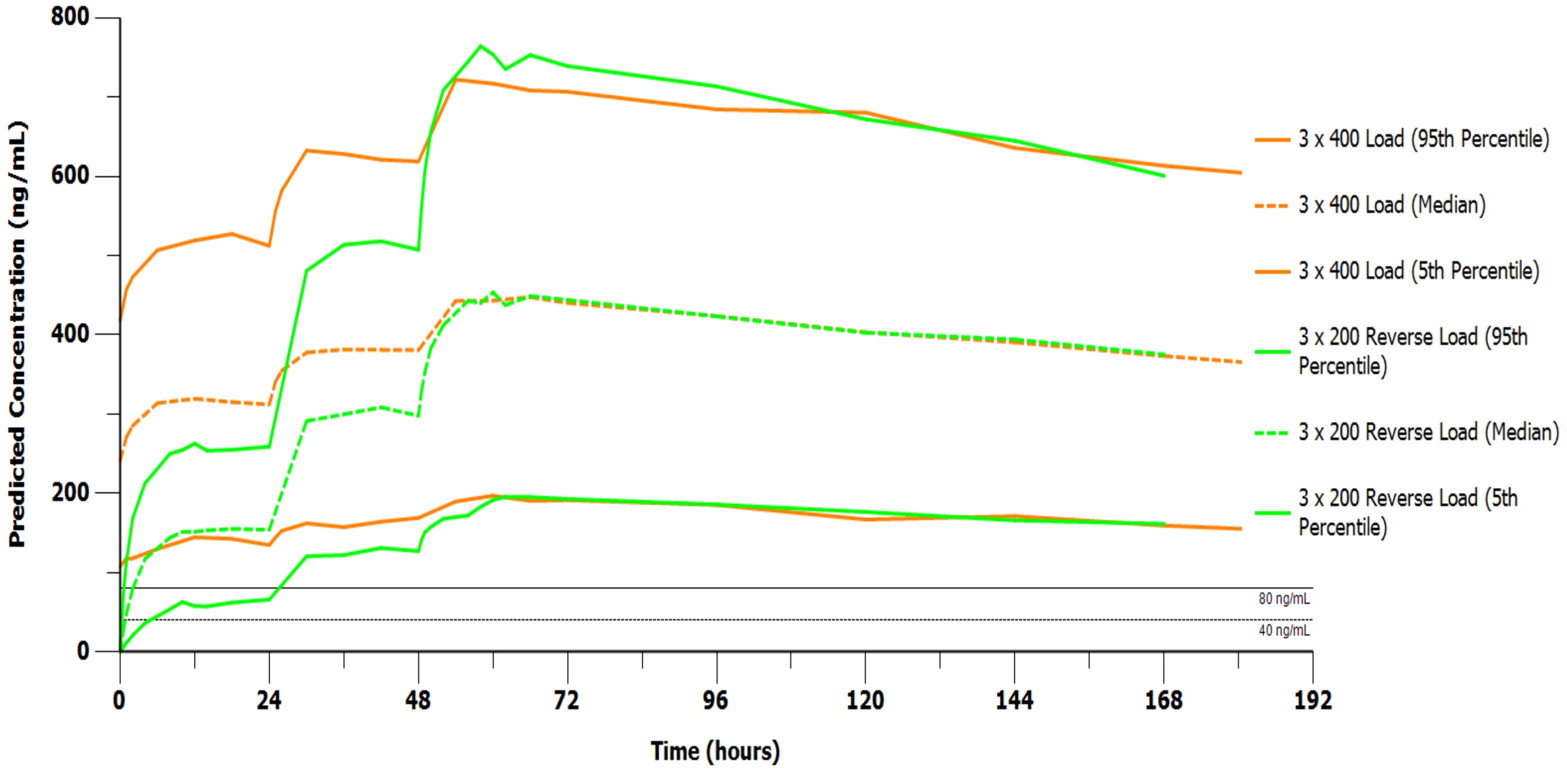

Note: For reverse load, time represents elapsed time from the start of first reverse loading dose after approximately 5 and months.

Figure 13-11 Comparison of Median Predicted Reference Profile versus Test Profiles

FASTED

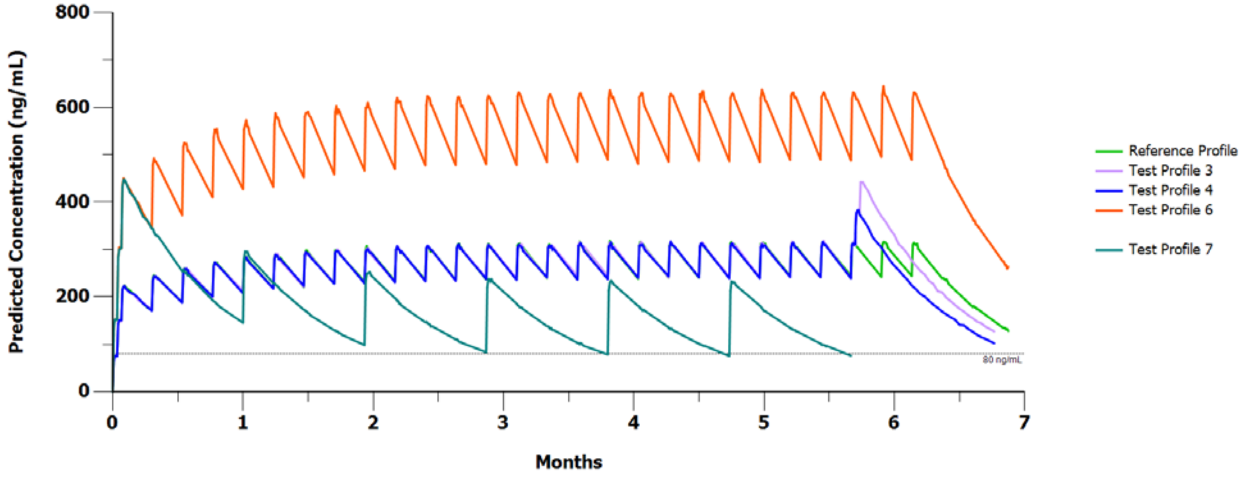

FED

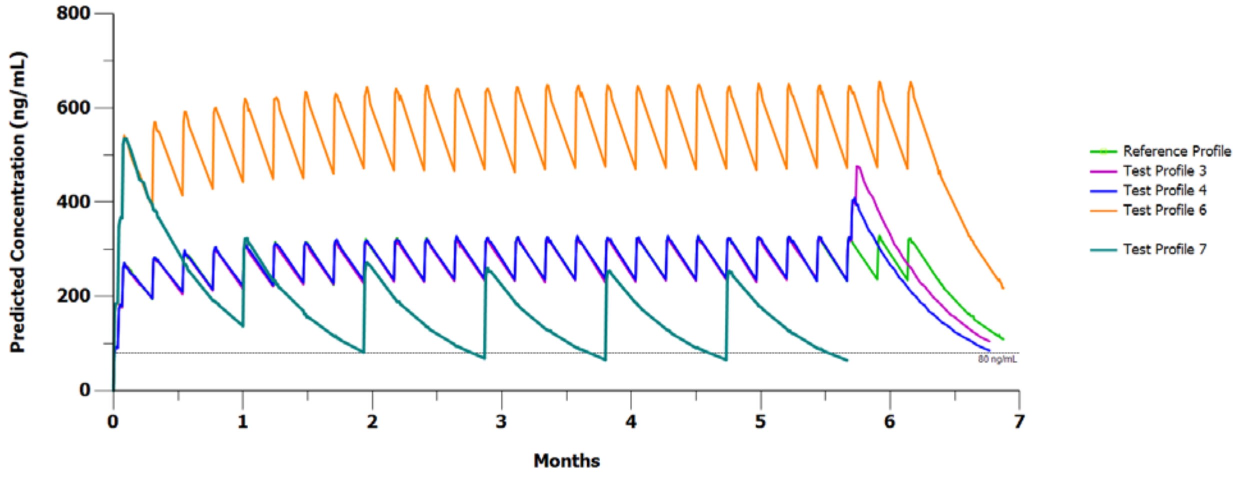

14. DISCUSSION AND CONCLUSIONS

14.1. Discussion

Population PK modeling was performed on pooled data from 10 Phase I/II/III studies.

A total of 866 subjects were included in this population PK modeling of tafenoquine. Consistent with earlier population PK analyses of tafenoquine, results showed that a one-compartment PK model with first order absorption and elimination was an appropriate base PK model for describing PK of tafenoquine administered orally. Inter-individual variability and residual variability were described by exponential models.

The final PK model revealed that apparent clearance (CL/F) of tafenoquine is a function of body weight (WT) and age. These covariates accounted for 2.9% of inter-individual variability of CL/F (i.e., decrease inter-individual variability from 26.5% to 23.6%. The mean estimate of CL/F for an individual with a median age of 25 years in the lowest body weight (43 kg) could be as low as 0.97-fold of the average CL/F. The mean estimate of CL/F in the highest body weight (135 kg) could be as large as 1.03-fold of the average CL/F. The mean estimate of CL/F for an individual with a median body weight of 75 kilograms in the younger age category (18 years) could be 1.07-fold of the average CL/F. The mean estimate of CL/F in the older age category (60 years) could be 0.84-fold of the average CL/F.

The final PK model revealed that apparent volume of distribution (V/F) of tafenoquine is a function of body weight (WT) and food. These covariates accounted for 5.5% of inter-individual variability of V/F (i.e., decrease inter-individual variability from 29.6% to 24.1%). The mean estimate of V/F for a fasted individual in the lowest body weight (43 kg) could be as low as 0.65-fold of the average V/F. The mean estimate of V/F in the highest body weight (135 kg) could be as large as 1.58-fold of the average V/F. For an individual with a median body weight of 75 kilograms, V/F of tafenoquine under fed conditions is 17.8% lower than the typical individual (2030 L vs. 2470 L).

Mean bootstrap estimates of all PK parameters were compared to those from the final PK model. The differences of mean bootstrap estimates from the NONMEM estimates of those parameters were less than 5%, demonstrating a satisfactory level of reliability of the final PK model. Posterior visual predictive checks were performed on the final PK model. These were judged to adequately reproduce drug concentrations.

Current population PK modeling of pooled data from 10 studies demonstrated consistent results with the previous independent population PK modeling for data from Study 33 and Study 44. There are some differences with the previous reports. The apparent V/F from pooled population PK modeling was 2470 L compared to 1820 L and 1110 L reported by Edstein et al.[1] and Charles et al.[2], respectively. The systemic CL/F was 4.17 L/hr compared to 3.20 L/hr and 3.02 L/hr from Edstein et al.[1] and Charles et al.[2], respectively. In earlier attempts, only body weight was identified as the significant covariate for CL/F and V/F. However, in the current analysis, not only body weight was significant for CL/F and V/F, age and FOOD were also found to be significant covariates for CL/F and V/F, respectively.

Derived elimination half-life of 17 hours was similar to the one reported by Edstein et al.[1] (16 hours) and both were different from 11 hours reported by Charles et al.[2].

14.2. Conclusions

- Pharmacokinetics of oral tafenoquine follows a one-compartment model with first order absorption and elimination
- Body weight and age were significant covariates with respect to CL/F of tafenoquine and CL/F was found to increase with increasing body weight and decreased with age
- Body weight and food were significant covariates with respect to V/F of tafenoquine and V/F was found to increase with increasing body weight and decreased with food

15. REFERENCES

3. Investigator Brochure Ver. 09, 18 November 2011.
4. PDx-Pop Manual
5. Ette EI, Williams PJ, Kim YH, Lane JR, Liu M, Capparelli EV. Model Appropriateness and Population Pharmacokinetic Modeling. *J. Clin. Pharmacol.* 43: 610-623, 2003.
6. Pharmacokinetic-Pharmacodynamic Modeling and Simulation. Bonate PL. Publisher: *Springer*, Pg 296-298, 2006.
7. Guidance for Industry, Population Pharmacokinetics, FDA, February 1999.

16. END-OF-TEXT TABLES AND FIGURES

End-of-Text Table 16-1 Data Item Definitions for NONMEM PK Input File

Variable	Data Definition
C	Reserved blank column
ID	Patient/subject identification number
SID	Sequential subject identification number
TIME	Elapsed time from first dose
EVID	Event Identification<br>• EVID = 0 for blood sampling event/observation event<br>• EVID = 1 for dosing event<br>• EVID = 2 for observation excluded from analysis [BLQ (0) and when concentration data are missing]
MDV	Missing Dependent variable<br>• MDV = 0 for observation<br>• the DV item is an observed value, i.e., DV is not missing<br>• MDV = 1 for missing dependent variable<br>• The DV data item is not regarded an observed value, i.e., DV is missing.<br>• EVID values 1, 2, 3, 4 must all have MDV=1
AMT	Amount of tafenoquine dose (mg)<br>• Dose amount (mg) for EVID = 1<br>• Set AMT to 0 for EVID for EVID = 0 and 2
DV	Dependent variable<br>• Tafenoquine concentration (ng/mL)
LNDV	Log-transformed DV<br>• Natural log transform concentration values
ASTDY	ARMY Study identification<br>• ASTDY = 1 for Study 1<br>• ASTDY = 2 for Study 2<br>• ASTDY = 3 for Study 3<br>• ASTDY = 4 for Study 4<br>• ASTDY = 5 for Study 5<br>• ASTDY = 14 for Study 14<br>• ASTDY = 15 for Study 15<br>• ASTDY = 33 for Study 33<br>• ASTDY = 44 for Study 44<br>• ASTDY = 58 for Study 58
GSTDY	GSK Study identification<br>• GSTDY = 50 for AStudy 1<br>• GSTDY = 52 for AStudy 2<br>• GSTDY = 53 for AStudy 3<br>• GSTDY = 51 for AStudy 4<br>• GSTDY = 54 for AStudy 5<br>• GSTDY = 14 for AStudy 14<br>• GSTDY = 15 for AStudy 15<br>• GSTDY = 33 for AStudy 33<br>• GSTDY = 44 for AStudy 44<br>• GSTDY = 58 for AStudy 58

REG	DOSE REGIMEN<br>• REG = 1 for ASTDY = 1, 2, 3<br>• REG = 2 for ASTDY = 4<br>• REG = 3 for STDY = 5<br>• 600 mg QD x 2 days / 300 mg QW<br>• REG = 4 for ASTDY = 14<br>• 400 mg QD x 3 days<br>• REG = 5 for ASTDY = 15<br>• 400 mg QD x 3 days<br>• REG = 6 for ASTDY = 33<br>• 200 mg QD x 3 days / 200 mg QW<br>• REG = 7 for ASTDY = 44<br>• 400 mg QD x 3 days / 400 mg QM<br>• REG = 8 for ASTDY = 44<br>• 400 mg QD x 3 days / 400 mg QW<br>• REG = 9 for ASTDY = 58<br>• 400 mg QD x 3 days
AGE	• Age in years
WT	• Weight in kilograms
SEX	• SEX = 0 for Male<br>• SEX = 1 for Female
RACE	• RACE = 1 for Asian or Oriental<br>• RACE = 2 for Black or African American<br>• RACE = 3 for Caucasian/White<br>• RACE = 4 for Hispanic<br>• RACE = 5 for Other
ASSY	ASSAY FLAG<br>• ASSY = 1<br>• ASSY = 2<br>• ASSY = 3 for missing
CLCR	Creatinine clearance<br>1. Serum creatinine units (mg/dL)<br>Males:<br>Females: $CRCL(mL/\min) = \frac{(140 - AGE) \times Wt\,in\,kg}{72 \times Serum Creatinine (mg/dL)}$<br>$CRCL(mL/\min) = \frac{(140 - AGE) \times Wt\,in\,kg \times 0.85}{72 \times Serum\ Creatinine\ (mg/dL)}$<br>2. Serum creatinine units (umol/L)<br>Males: $CRCL\,(mL/min) = 1.23 \times \frac{(140 - AGE) \times Wt(kg)}{Serum\,Creatinine\,(umol/L)}$<br>Females:<br>$CRCL\,(mL/min) = 1.04 \times \frac{(140 - AGE) \times Wt(kg)}{Serum\,Creatinine\,(umol/L)}$
HCRIT	Hematocrit (%)
MAL	Malaria status<br>• MAL = 0 for No<br>• MAL = 1 for Yes

SMOK	Smoking status<br>• SMOK = 0 for No<br>• SMOK = 1 for Yes
FOOD	Fed or Fasted<br>• FOOD = 0 for No<br>• FOOD = 1 for Yes
STAK	Finger Prick Smear<br>• STAK = 0 for No<br>• STAK = 1 for Yes
PAR	Parasitemeia<br>• PAR = 0 for No<br>• PAR = 1 for Yes
LAST	Last Attack of Malaria<br>• LAST = 0 for No<br>• LAST = 1 for Yes
SPEC	Species of parasitaemia<br>• SPEC = 1000 for P.Falciparum<br>• SPEC = 200 for P.Malariae<br>• SPEC = 30 for P.Ovale<br>• SPEC = 4 for P.Vivax
METH	Methhaemoglobin (%)

NONMEM Data File Name: USTAFCMB10OCT12.csv

End-of-Text Table 16-2 Population PK Parameters of Base PK Model

Parameters	Description	Estimate	RSE%
θ1	CL/F (L/hr)	4.10	1.02
θ2	V/F (L)	2050	1.20
θ3	Ka (1/hr)	0.356	4.38
ω11	Variance of inter-individual variability (ETA) in CL/F	0.0703	6.66
ω21	Covariance of inter-individual variability (ETA) in CL/F and inter-individual variability (ETA) in V/F	0.0472	9.19
ω22	Variance of inter-individual variability (ETA) in V/F	0.0879	7.47
ω33	Variance of inter-individual variability (ETA) in Ka	0.294	14.8
σ11	Variance of proportional residual error (EPSILON)	0.0487	6.10

Source: USARMYTAFPKB0218.sum, Attachment 4.2

End-of-Text Table 16-3 Population PK Parameters of Final PK Model

Parameters	Description	Estimate	RSE%
θ1	CL/F (L/hr)	4.17	0.935
θ2	V/F (L)	2470	2.99
θ3	Ka (1/hr)	0.359	4.37
θ4	WT/75 on CL/F	0.552	8.39
θ5	WT/75 on CL/F	0.781	7.84
θ6	AGE/25 on CL/F	-0.200	15.9
θ7	FOOD on V/F	0.822	3.26
ω11	Variance of inter-individual variability (ETA) in CL/F	0.0555	7.44
ω21	Covariance of inter-individual variability (ETA) in CL/F and inter-individual variability (ETA) in V/F	0.0289	12.1
ω22	Variance of inter-individual variability (ETA) in V/F	0.0583	7.98
ω33	Variance of inter-individual variability (ETA) in Ka	0.293	15.0
σ11	Variance of proportional residual error (EPSILON)	0.0488	6.07

Source: USARMYTAFPKC0409.sum, Attachment 8.2

End-of-Text Table 16-4 Demographic Summary of Subjects/Patients Used in the Population PK Analysis - Tafenoquine Population PK Modeling -Overall

Baseline Characteristic	Statistic	All Subjects	Male	Female
Number of Subjects	n (%)	866	808 (93.3%)	58 (6.7%)
Age (years)	Mean	27.8	27.1	37.2
	SE	0.28	0.25	1.68
	Median	25.0	25.0	35.0
	Min,Max	18.0,60.0	18.0,60.0	19.0,60.0
Race				
Asian or Oriental	n (%)	181 (20.9%)	172 (19.9%)	9 (1.0%)
Black or African	n (%)	26 (3.0%)	21 (2.4%)	5 (0.6%)
Caucasian/White	n (%)	626 (72.3%)	582 (67.2%)	44 (5.1%)
Hispanic	n (%)	31 (3.6%)	31 (3.6%)	-
Other	n (%)	2 (0.2%)	2 (0.2%)	-
Food				
No	n (%)	92 (10.6%)	86 (9.9%)	6 (0.7%)
Yes	n (%)	774 (89.4%)	722 (83.4%)	52 (6.0%)
Weight (kg)	Mean	75.0	75.9	62.4
	SE	0.47	0.48	1.37
	Median	75.0	76.0	62.3
	Min,Max	43.0,135.0	43.0,135.0	43.0,88.0

End-of-Text Table 16-5 Demographic Summary of Subjects/Patients Used in the Population PK Analysis – Army Study No. 1

Baseline Characteristic	Statistic	All Subjects	Male	Female
Number of Subjects	n (%)	45	45 (100.0%)	-
Age (years)	Mean	27.8	27.8	-
	SE	0.70	0.70	-
	Median	28.0	28.0	-
	Min,Max	18.0,35.0	18.0,35.0	-
Race				
Black or African	n (%)	11 (24.4%)	11 (24.4%)	-
Caucasian/White	n (%)	18 (40.0%)	18 (40.0%)	-
Hispanic	n (%)	16 (35.6%)	16 (35.6%)	-
Food				
No	n (%)	45 (100.0%)	45 (100.0%)	-
Weight (kg)	Mean	76.7	76.7	-
	SE	1.21	1.21	-
	Median	79.0	79.0	-
	Min,Max	60.0,90.0	60.0,90.0	-

End-of-Text Table 16-6 Demographic Summary of Subjects/Patients Used in the Population PK Analysis – Army Study No. 2

Baseline Characteristic	Statistic	All Subjects	Male	Female
Number of Subjects	n (%)	18	18 (100.0%)	-
Age (years)	Mean	22.6	22.6	-
	SE	0.82	0.82	-
	Median	23.0	23.0	-
	Min,Max	18.0,32.0	18.0,32.0	-
Race				
Black or African	n (%)	2 (11.1%)	2 (11.1%)	-
Caucasian/White	n (%)	16 (88.9%)	16 (88.9%)	-
Food				
No	n (%)	18 (100.0%)	18 (100.0%)	-
Weight (kg)	Mean	76.3	76.3	-
	SE	2.63	2.63	-
	Median	77.0	77.0	-
	Min,Max	55.0,95.0	55.0,95.0	-

End-of-Text Table 16-7 Demographic Summary of Subjects/Patients Used in the Population PK Analysis – Army Study No. 3

Baseline Characteristic	Statistic	All Subjects	Male	Female
Number of Subjects	n (%)	4	2 (50.0%)	2 (50.0%)
Age (years)	Mean	26.3	23.0	29.5
	SE	2.06	2.00	0.50
	Median	27.0	23.0	29.5
	Min,Max	21.0,30.0	21.0,25.0	29.0,30.0
Race				
Caucasian/White	n (%)	4 (100.0%)	2 (50.0%)	2 (50.0%)
Food				
No	n (%)	4 (100.0%)	2 (50.0%)	2 (50.0%)
Weight (kg)	Mean	69.3	72.0	66.5
	SE	2.59	5.00	0.50
	Median	67.0	72.0	66.5
	Min,Max	66.0,77.0	67.0,77.0	66.0,67.0

End-of-Text Table 16-8 Demographic Summary of Subjects/Patients Used in the Population PK Analysis – Army Study No. 4

Baseline Characteristic	Statistic	All Subjects	Male	Female
Number of Subjects	n (%)	25	21 (84.0%)	4 (16.0%)
Age (years)	Mean	32.4	32.3	33.3
	SE	1.24	1.44	1.89
	Median	33.0	33.0	33.0
	Min,Max	23.0,45.0	23.0,45.0	29.0,38.0
Race				
Black or African	n (%)	9 (36.0%)	5 (20.0%)	4 (16.0%)
Caucasian/White	n (%)	5 (20.0%)	5 (20.0%)	-
Hispanic	n (%)	11 (44.0%)	11 (44.0%)	-
Food				
No	n (%)	25 (100.0%)	21 (84.0%)	4 (16.0%)
Weight (kg)	Mean	73.8	74.3	71.5
	SE	2.06	2.20	6.36
	Median	75.0	75.0	68.5
	Min,Max	60.0,90.0	60.0,90.0	61.0,88.0

End-of-Text Table 16-9 Demographic Summary of Subjects/Patients Used in the Population PK Analysis – Army Study No. 5

Baseline Characteristic	Statistic	All Subjects	Male	Female
Number of Subjects	n (%)	10	10 (100.0%)	-
Age (years)	Mean	23.6	23.6	-
	SE	1.56	1.56	-
	Median	22.5	22.5	-
	Min,Max	19.0,36.0	19.0,36.0	-
Race				
Caucasian/White	n (%)	8 (80.0%)	8 (80.0%)	-
Other	n (%)	2 (20.0%)	2 (20.0%)	-
Food				
Yes	n (%)	10 (100.0%)	10 (100.0%)	-
Weight (kg)	Mean	79.0	79.0	-
	SE	4.54	4.54	-
	Median	75.0	75.0	-
	Min,Max	61.0,109.0	61.0,109.0	-

End-of-Text Table 16-10 Demographic Summary of Subjects/Patients Used in the Population PK Analysis – Army Study No. 14

Baseline Characteristic	Statistic	All Subjects	Male	Female
Number of Subjects	n (%)	58	43 (74.1%)	15 (25.9%)
Age (years)	Mean	39.7	36.9	47.7
	SE	1.49	1.75	1.61
	Median	40.0	36.0	48.0
	Min,Max	20.0,60.0	20.0,60.0	35.0,57.0
Race				
Caucasian/White	n (%)	58 (100.0%)	43 (74.1%)	15 (25.9%)
Food				
Yes	n (%)	58 (100.0%)	43 (74.1%)	15 (25.9%)
Weight (kg)	Mean	74.0	77.6	63.6
	SE	1.35	1.32	1.82
	Median	72.5	77.9	64.2
	Min,Max	51.7,94.8	62.0,94.8	51.7,71.7

End-of-Text Table 16-11 Demographic Summary of Subjects/Patients Used in the Population PK Analysis – Army Study No. 15

Baseline Characteristic	Statistic	All Subjects	Male	Female
Number of Subjects	n (%)	34	20 (58.8%)	14 (41.2%)
Age (years)	Mean	42.4	38.4	48.1
	SE	1.80	1.97	2.78
	Median	41.5	39.5	47.0
	Min,Max	25.0,60.0	25.0,59.0	29.0,60.0
Race				
Caucasian/White	n (%)	34 (100.0%)	20 (58.8%)	14 (41.2%)
Food				
Yes	n (%)	34 (100.0%)	20 (58.8%)	14 (41.2%)
Weight (kg)	Mean	75.4	81.7	66.4
	SE	2.02	1.36	3.28
	Median	79.5	81.8	63.5
	Min,Max	52.0,91.0	71.0,91.0	52.0,87.0

End-of-Text Table 16-12 Demographic Summary of Subjects/Patients Used in the Population PK Analysis – Army Study No. 33

Baseline Characteristic	Statistic	All Subjects	Male	Female
Number of Subjects	n (%)	491	477 (97.1%)	14 (2.9%)
Age (years)	Mean	25.5	25.5	25.1
	SE	0.24	0.24	1.22
	Median	24.0	24.0	24.5
	Min,Max	18.0,47.0	18.0,47.0	19.0,34.0
Race				
Black or African	n (%)	4 (0.8%)	3 (0.6%)	1 (0.2%)
Caucasian/White	n (%)	483 (98.4%)	470 (95.7%)	13 (2.6%)
Hispanic	n (%)	4 (0.8%)	4 (0.8%)	-
Food				
Yes	n (%)	491 (100.0%)	477 (97.1%)	14 (2.9%)
Weight (kg)	Mean	81.0	81.5	62.8
	SE	0.54	0.53	1.83
	Median	80.0	80.0	64.0
	Min,Max	50.0,135.0	50.0,135.0	51.0,75.0

End-of-Text Table 16-13 Demographic Summary of Subjects/Patients Used in the Population PK Analysis – Army Study No. 44

Baseline Characteristic	Statistic	All Subjects	Male	Female
Number of Subjects	n (%)	135	135 (100.0%)	-
Age (years)	Mean	28.6	28.6	-
	SE	0.67	0.67	-
	Median	23.0	23.0	-
	Min,Max	21.0,46.0	21.0,46.0	-
Race				
Asian or Oriental	n (%)	135 (100.0%)	135 (100.0%)	-
Food				
Yes	n (%)	135 (100.0%)	135 (100.0%)	-
Weight (kg)	Mean	60.5	60.5	-
	SE	0.60	0.60	-
	Median	60.0	60.0	-
	Min,Max	45.0,90.0	45.0,90.0	-

End-of-Text Table 16-14 Demographic Summary of Subjects/Patients Used in the Population PK Analysis – Army Study No. 58

Baseline Characteristic	Statistic	All Subjects	Male	Female
Number of Subjects	n (%)	46	37 (80.4%)	9 (19.6%)
Age (years)	Mean	25.5	25.5	25.3
	SE	0.94	1.06	2.12
	Median	23.5	23.0	25.0
	Min,Max	20.0,43.0	20.0,43.0	20.0,40.0
Race				
Asian or Oriental	n (%)	46 (100.0%)	37 (80.4%)	9 (19.6%)
Food				
Yes	n (%)	46 (100.0%)	37 (80.4%)	9 (19.6%)
Weight (kg)	Mean	52.9	53.9	48.8
	SE	0.88	0.95	1.70
	Median	52.0	53.0	48.0
	Min,Max	43.0,69.0	43.0,69.0	43.0,59.0

End-of-Text Figure 16-1 Plot of Observed (DV) vs. Individual Predicted (IPRED) Tafenoquine Concentrations (Final PK Model)

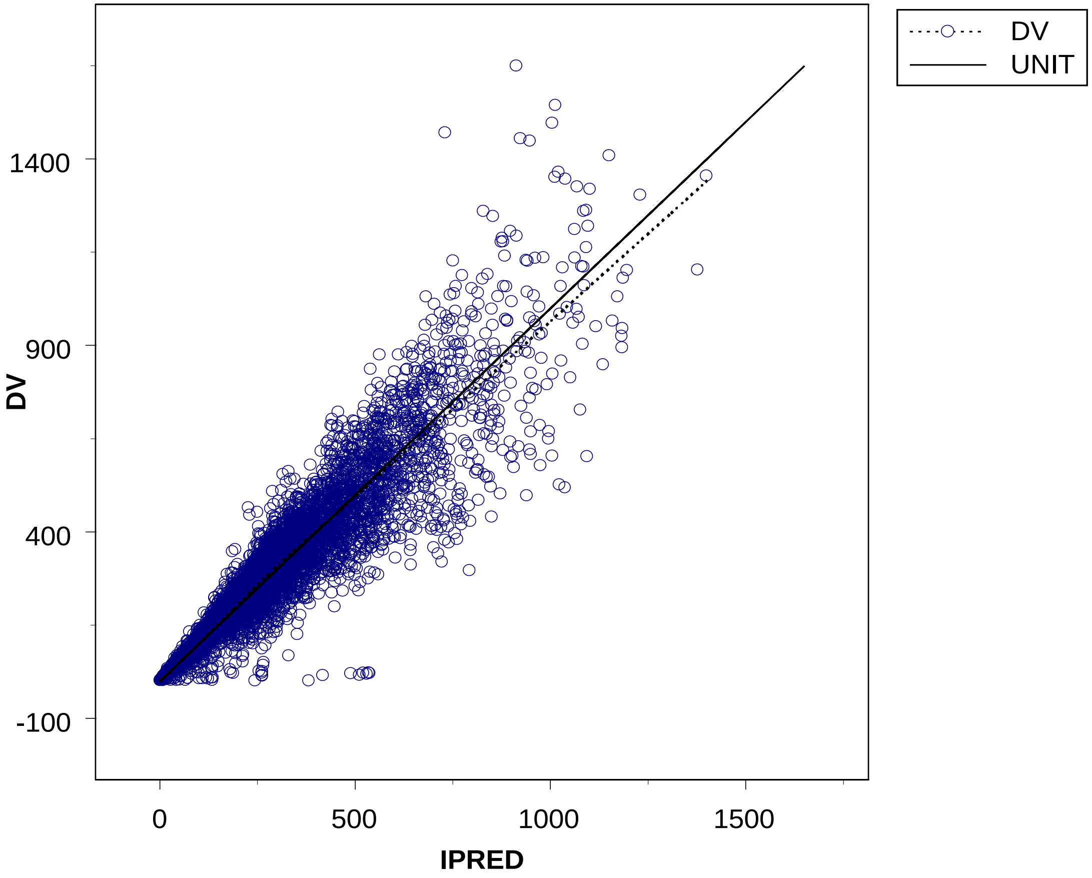

USARMYTAFPKC0409

Oct. 21, 2012 9:37:05 PM

End-of-Text Figure 16-2 Plot of Observed (DV) vs. Population Predicted (PRED) Tafenoquine Concentrations (Final PK Model)

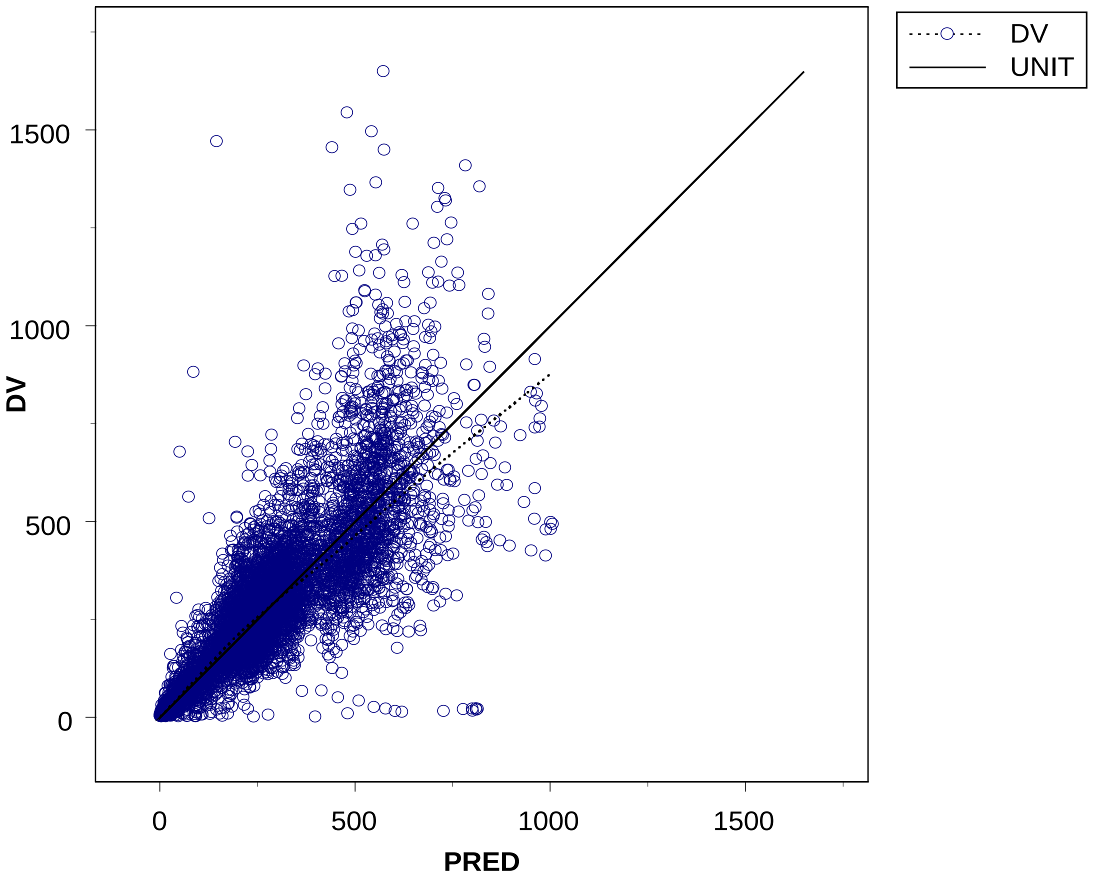

USARMYTAFPKC0409

Oct. 21, 2012 9:36:49 PM

End-of-Text Figure 16-3 Plot of Residuals (RES) vs. Population Predicted (PRED) Tafenoquine Concentrations (Final PK Model)

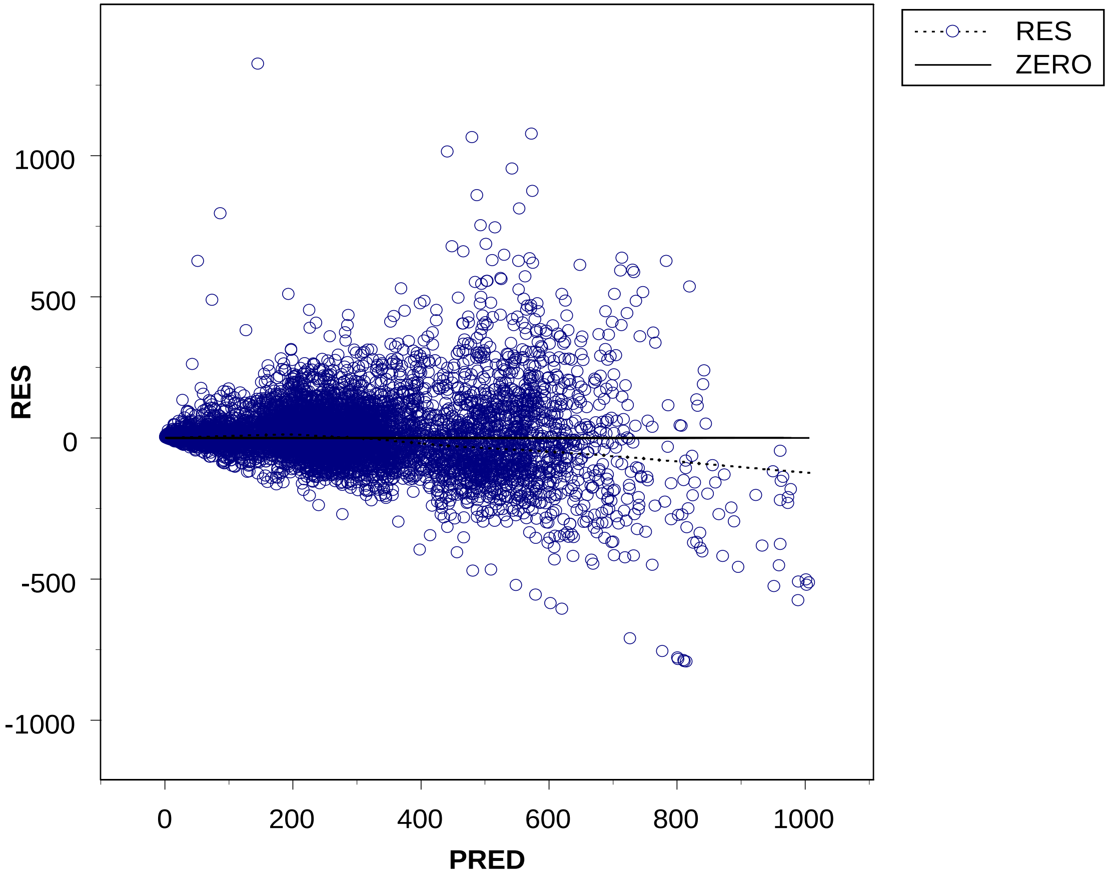

USARMYTAFPKC0409

Oct. 21, 2012 9:37:20 PM

End-of-Text Figure 16-4 Plot of Residuals (RES) vs. Time (Final PK Model)

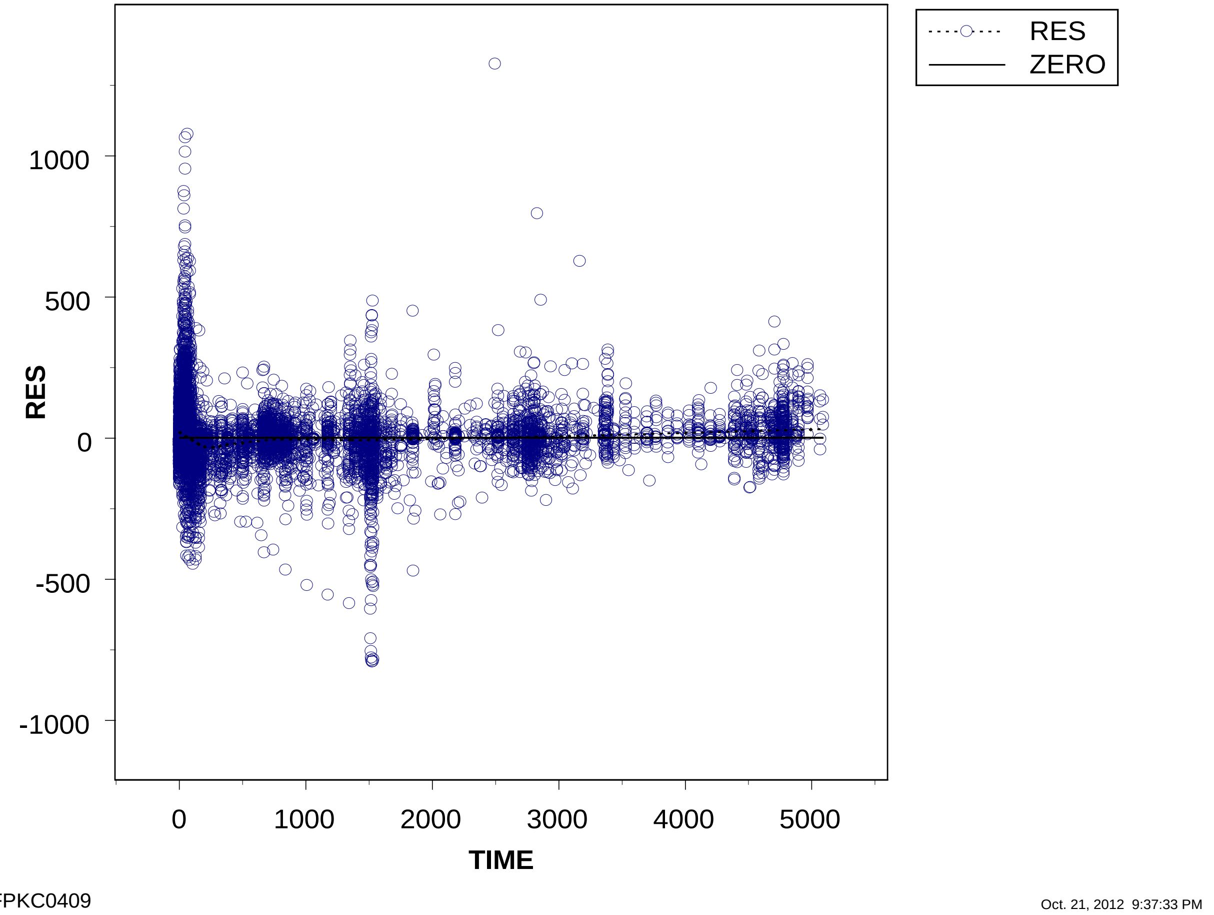

Note: Time in hours

End-of-Text Figure 16-5 Plot of Residuals (RES) vs. Time after Dose (Final PK Model)

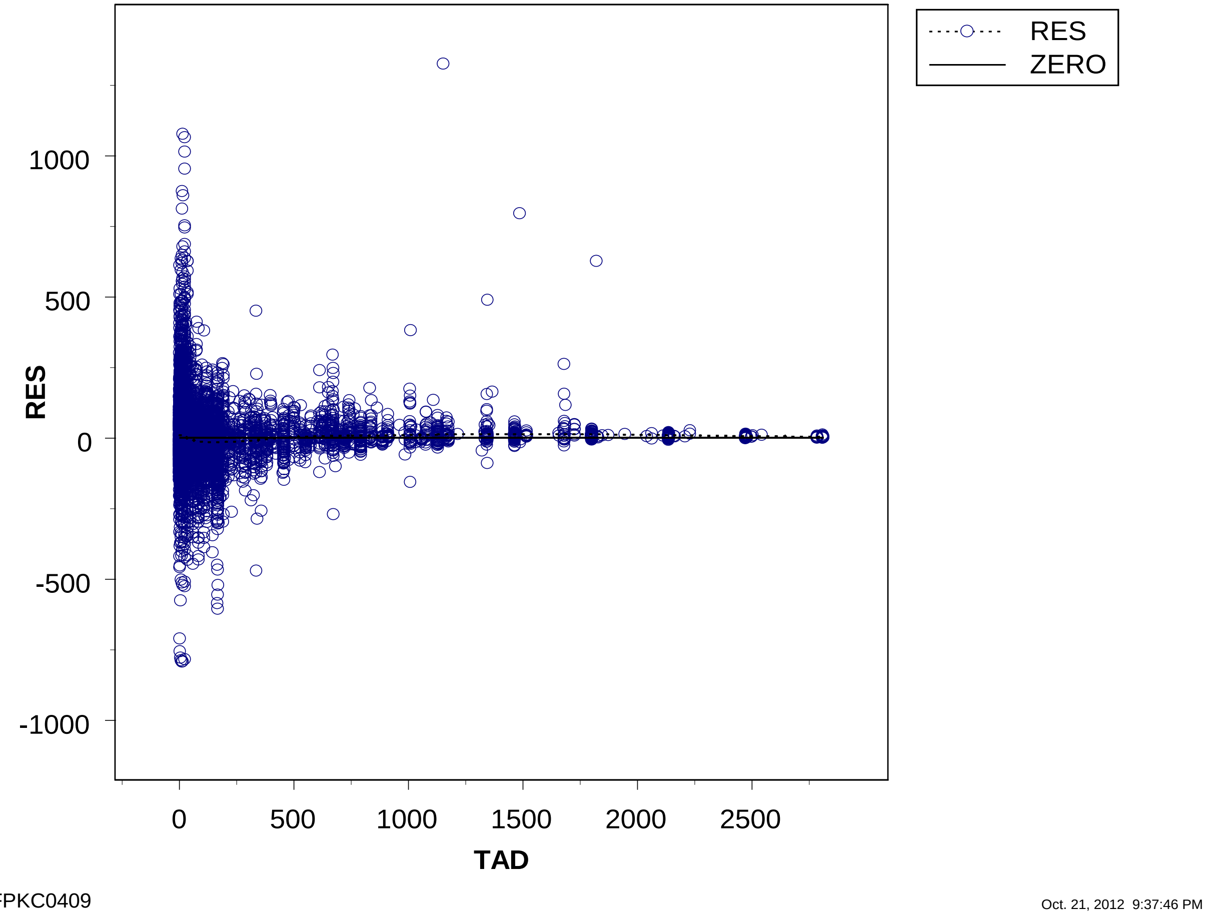

TAD: Time after dose in hours

End-of-Text Figure 16-6 Plot of Weighted Residuals (WRES) vs. Population Predicted (PRED) Tafenoquine Concentrations (Final PK Model)

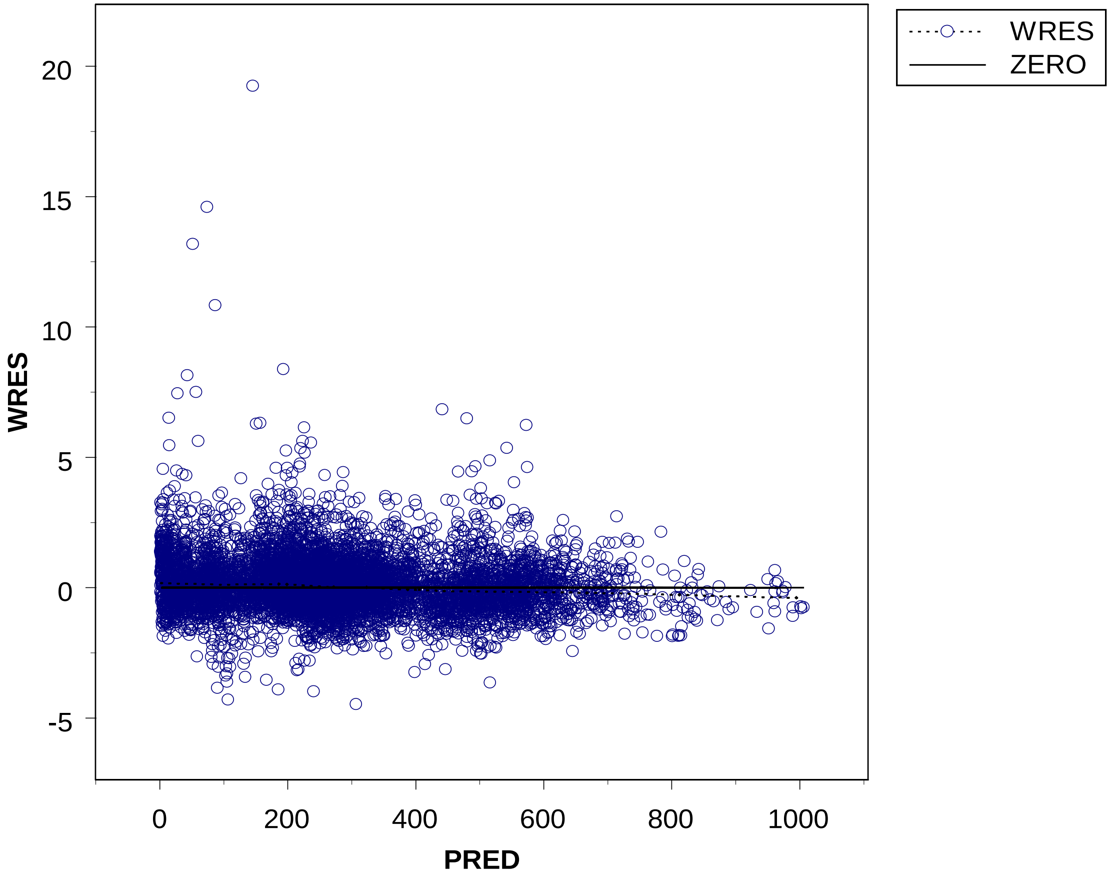

USARMYTAFPKC0409

Oct. 21, 2012 9:37:58 PM

End-of-Text Figure 16-7 Plot of Weighted Residuals (WRES) vs. Time (Final PK Model)

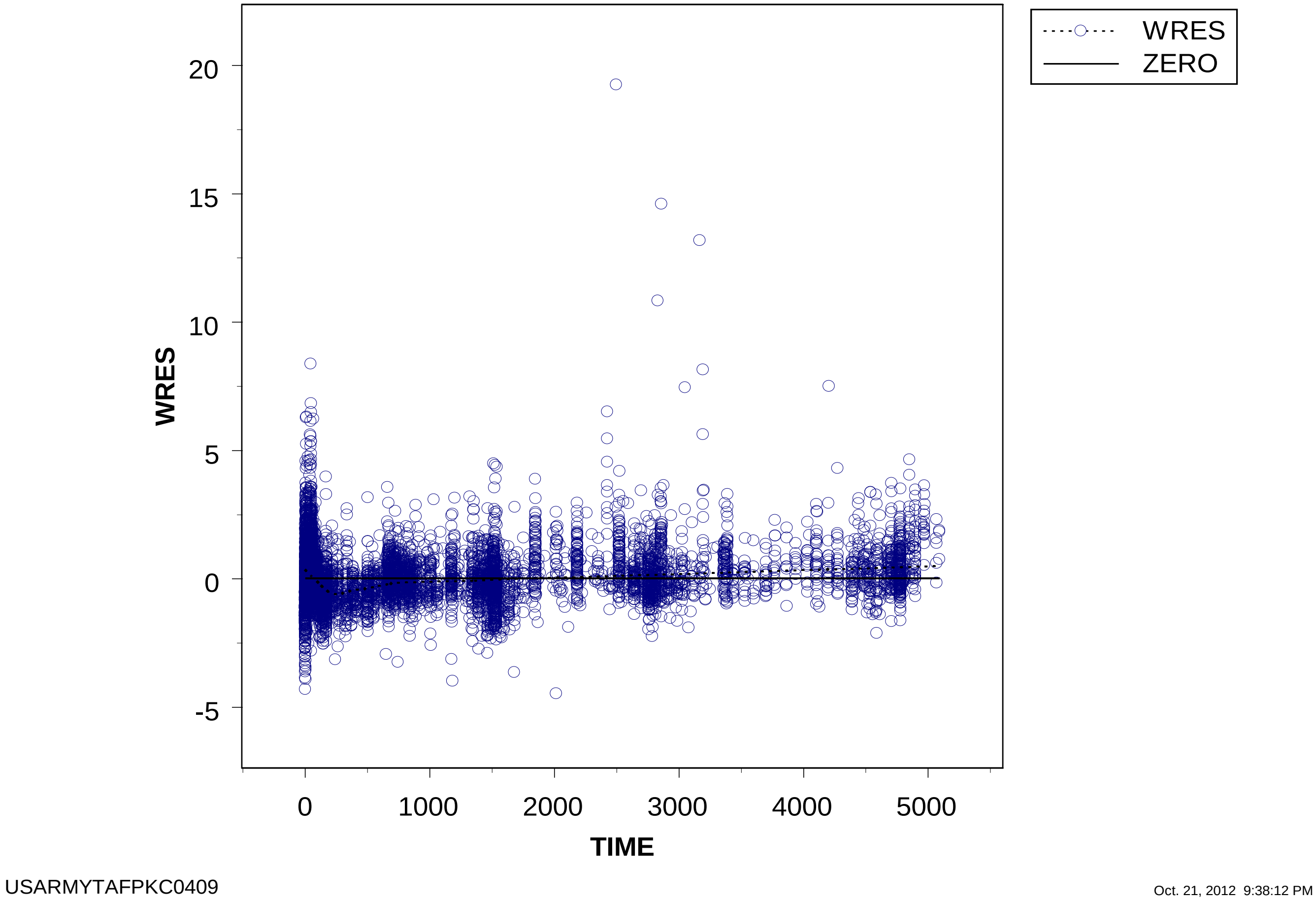

Note: Time in hours

End-of-Text Figure 16-8 Plot of Weighted Residuals (WRES) vs. Time after Dose (Final PK Model)

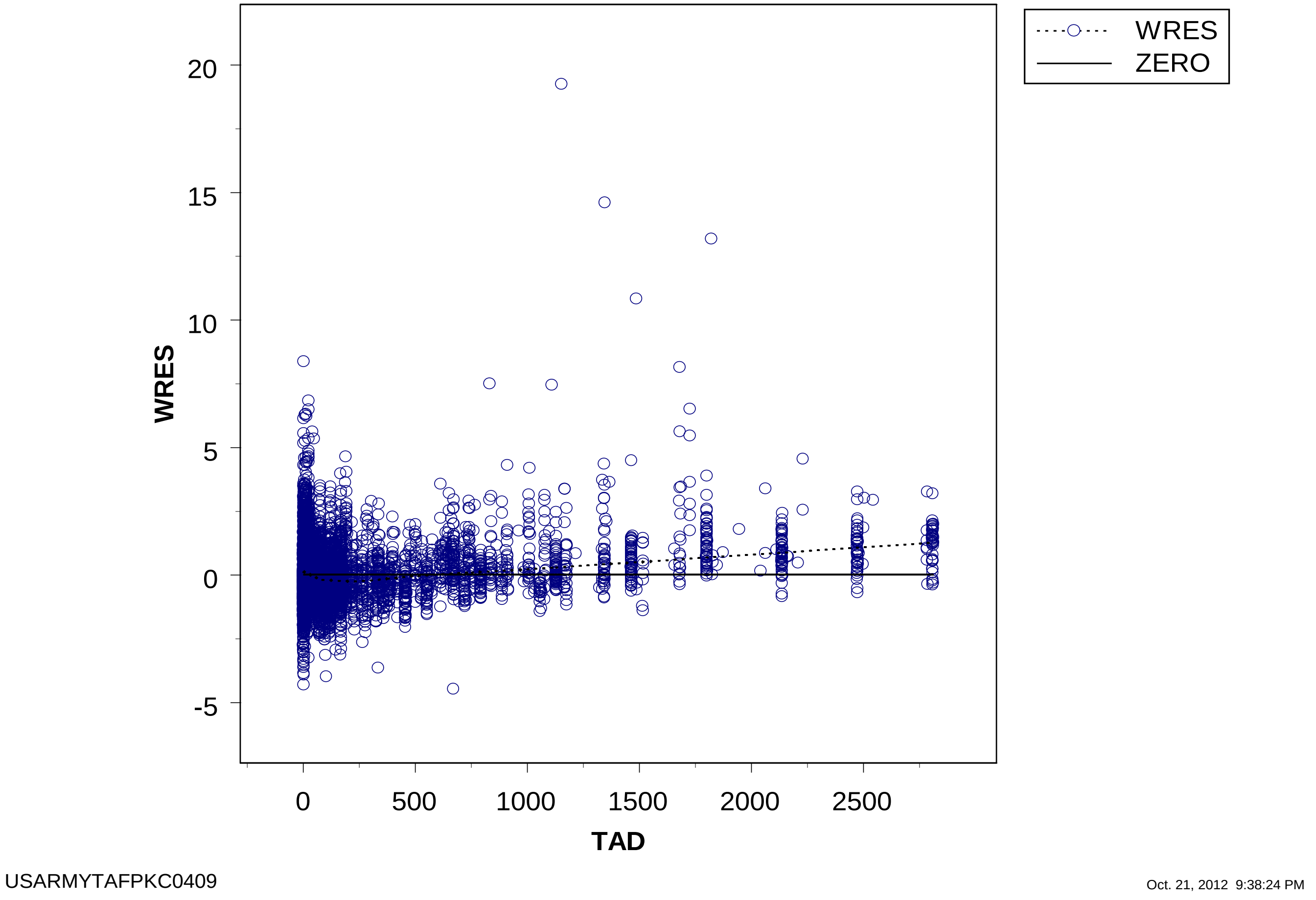

Note: Time after dose (TAD) in hours

End-of-Text Figure 16-9 Plot of Weighted Residuals (WRES) vs. ID (Final PK Model)

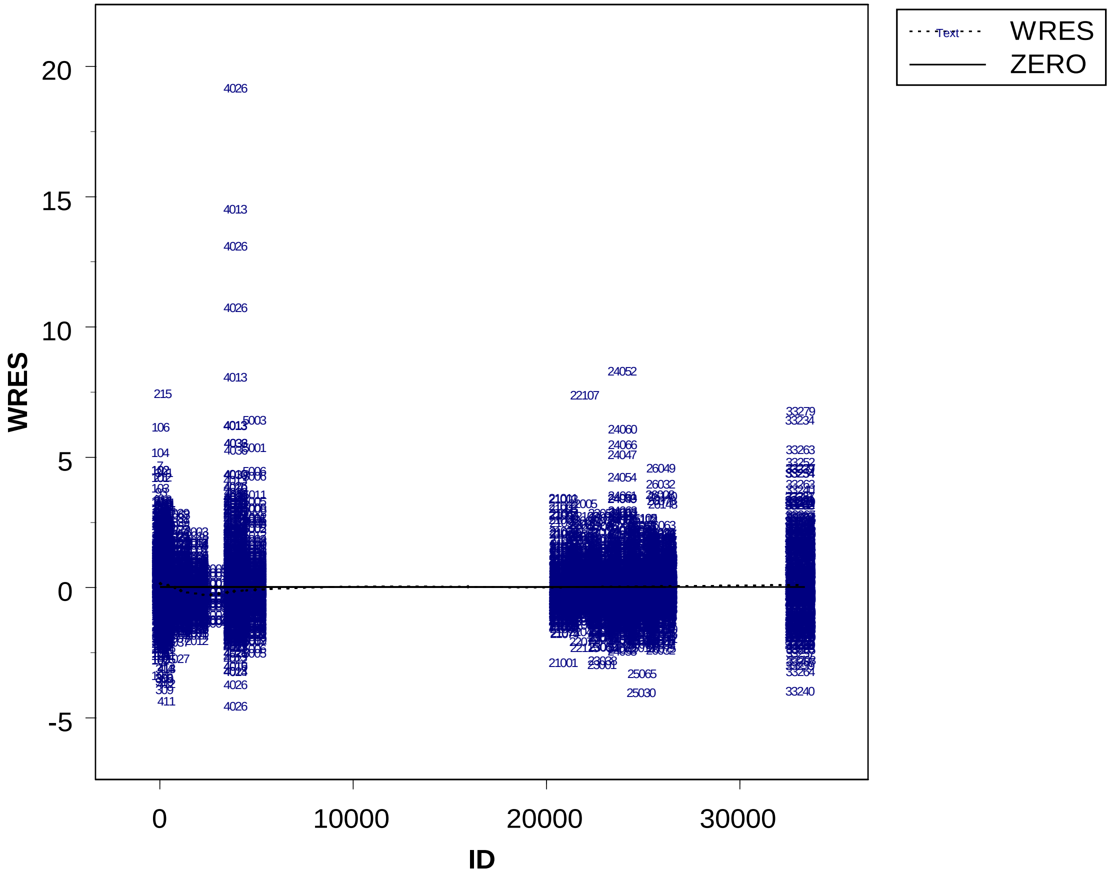

USARMYTAFPKC0409

Oct. 21, 2012 9:38:43 PM

End-of-Text Figure 16-10 Plot of Individual Weighted Residuals (IWRES) vs. Individual Predicted (IPRED) Tafenoquine Concentrations (Final PK Model)

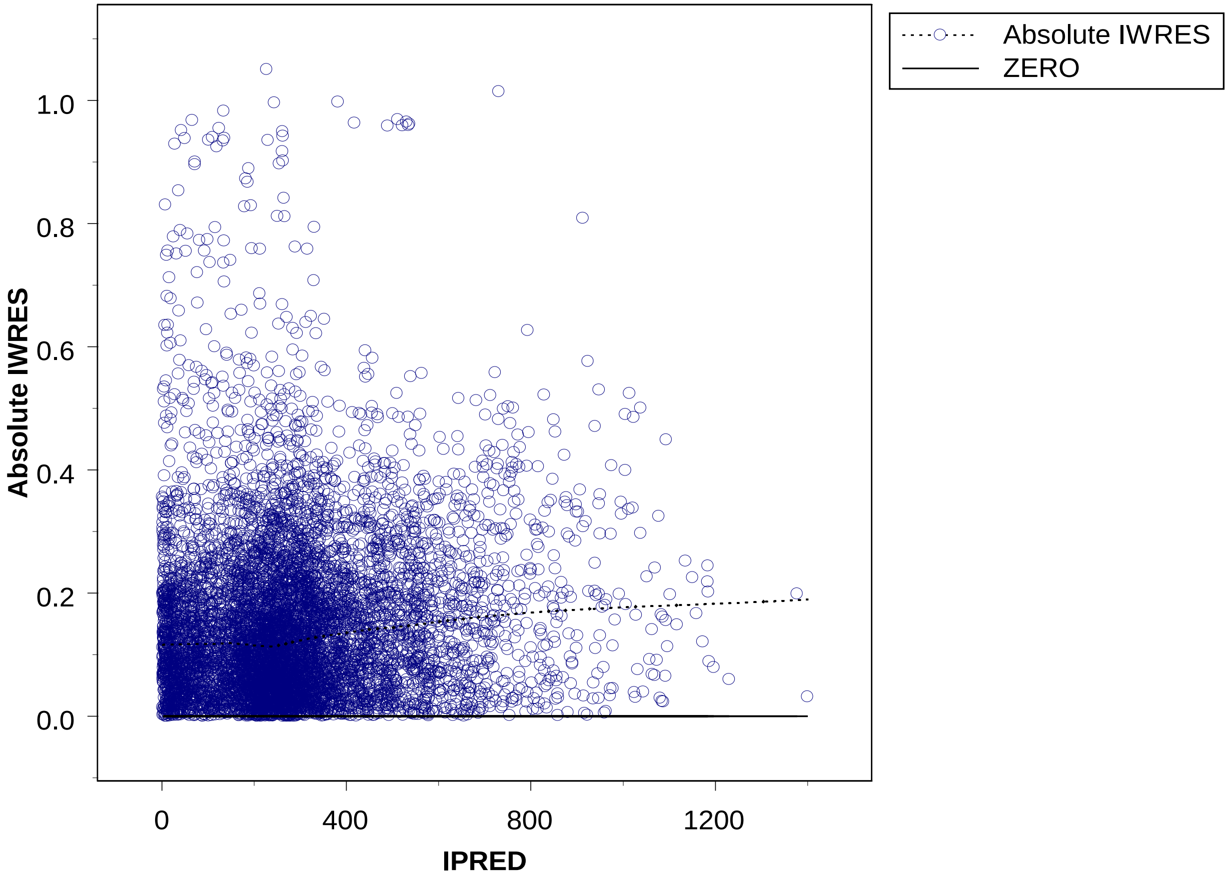

USARMYTAFPKC0409

Oct. 21, 2012 9:38:59 PM

End-of-Text Figure 16-11 Plot of Predicted Tafenoquine Concentrations vs. Observed Tafenoquine Concentrations (Final PK Model)

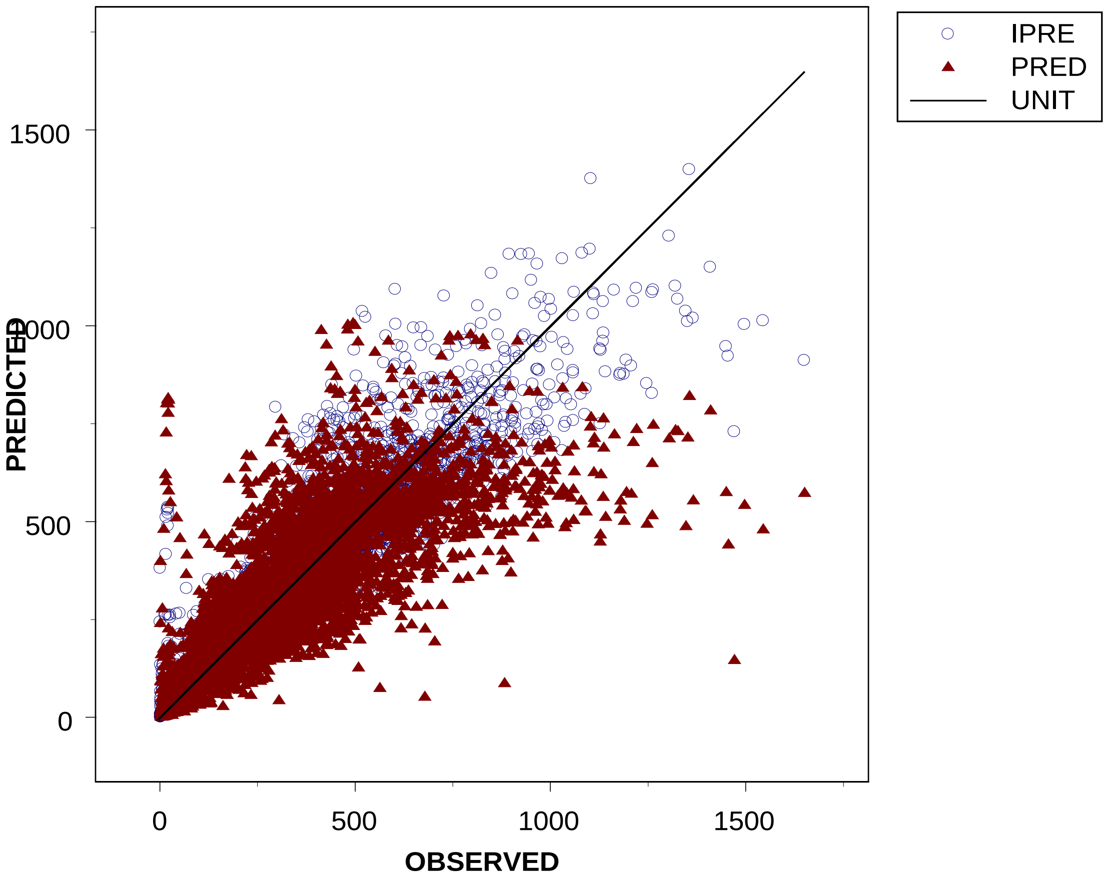

USARMYTAFPKC0409

Oct. 21, 2012 9:39:58 PM

End-of-Text Figure 16-12 Plot of Conditional Weighted Residuals (CWRES) vs. Population Predicted (PRED) Tafenoquine Concentrations (Final PK Model)

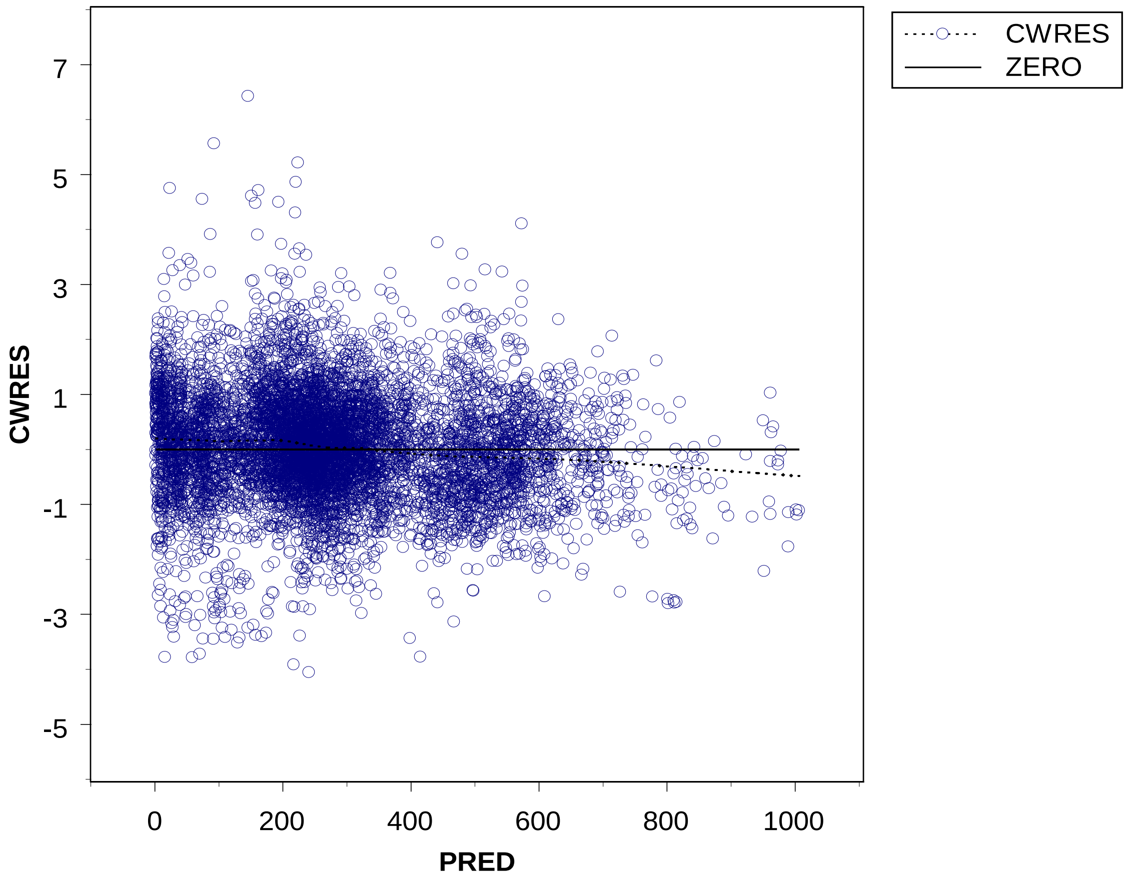

USARMYTAFPKC0409

Oct. 21, 2012 9:40:11 PM

End-of-Text Figure 16-13   Plot of Conditional Weighted Residuals (CWRES) vs. Time (Final PK Model)

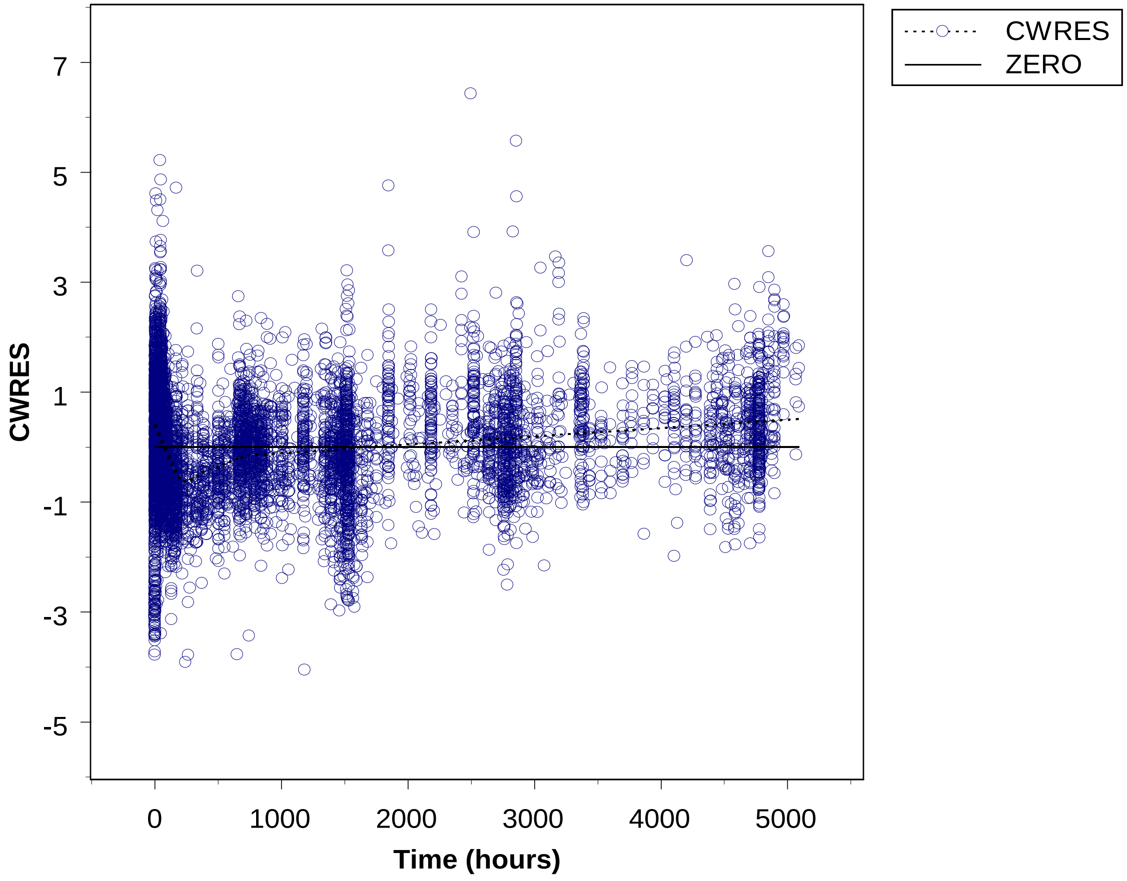

USARMYTAFPKC0409

Oct. 21, 2012 9:40:24 PM

End-of-Text Figure 16-14 Plot of DV, PRED, and IPRED vs. Time (Linear Scale) (Final PK Model)

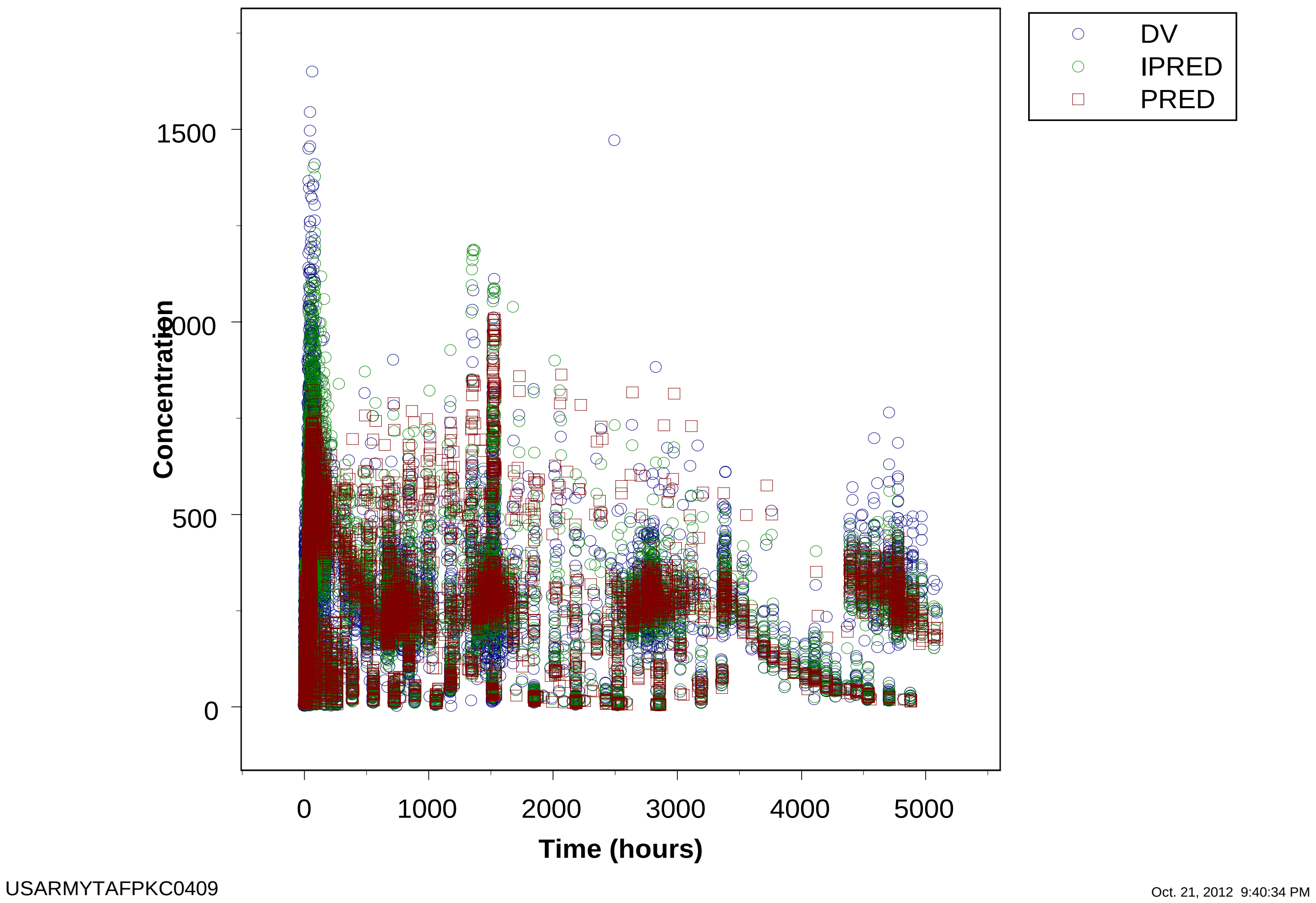

Note: Concentration in ng/mL

End-of-Text Figure 16-15 Plot of DV, PRED, and IPRED vs. Time (Semi-Logarithmic Scale) (Final PK Model)

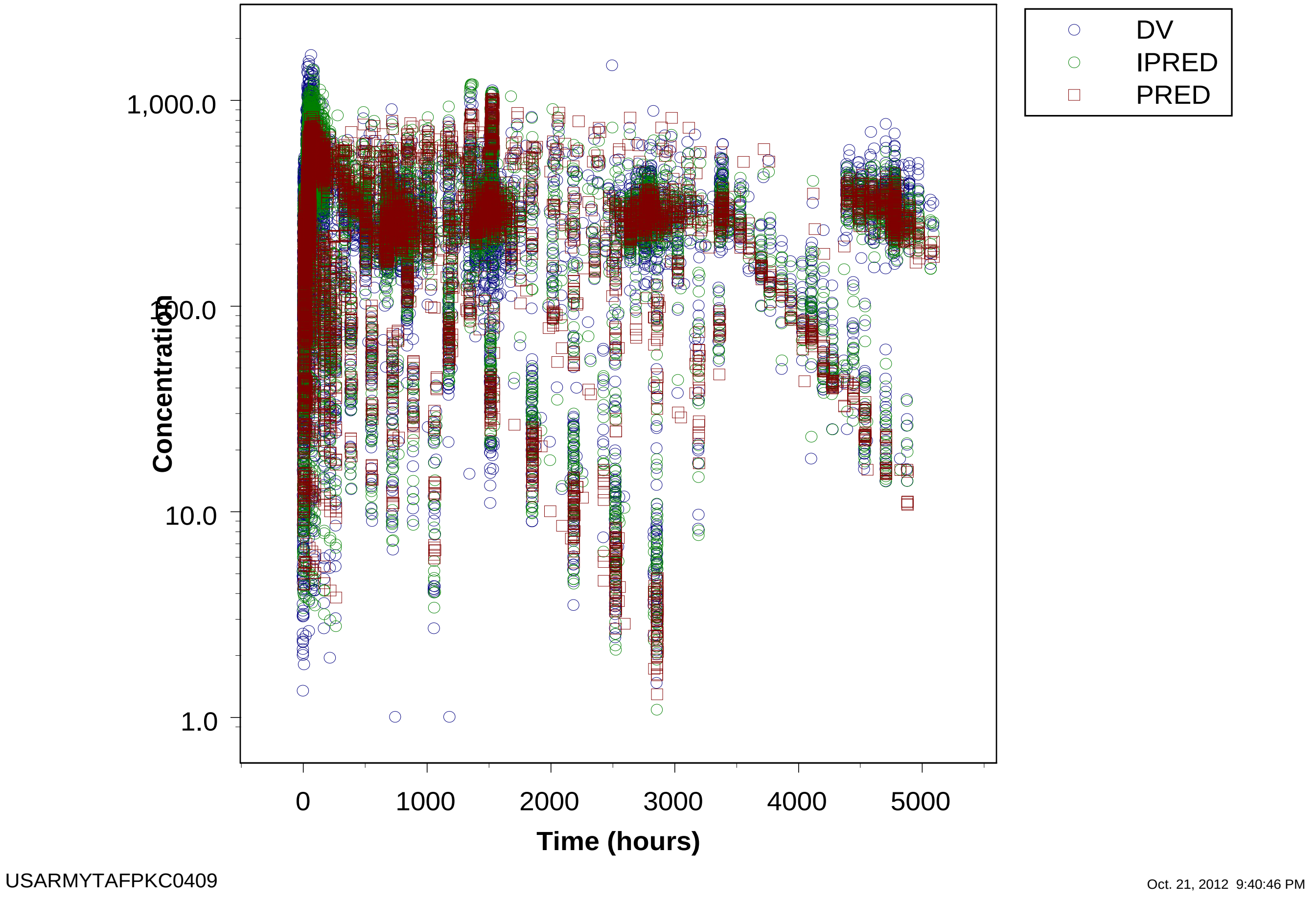

Note: Concentration in ng/mL

End-of-Text Figure 16-16 Plot of DV, PRED, and IPRED vs. Time after Dose (Linear Scale) (Final PK Model)

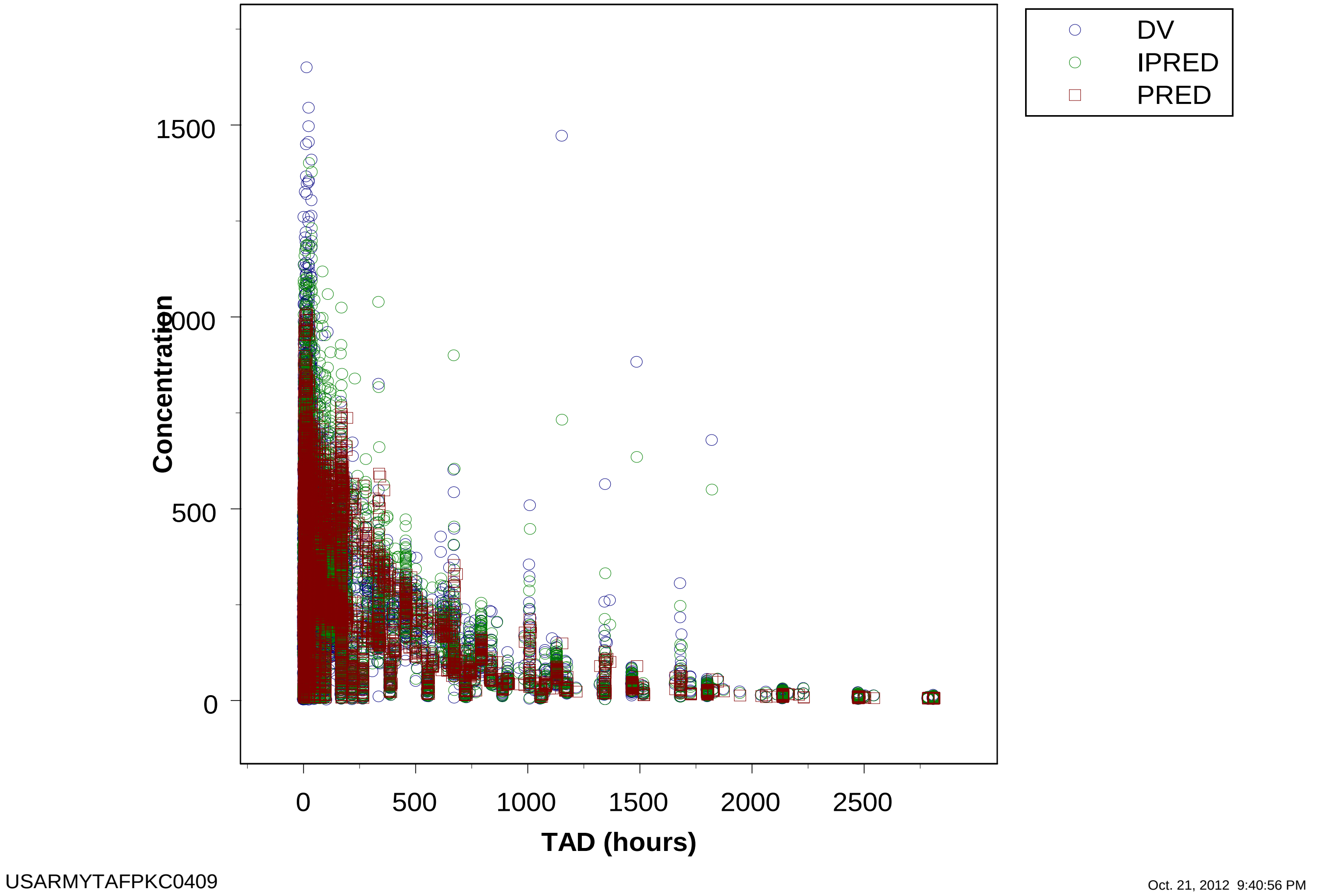

Note: Concentration in ng/mL

End-of-Text Figure 16-17 Plot of DV, PRED, and IPRED vs. Time after Dose (Semi-Logarithmic Scale) (Final PK Model)

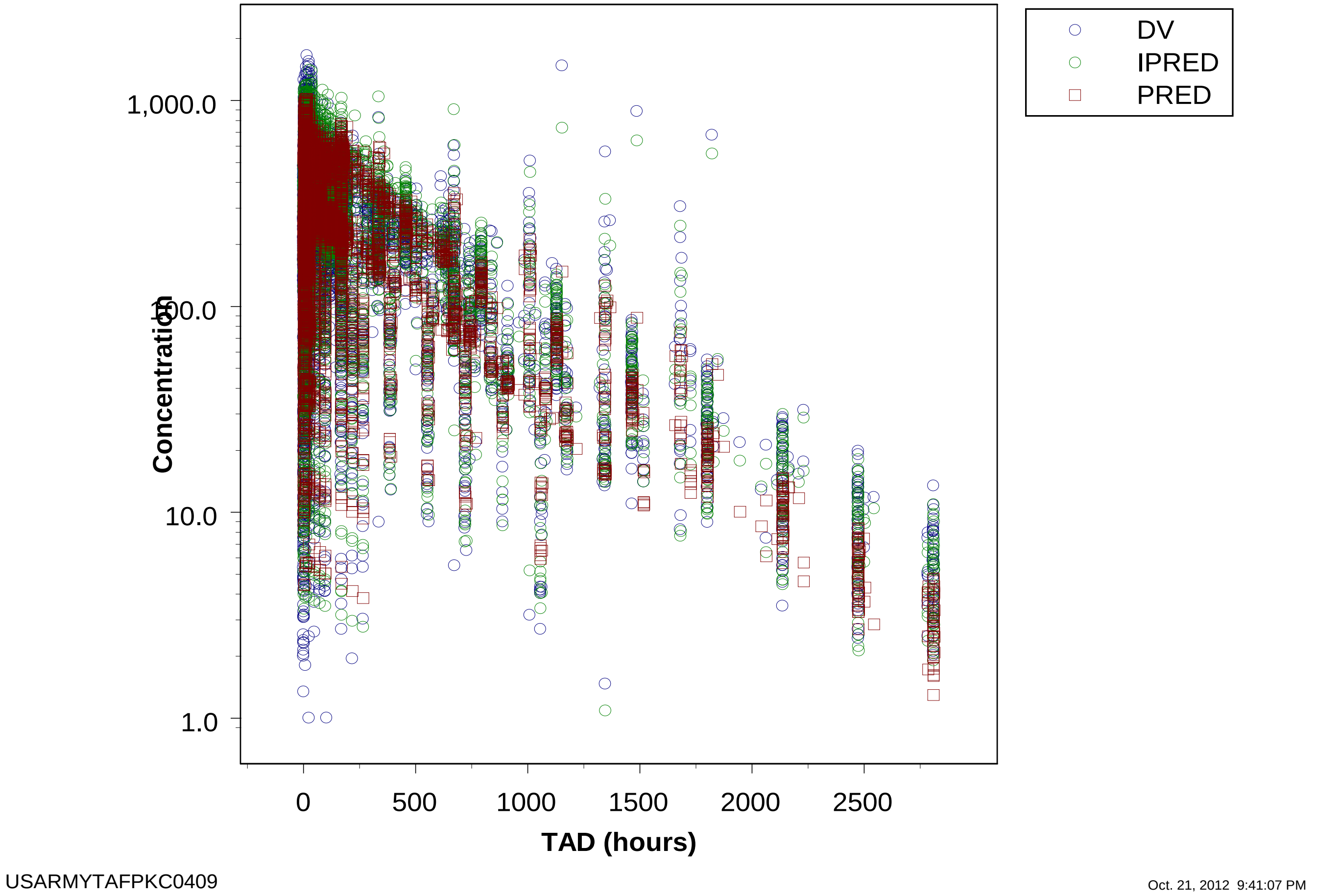

Note: Concentration in ng/mL

End-of-Text Figure 16-18 Plot of CWRES vs. Covariates

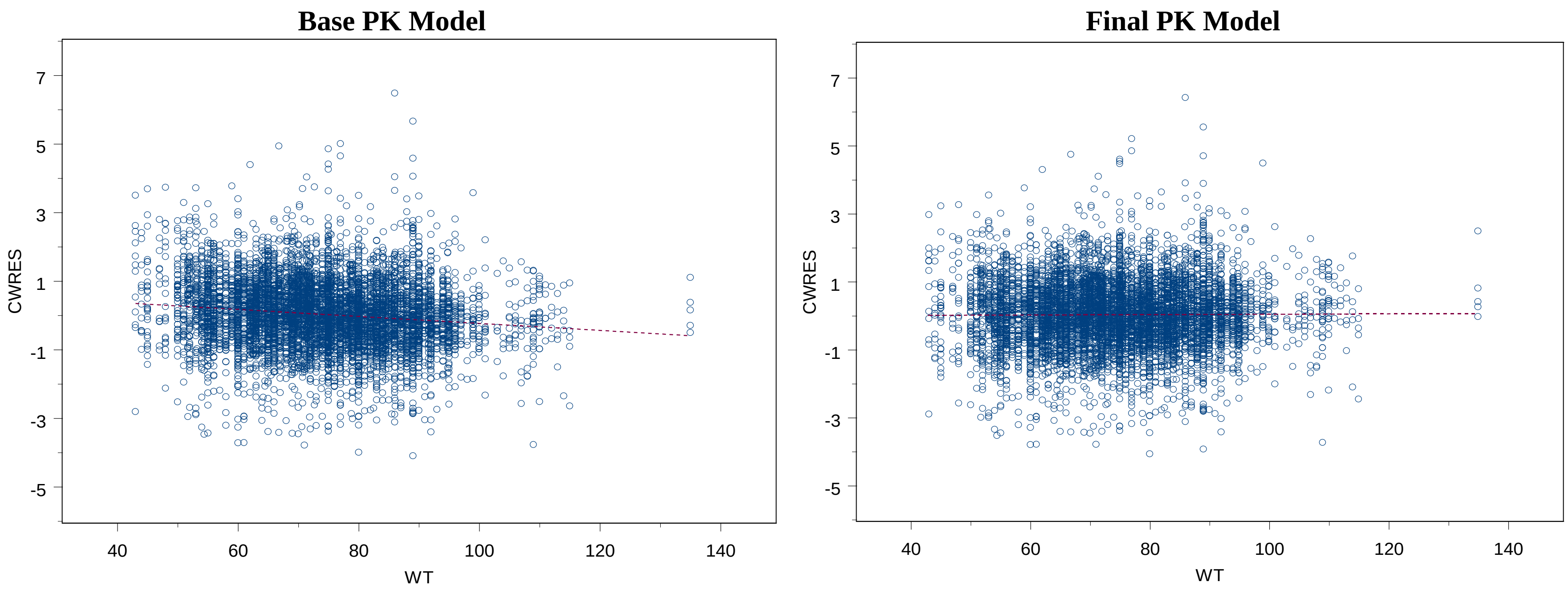

End-of-Text Figure 8-20 Plot of CWRES vs. Covariates (Cont'd)

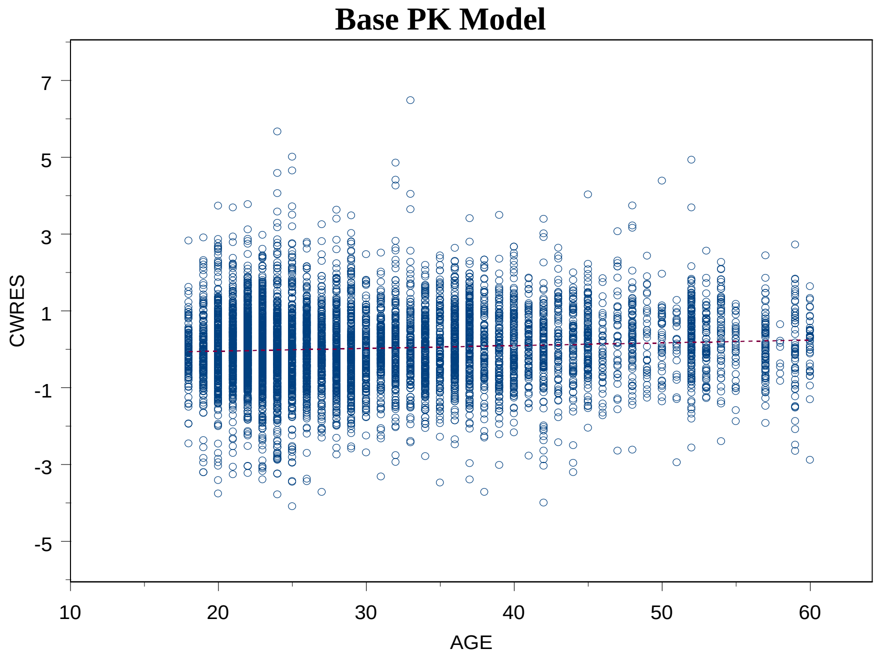

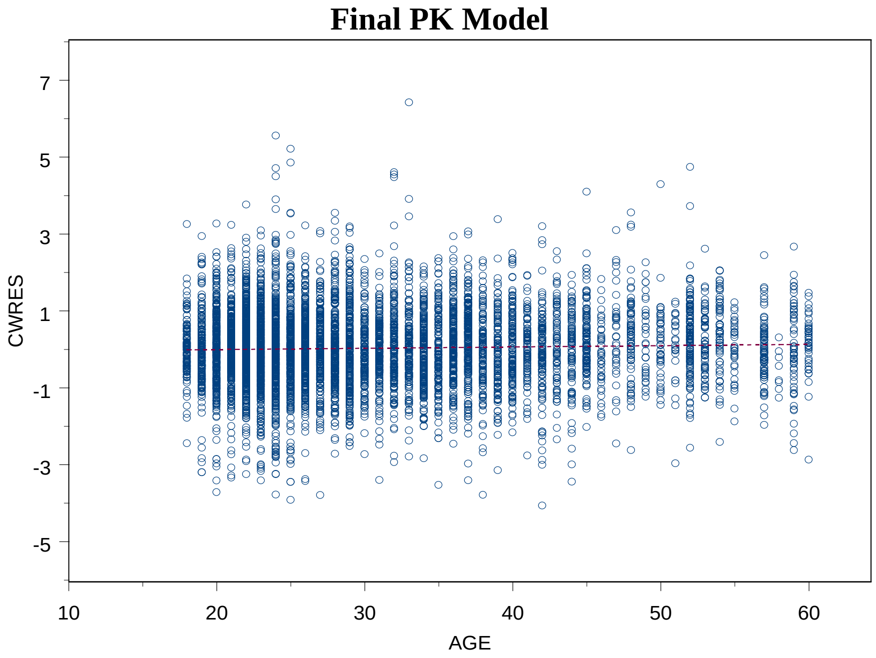

End-of-Text Figure 8-20 Plot of CWRES vs. Covariates (Final PK Model) (Cont'd)

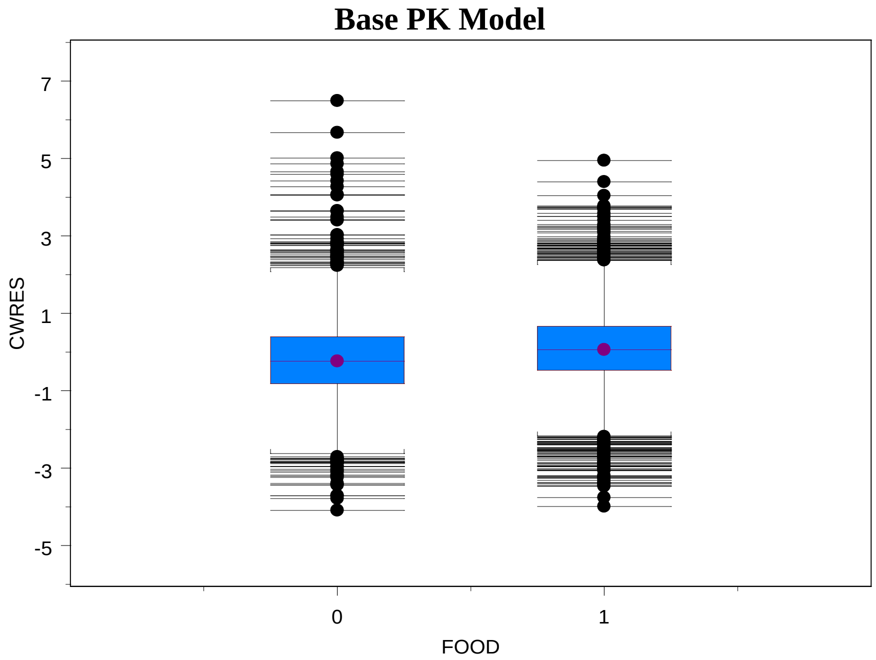

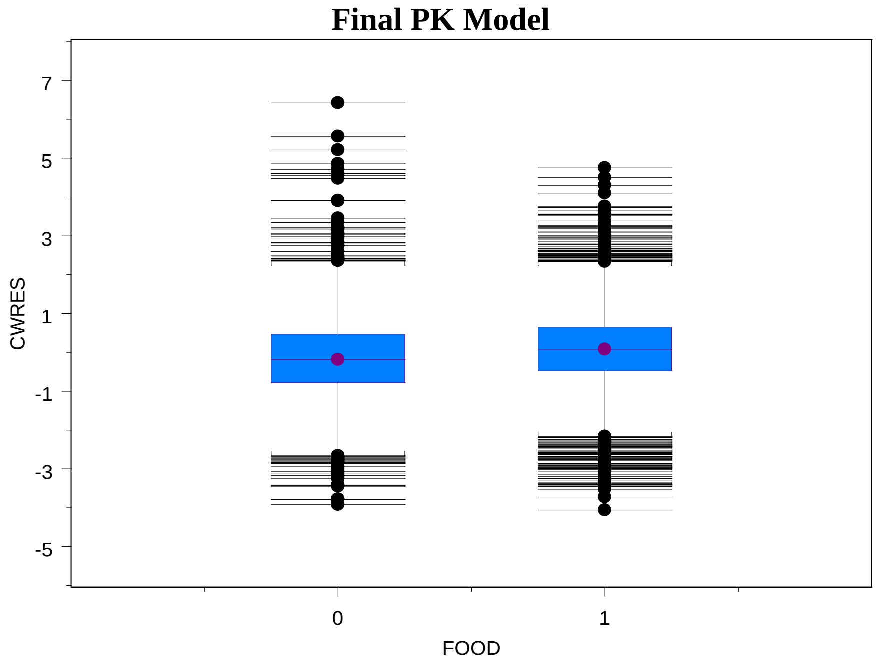

End-of-Text Figure 16-19 Distribution of Individual Estimate of ETA (Final PK Model)

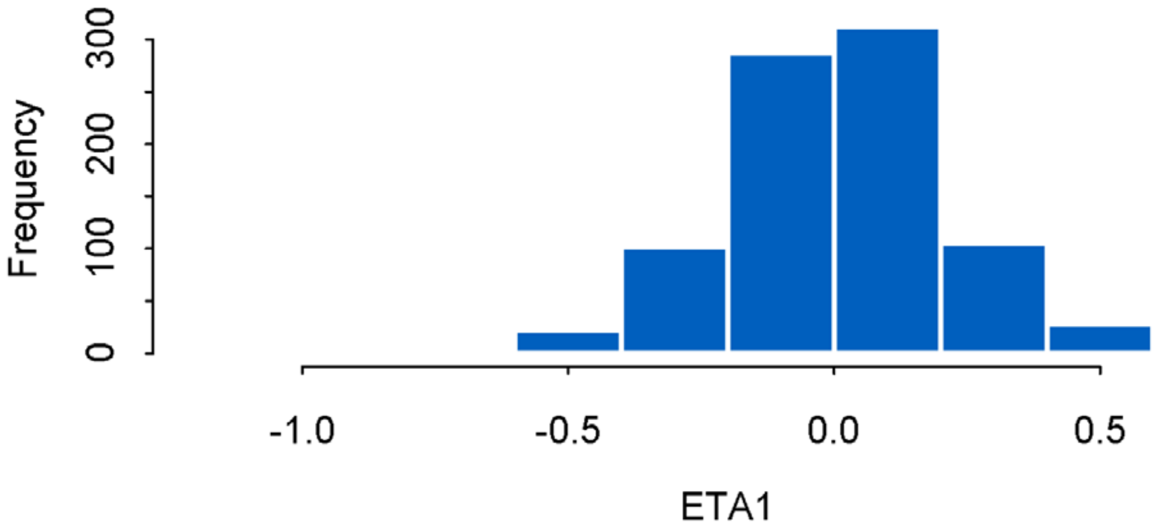

ETA1 on CL

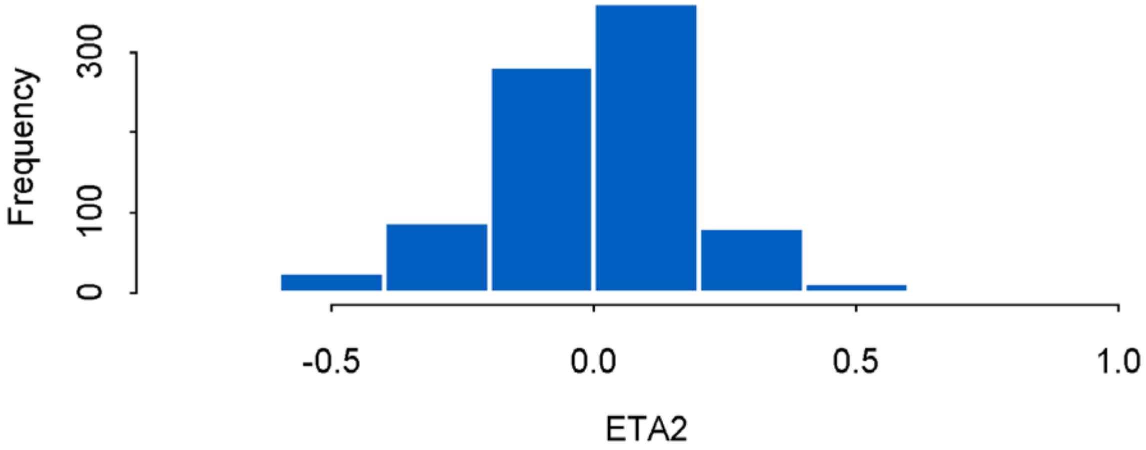

ETA2 on V2

End-of-Text Figure 8-21 Distribution of Individual Estimate of ETA (Final PK Model) (Cont'd)

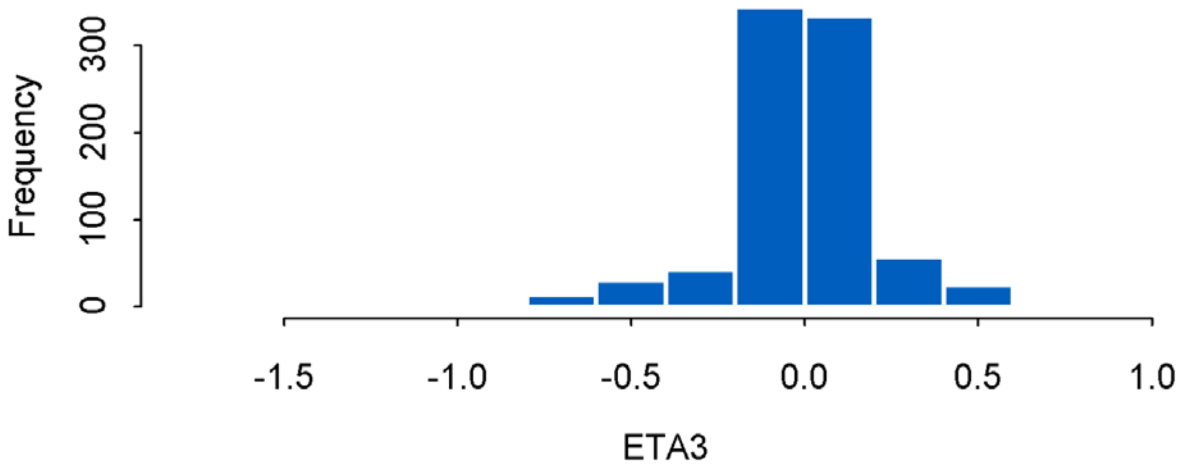

ETA3 on KA

End-of-Text Figure 16-20 Plot of Correlation Matrix of ETA (Final PK Model)

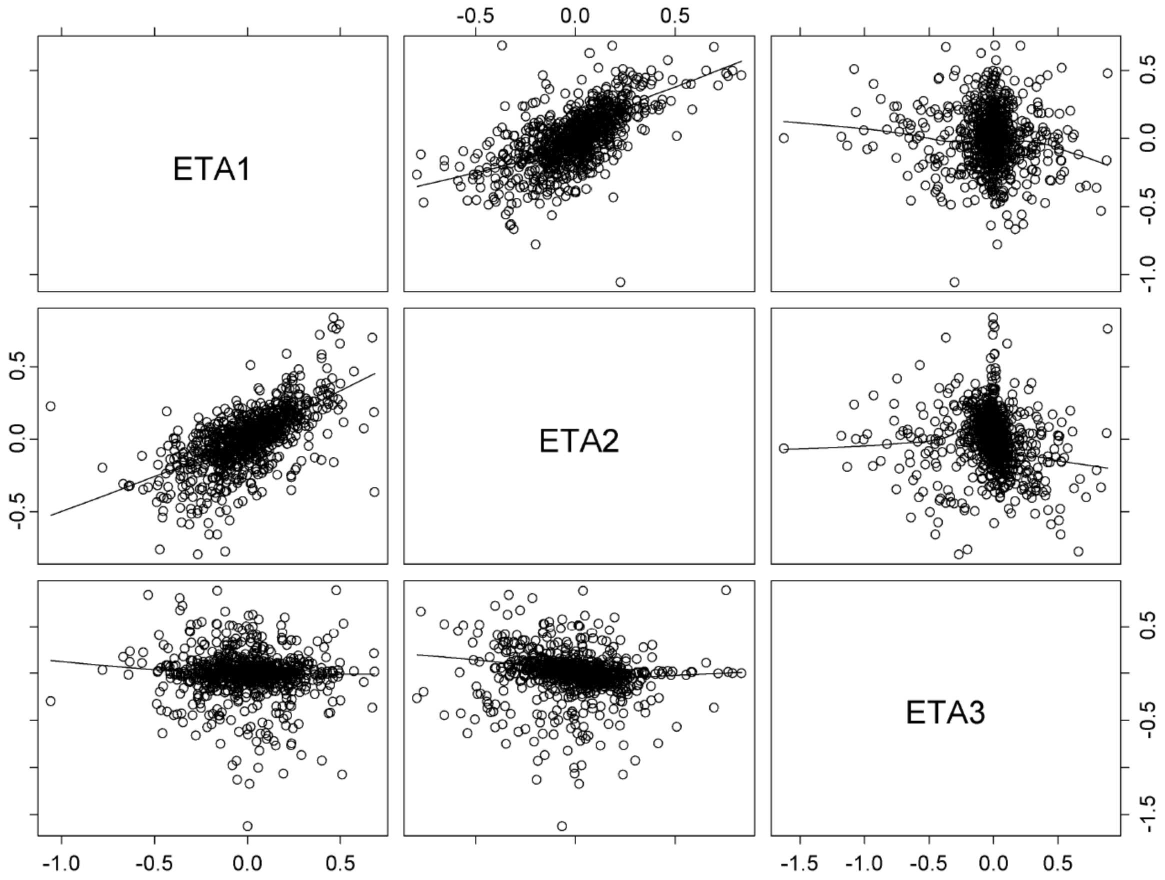

End-of-Text Figure 16-21 Plot of ETA vs. Covariates

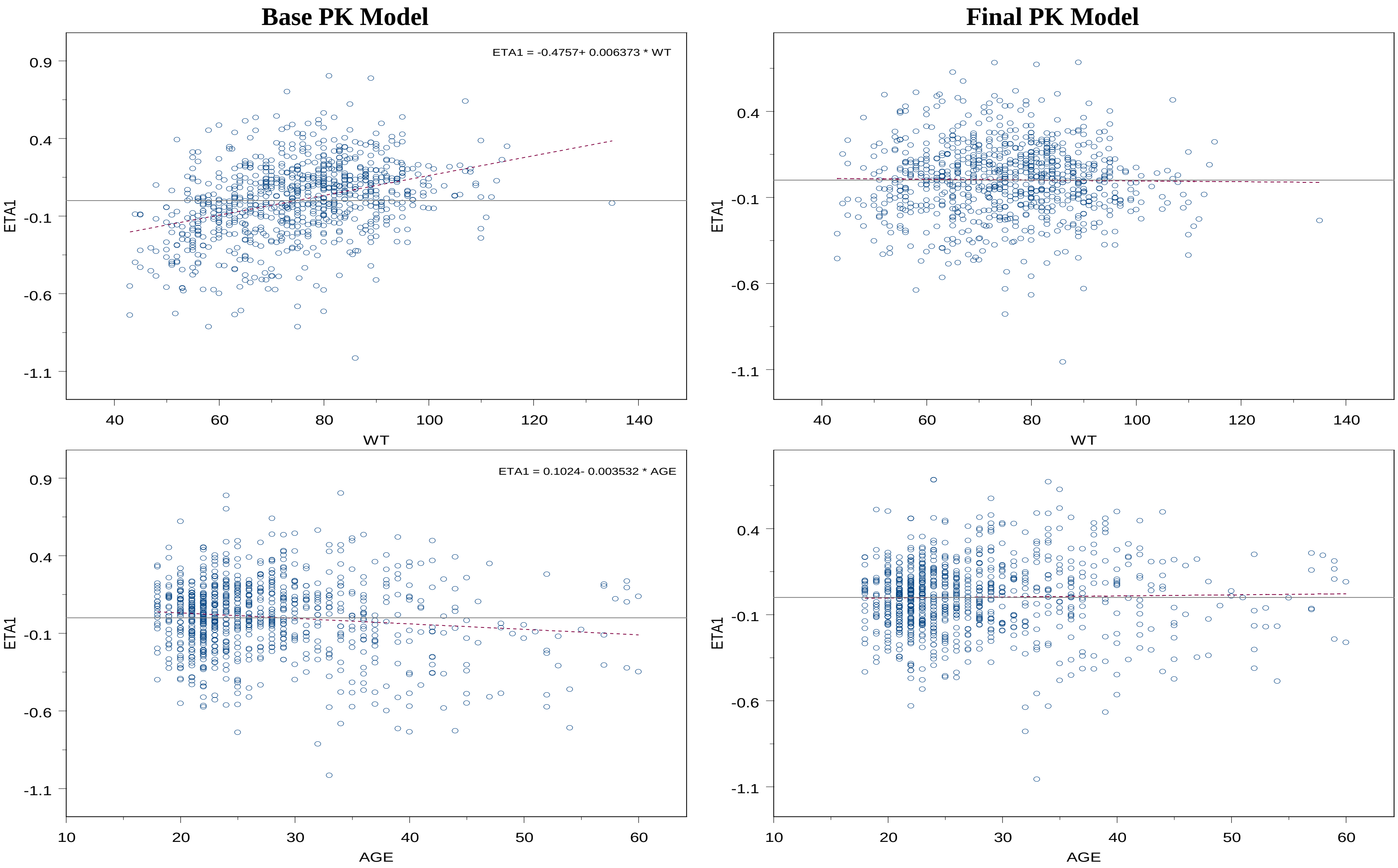

End-of-Text Figure 8-23 Plot of ETA vs. Covariates (Cont'd)

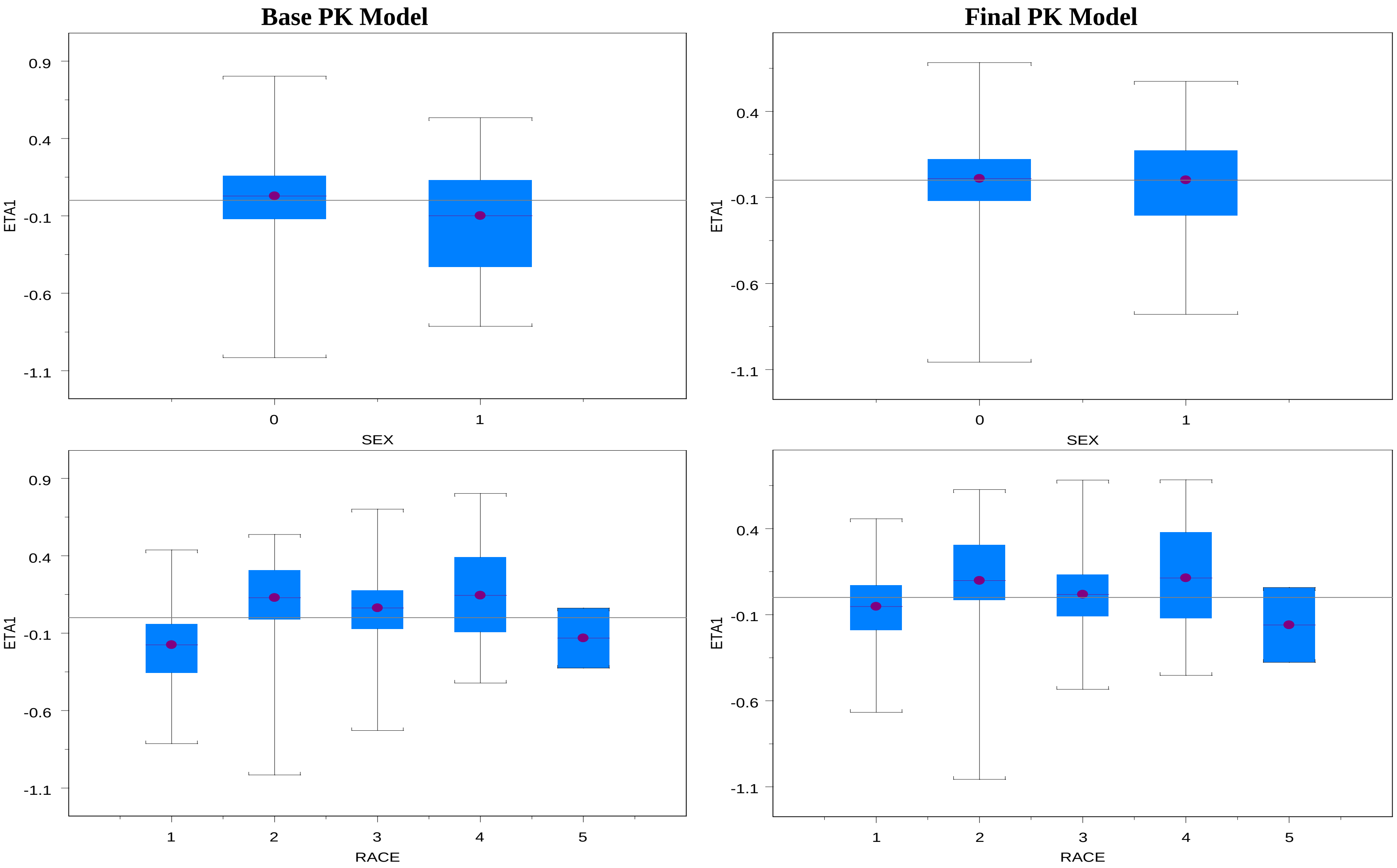

End-of-Text Figure 8-23 Plot of ETA vs. Covariates (Cont'd)

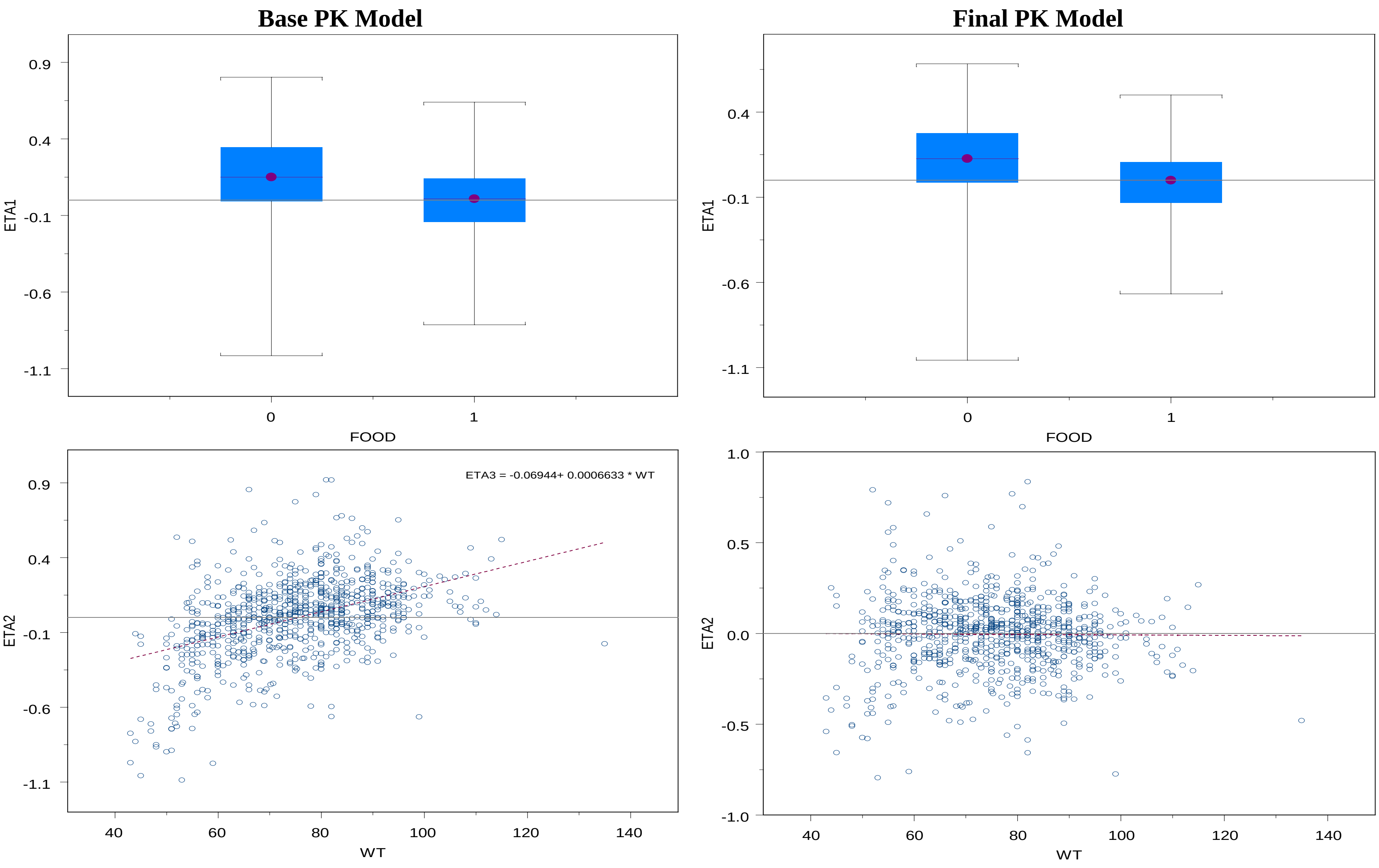

End-of-Text Figure 8-23 Plot of ETA vs. Covariates (Cont'd)

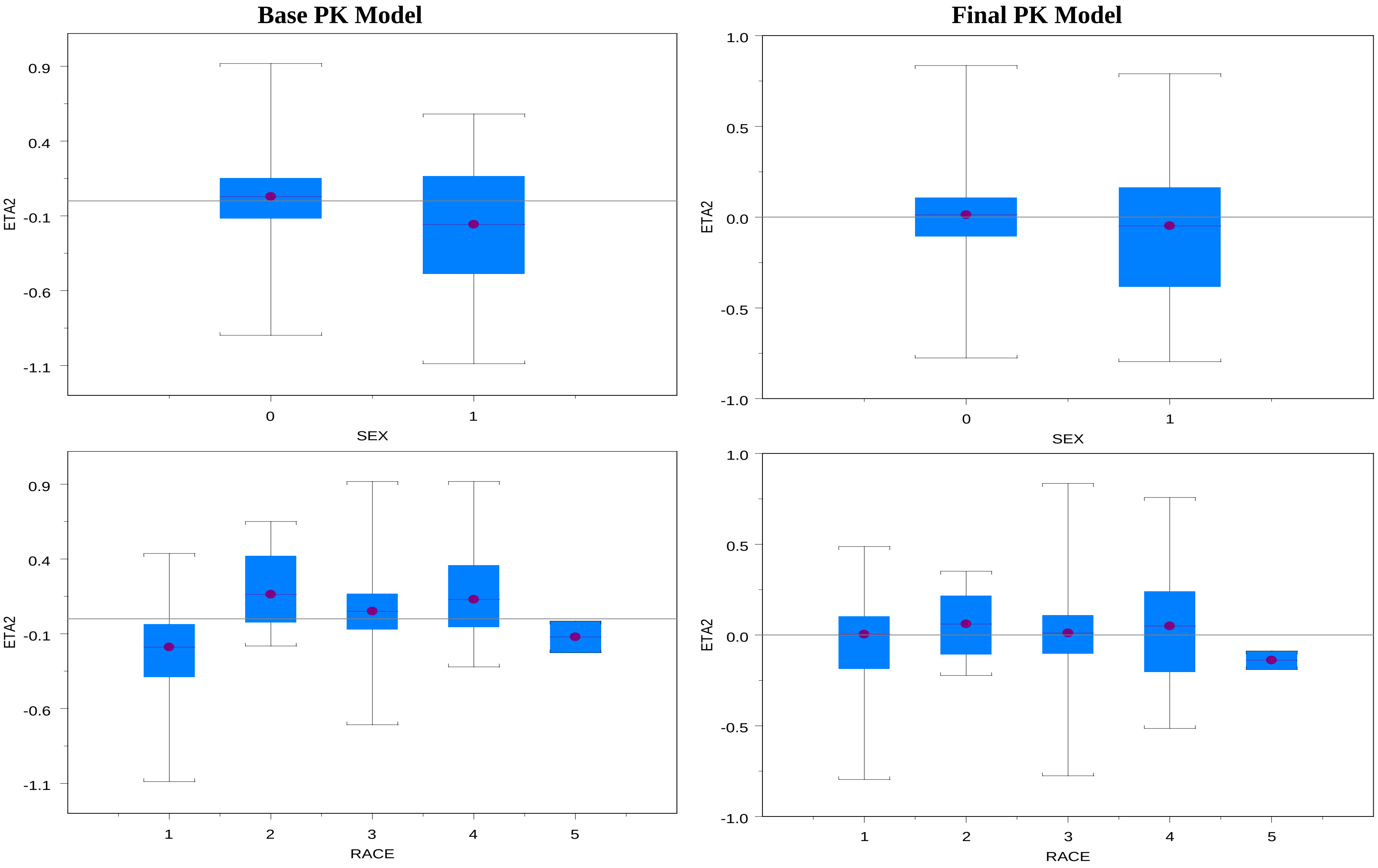

End-of-Text Figure 8-23 Plot of ETA vs. Covariates (Cont'd)

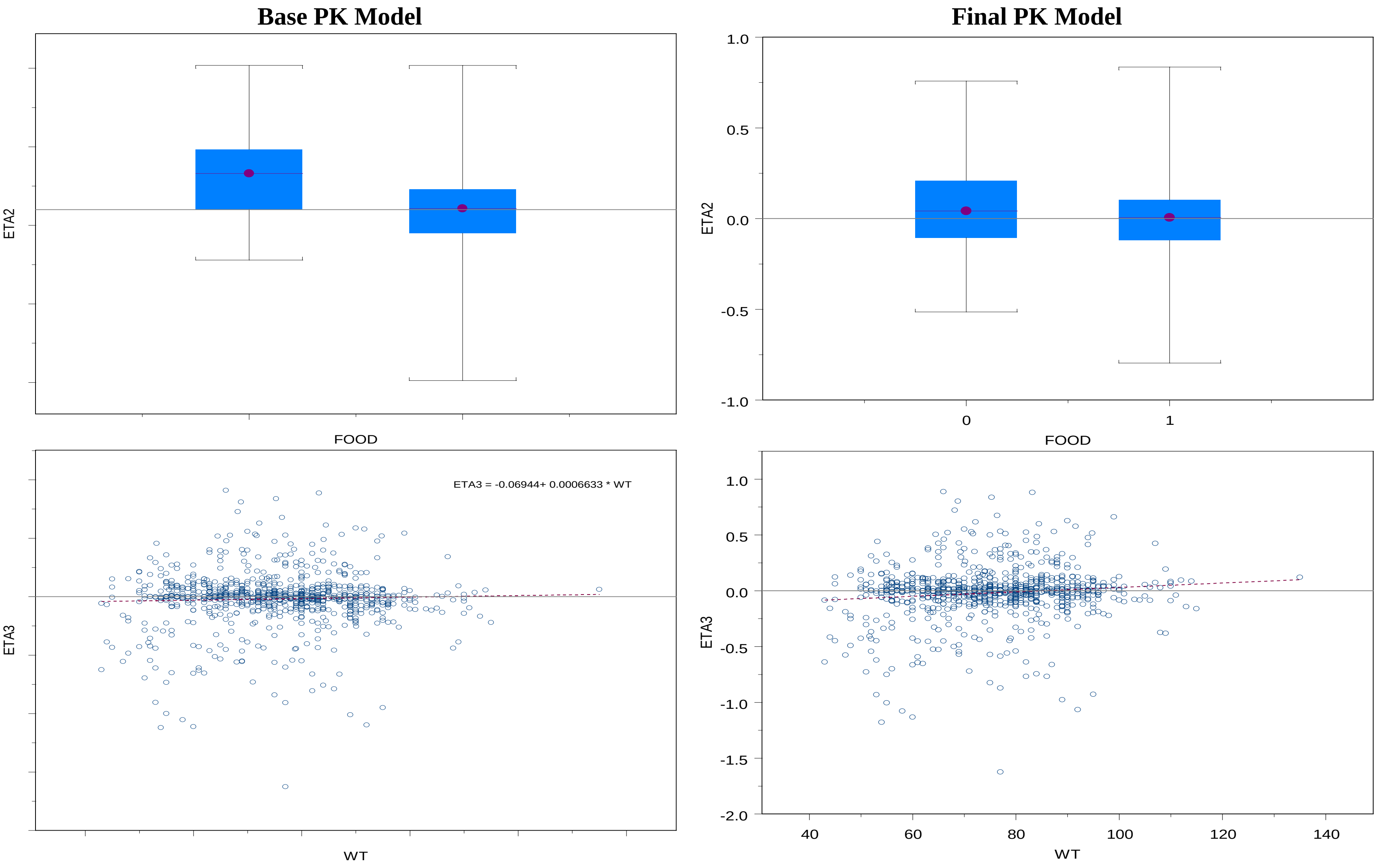

End-of-Text Figure 8-23 Plot of ETA vs. Covariates (Cont'd)

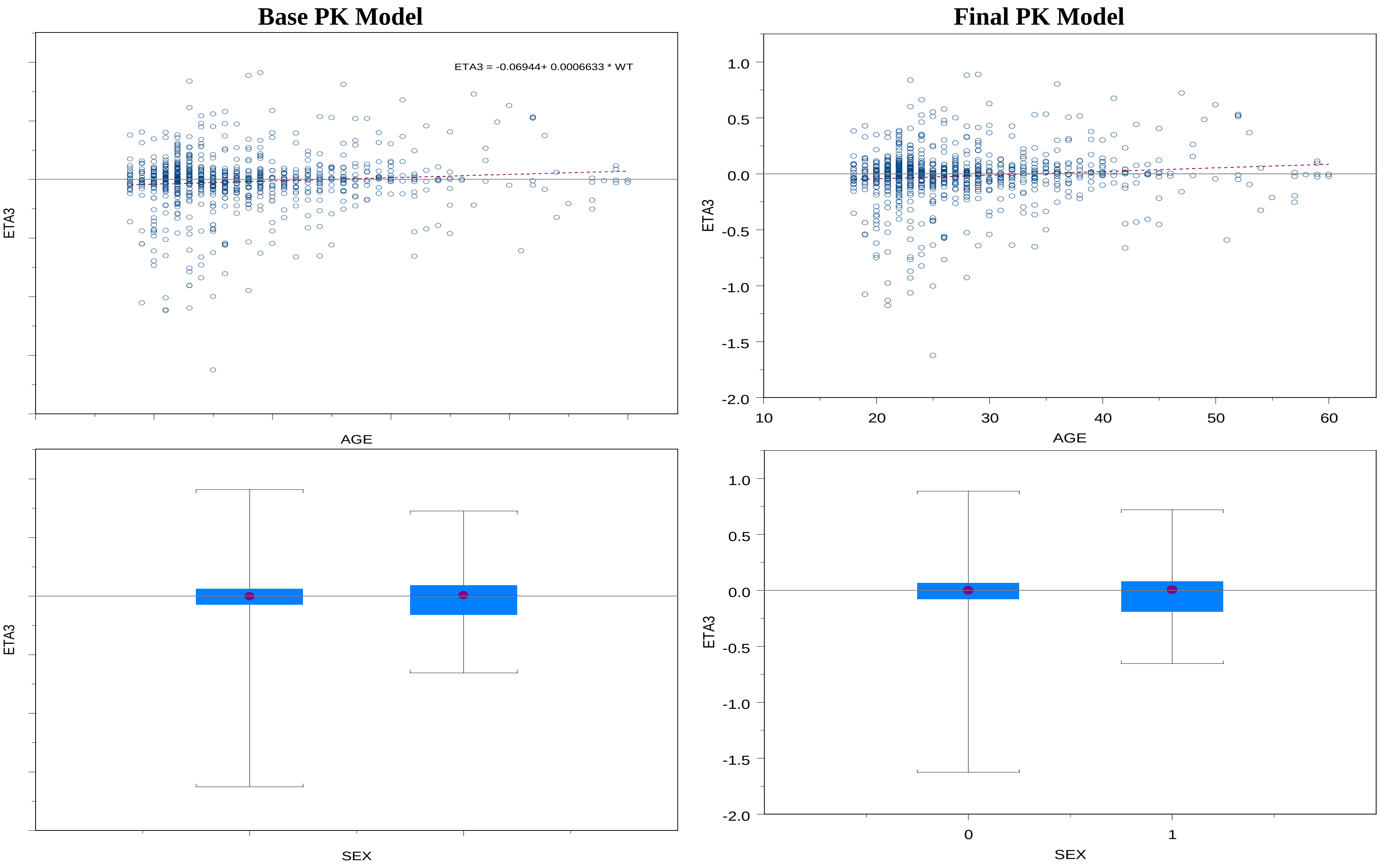

End-of-Text Figure 8-23 Plot of ETA vs. Covariates (Cont'd)

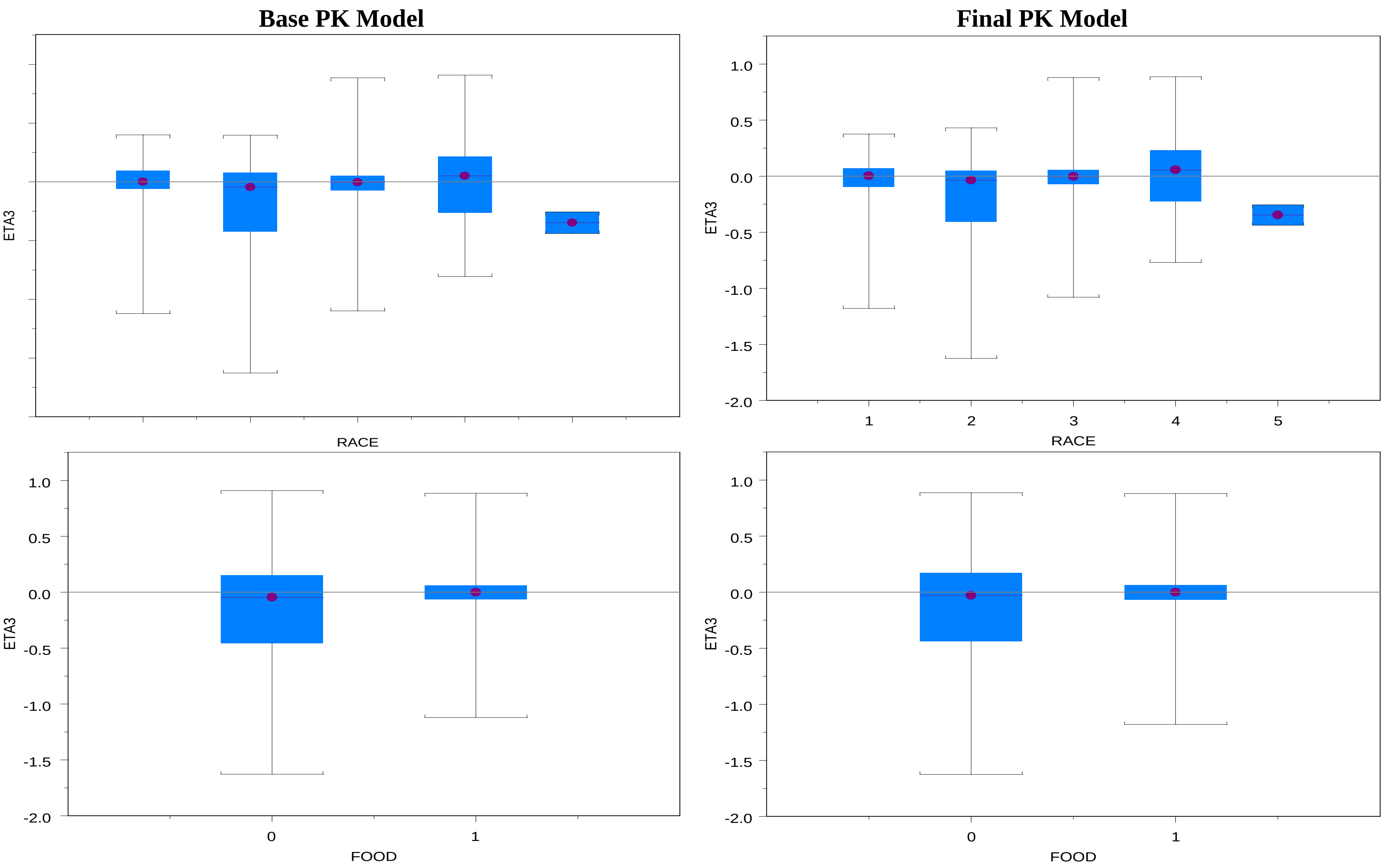

Supplement: Supplementary file 1 — Additional file 1. Population Pharmacokinetic Modeling Report. [file 12936_2017_1862_MOESM1_ESM.pdf]
